# Placebo rates in randomized clinical trials of ulcerative colitis: an individual patient data meta-analysis

**DOI:** 10.1093/ecco-jcc/jjaf191

**Published:** 2025-11-09

**Authors:** Virginia Solitano, Malcolm Hogan, Siddharth Singh, Silvio Danese, Laurent Peyrin-Biroulet, Sudheer Vuyyuru, John K Macdonald, Guangyong Zou, Yuhong Yuan, Bruce E Sands, Remo Panaccione, Brian G Feagan, Jurij Hanzel, Rocio Sedano, Parambir Dulai, Neeraj Narula, Christopher Ma, Vipul Jairath

**Affiliations:** Division of Gastroenterology, Western University, London, Ontario N6A 3K7, Canada; Division of Gastroenterology and Gastrointestinal Endoscopy, IRCCS Ospedale San Raffaele, Università Vita-Salute San Raffaele, Milan 20132, Italy; Alimentiv Inc, London, Ontario N6A 5B6, Canada; Division of Gastroenterology, Department of Medicine, University of California, San Diego, La Jolla, CA 92037, United States; Division of Gastroenterology and Gastrointestinal Endoscopy, IRCCS Ospedale San Raffaele, Università Vita-Salute San Raffaele, Milan 20132, Italy; Department of Gastroenterology, Nancy University Hospital, Vandœuvre-lès-Nancy F-54500, France; INFINY Institute, Nancy University Hospital, Vandœuvre-lès-Nancy F-54500, France; Groupe Hospitalier Privé Ambroise Paré – Hartmann Paris IBD Center, Neuilly sur Seine 92200, France; Division of Gastroenterology, Western University, London, Ontario N6A 3K7, Canada; Alimentiv Inc, London, Ontario N6A 5B6, Canada; Alimentiv Inc, London, Ontario N6A 5B6, Canada; Alimentiv Inc, London, Ontario N6A 5B6, Canada; Department of Epidemiology and Biostatistics, Western University, London, Ontario N6A 5C1, Canada; Lawson Health Research Institute, London, Ontario N6A 4V2, Canada; Dr. Henry D. Janowitz Division of Gastroenterology, Icahn School of Medicine at Mount Sinai, New York, NY 10029, United States; Division of Gastroenterology & Hepatology, University of Calgary, Calgary, Alberta T2N 4Z6, Canada; Division of Gastroenterology, Western University, London, Ontario N6A 3K7, Canada; Alimentiv Inc, London, Ontario N6A 5B6, Canada; Department of Epidemiology and Biostatistics, Western University, London, Ontario N6A 5C1, Canada; Alimentiv Inc, London, Ontario N6A 5B6, Canada; Department of Gastroenterology, University Medical Centre Ljubljana, Faculty of Medicine, University of Ljubljana, Ljubljana 1000, Slovenia; Division of Gastroenterology, Western University, London, Ontario N6A 3K7, Canada; Alimentiv Inc, London, Ontario N6A 5B6, Canada; Division of Gastroenterology, Northwestern University, Chicago, IL 60611, United States; Department of Medicine, Division of Gastroenterology, Farncombe Family Digestive Health Research Institute, McMaster University, Hamilton, Ontario L8S 4K1, Canada; Alimentiv Inc, London, Ontario N6A 5B6, Canada; Division of Gastroenterology & Hepatology, University of Calgary, Calgary, Alberta T2N 4Z6, Canada; Department of Community Health Sciences, University of Calgary, Calgary, Alberta T2N 4N1, Canada; Division of Gastroenterology, Western University, London, Ontario N6A 3K7, Canada; Alimentiv Inc, London, Ontario N6A 5B6, Canada; Department of Epidemiology and Biostatistics, Western University, London, Ontario N6A 5C1, Canada

**Keywords:** placebo, clinical trials, inflammatory bowel disease, ulcerative colitis

## Abstract

**Background and Aims:**

We assessed placebo rates and associated factors using individual patient data (IDP) from randomized clinical trials (RCTs) in ulcerative colitis (UC).

**Methods:**

We conducted an IPD meta-analysis using Vivli and Yale University Open Data Access data-sharing platforms. Phase 2 and 3 RCTs of advanced biologics in adults with moderate-to-severe UC published since 2010 were included. Pooled placebo rates and 95% CIs were estimated using one- and two-stage meta-analytical approaches. Significant patient-level factors (*P* < .05) were identified using regression analyses. Primary outcomes were clinical response and remission.

**Results:**

Data were available for 1703 patients from nine studies. For induction trials, overall placebo response and remission rates were 33% (95% CI 29%-38%) and 9% (95% CI 7%-11%). Overall placebo response and remission rates in maintenance trials were 28% (95% CI 18%-41%) and 14% (95% CI 9%-20%). A lower body mass index reduced the odds of placebo response and remission, while higher baseline albumin levels and left-sided (compared to extensive) UC increased the odds of these outcomes. A 1-point increase in the Mayo Clinic Score (MCS) and adapted MCS was associated with a 26% and 27% reduction in odds of clinical remission. For induction trials, prior biologic exposure was associated with lower odds of response and remission. Multicenter trials have lower placebo effects than single-center trials.

**Conclusions:**

These results enable future trials to incorporate design elements that reduce placebo rates as well as a precise benchmark for expected rates in clinical trials that do not include placebo.

## Graphical abstract

**Graphical Abstract:**
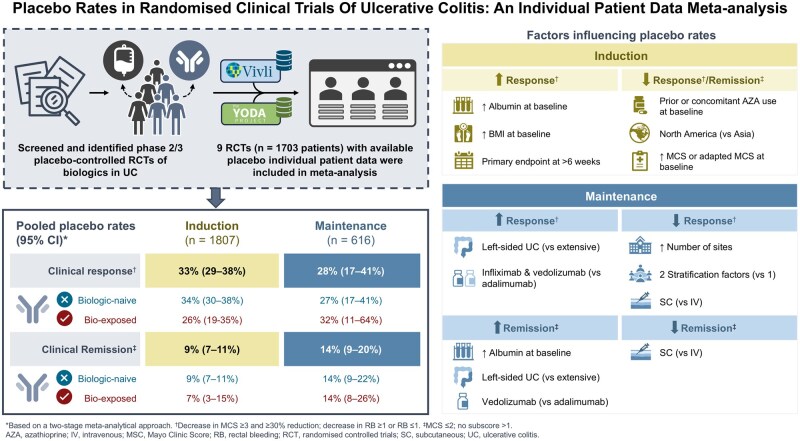

## 1. Introduction

Quantifying placebo rates in inflammatory bowel disease clinical trials and identifying the factors which influence these rates is important for the design, analysis, and interpretation of studies investigating novel therapeutic agents.^1–3^ The placebo response is a well-documented phenomenon affected by multiple factors including the impact of concomitant medications, natural variation in underlying disease, and regression toward the mean. In addition, placebo response is probably influenced by environmental and psychosocial factors.^4^^,^^5^

Prior studies have reported placebo rates in ulcerative colitis (UC) clinical trials based on meta-analysis of published studies.^1^^,^^2^^,^^6–8^ Although these studies identified some key trial- and disease-related characteristics associated with placebo response rates, a significant limitation to these analyses was the use of pooled data from published clinical trials. Access to individual patient data (IPD) from the placebo arms of UC trials may facilitate a more rigorous assessment of placebo rates and the factors influencing these rates.^9^ Furthermore, placebo rates in important subgroups of patients relevant to modern-day clinical trials can be assessed, such as rates in patients without exposure to biologics versus those with prior exposure. In addition, with a move towards designing clinical trials that reduce exposure to or eliminate placebo, such as open-label, head-to-head, or Bayesian designs, precise estimates of expected placebo rates are crucial to contextualize results. Thus, we aimed to estimate placebo rates across a range of endpoints in randomized controlled trials (RCTs) in UC and identify the patient- and trial-level factors influencing these rates using IPD.

## 2. Materials and methods

This systematic review and meta-analysis is reported according to the Preferred Reporting Items for Systematic Reviews and Meta-Analyses of Individual Patient Data (PRISMA-IPD) statement^10^ and was conducted following an a priori developed protocol (available upon request).

### 2.1. Study selection and data extraction

Search results previously reported in trial-level meta-analyses of placebo-controlled RCTs in UC were used to identify eligible trials.^2^^,^^6^^,^^7^ These meta-analyses used a consistent search strategy described in our study protocol. Two authors (VS and MH) independently and in duplicate identified placebo-controlled phase 2 or 3 RCTs of biologic therapy for adults with UC that used the Mayo Clinic Score (MCS) for enrolment and/or outcome assessment.^7^ Studies had a minimum duration of 2 weeks for induction trials, and 4 months for maintenance trials. Studies published prior to 2010 were excluded to ensure alignment with contemporary regulatory standards, comprehensive outcome assessments, and patient populations representative of current IBD practice.

From the studies identified, de-identified placebo arm IPD were requested for those trials that were available in Yale Open Data Access (YODA) (#2021-4829) and Vivli Inc. (#7288) data-sharing platforms at the time the protocol was developed (September 29, 2020). Patient- and trial-level placebo data were independently extracted by two authors (VS and MH). Participants who had received placebo at any timepoint were included if data were available at baseline and primary endpoint assessment.

### 2.2. Patient-level data collection

For induction and maintenance trials, eligible participants were permitted to have been initially assigned to placebo or to have been switched from active treatment to placebo. Baseline IPD on patient demographics, disease and clinical characteristics, concomitant medications, and previous exposure to biologics were compiled within the Vivli analytics platform. Demographic information included age, gender, body mass index (BMI), ethnicity, smoking status, disease duration, prior medical history related to inflammatory bowel disease (IBD) (eg, surgery, exposure to biologics, tumor necrosis factor [TNF] inhibitors, failure/intolerance to UC therapies), concomitant medication usage (eg, 5-aminosalicylates [5-ASAs], calcineurin inhibitors, immunomodulators, corticosteroids), and disease characteristics (eg, extent). We extracted data for disease activity measures, including the MCS and its individual subcomponents (eg, rectal bleeding and stool frequency score), Inflammatory Bowel Disease Questionnaire (IBDQ) score, and baseline concentration of albumin, C-reactive protein (CRP), fecal calprotectin (FCP), and fecal lactoferrin.

### 2.3. Trial-level data collection

Trial-level data collection included study design, setting, trial phase, drug type, route of administration, and blinding.

### 2.4. Outcomes

Primary outcomes were placebo clinical response and remission, as defined by the original studies. We assessed clinical response rates according to 2 definitions: (1) as defined by the included study; and (2) a decrease in adapted MCS by ≥2, a ≥35% reduction from baseline, and a ≥1-point decrease in rectal bleeding (RB) or absolute RB subscore ≤1. Two definitions of clinical remission were evaluated: (1) as defined by the included study; and (2) a Mayo endoscopic subscore (MES) of ≤1, a ≥1-point decrease in stool frequency (SF) to achieve an SF of ≤1, and RB = 0. Secondary outcomes included endoscopic response and remission, sustained clinical remission, sustained corticosteroid-free remission, adverse events (AEs), and serious adverse events (SAEs).

### 2.5. Statistical analysis

Demographic and clinical baseline information were analysed using summary statistics. Reported summary statistics for continuous variables include the mean, standard deviation (SD), median, and interquartile range (IQR), while frequency tables displaying the number and percentage of participants in each subgroup were reported for categorical variables. Descriptive statistics were used to report demographic information for UC trials, including age, endoscopy score at enrolment, gender, BMI, ethnicity, smoking status, disease duration (median, IQR), and the percentage of participants who had prior surgery for IBD, prior exposure to biologics, prior exposure to anti-TNFs, prior exposure to small molecule drugs, prior failure/intolerance to biologics, prior failure/intolerance to anti-TNFs, prior failure/intolerance to small molecule drugs, concomitant 5-ASA drugs, concomitant calcineurin inhibitors, concomitant immunomodulators, and concomitant corticosteroids. Disease extent (ulcerative proctitis, left-sided UC, or extensive UC) based upon the Montreal classification was also reported. Disease activity measures were reported for the baseline (Week 0) and primary endpoint assessment visits for both induction and maintenance periods.

Both one- and two-stage approaches for conducting an IPD meta-analyses were used to pool outcome data.^11^ In the one-stage approach, a generalized linear mixed model with binomial likelihood was used to model binary outcomes. A random (study-level) intercept was used to account for clustering within trials.^12^ The estimated mean intercept (and its associated 95% CI), which is equivalent to the overall proportion on the logit scale, was then back-transformed to the original proportion scale. The *lmer* function from the *lme4* package in R was used to fit these models.^13^

For the two-stage approach, a logistic regression model was used to obtain logit-transformed placebo rates and associated standard errors for each trial. A random-effects model was then applied to pool response and remission rates on the logit scale using the inverse-variance weighted average approach. Pooled estimates and associated 95% CIs were then converted back to the scale for proportions. A random‐effects model was chosen to provide inference about the average placebo rate in the population of studies from which the included studies are assumed to be a random selection. The *metafor* package in R was used for this approach.^14^ This paper reports on results obtained from employing the two-stage approach. Results derived from using the one-stage approach are included in the supplemental materials since results were identical or similar.

Subgroup analyses and meta-regression models were used to identify patient- and trial-level characteristics associated with placebo response and remission. For trial-level characteristics, stratum-specific placebo rates (and two-sided 95% CIs) were constructed by conducting a separate meta-analysis for each subgroup. Meta-regression models with trial‐level characteristics as predictors, and the log odds of the event rates as outcomes were used to assess the effect of study-level characteristics on placebo rates. For patient-level characteristics, the generalized linear mixed effects model described above was extended to include covariates. Continuous factors were centered, with adjusted proportions (and associated 95% CIs) obtained using the estimated intercept.

Estimates of τ^2^ (between-trial variance of the true placebo rates) were calculated to evaluate the extent to which placebo rates vary. Statistical significance of heterogeneity overall and within subgroups was assessed using Cochran’s Q test (*P* < .10 for the chi-squared test), and the magnitude was quantified using the I^2^ statistic,^15^ with a value greater than 50% representing substantial heterogeneity.^16^ Funnel plots were constructed to assess publication bias related to placebo rates by plotting the proportions on the logit scale against the standard error.

Both intention-to-treat (ITT) and as-observed (AO) populations were included in the conducted meta-analyses, ensuring a comprehensive assessment of the outcomes. Only the ITT population is reported throughout this paper since this reflects the primary methodology by which the original trials were assessed and provides a conservative estimate of effect size compared to the AO analysis.

A risk of bias assessment for included studies was independently conducted by two authors (VS and JKM) using the Cochrane risk of bias tool.^17^

## 3. Results

### 3.1. Study selection and characteristics

A total of 34 eligible studies were identified based on the previously conducted systematic reviews. Data from nine of these 34 eligible trials (26.5%) were available through YODA and Vivli at the time the protocol was submitted and included in the meta-analysis (Table 1; Figure S1).^18–26^

**Table 1.** Summary of included studies.

Study ID	Design	Setting	Intervention	Placebo patients (*n*)	Outcome timepoint (weeks)	Clinical response definition	Clinical remission definition
**NCT00488774 (PURSUIT-IV)**	Induction	Multicenter, multinational	Golimumab	77	6	Decrease in the MCS of ≥3 points and a ≥30% reduction from baseline; and a ≥1-point decrease in RB or an absolute RB subscore ≤1	MCS ≤2 with no subscore >1
**NCT00487539 (PURSUIT-SC)**	Induction	Multicenter, multinational	Golimumab	258	6	Decrease in the MCS of ≥3 points and a ≥30% reduction from baseline; and a ≥1-point decrease in RB or an absolute RB subscore ≤1	MCS ≤2 with no subscore >1
**NCT00783718 (GEMINI 1)**	Induction and maintenance	Multicenter, multinational	Vedolizumab	149 (induction) 126 (maintenance)	6 (induction) 52 (maintenance)	Decrease in the MCS of ≥3 points and a ≥30% reduction from baseline; and a ≥1-point decrease in RB or an absolute RB subscore ≤1	MCS ≤2 with no subscore >1
**NCT01863771 (PURSUIT-J)**	Maintenance	Multicenter, single country	Golimumab	31	54	Decrease in the MCS of ≥3 points and a ≥30% reduction from baseline; and a ≥1-point decrease in RB or an absolute RB subscore ≤1	MCS ≤2 with no subscore >1
**NCT00385736 (ULTRA 1)**	Induction	Multicenter, multinational	Adalimumab	130	8	Decrease in the MCS of ≥3 points and a ≥30% reduction from baseline; and a ≥1-point decrease in RB or an absolute RB subscore ≤1	MCS ≤2 with no subscore >1
**NCT00408629 (ULTRA 2)**	Induction and maintenance	Multicenter, multinational	Adalimumab	246 (induction) 246 (maintenance)	8 (induction) 52 (maintenance)	Decrease in the MCS of ≥3 points and a ≥30% reduction from baseline; and a ≥1-point decrease in RB or an absolute RB subscore ≤1	MCS ≤2 with no subscore >1
**NCT00853099**	Induction and maintenance	Multicenter, single country	Adalimumab	96 (induction) 96 (maintenance	8 (induction) 52 (maintenance)	Decrease in the MCS of ≥3 points and a ≥30% reduction from baseline; and a ≥1-point decrease in RB or an absolute RB subscore ≤1	MCS ≤2 with no subscore >1
**NCT02039505**	Induction and maintenance	Multicenter, single country	Vedolizumab	82 (induction) 68 (maintenance	10 (induction) 60 (maintenance)	Decrease in the MCS of ≥3 points and a ≥30% reduction from baseline; and a ≥1-point decrease in RB or an absolute RB subscore ≤1	MCS ≤2 with no subscore >1
**NCT01551290**	Induction and maintenance	Multicenter, single country	Infliximab	49 (induction) 49 (maintenance	8 (induction) 26 (maintenance)	Decrease in the MCS of ≥3 points and a ≥30% reduction from baseline; and a ≥1-point decrease in RB or an absolute RB subscore ≤1	MCS ≤2 with no subscore >1

Abbreviations: MCS, Mayo Clinic Score; RB, rectal bleeding.

Baseline demographic and clinical information for nine UC induction (*n* = 1087) and maintenance trials (*n* = 616) are summarized in Table 2. The overall characteristics of patients receiving placebo were similar across trials.

**Table 2.** Baseline characteristics of patients with ulcerative colitis assigned to receive placebo in induction and maintenance trials.

Characteristic	Induction (*N* = 1087)^a^	Missing values, *n*	Maintenance (*N* = 616)^a^	Missing values, *n*
**Age, years, mean (SD)**	40 (13.3)	0	41.1 (13.4)	0
**Body mass index, kg/m^2^, mean (SD)**	24.7 (5.4)	56	24.4 (5.4)	51
**Age at diagnosis, years, mean (SD)**	35 (13.3)	433	33 (13.6)	326
**Disease duration at baseline, years, mean (SD)**	4.6 (6.4)	433	8.1 (6.8)	326
**Sex, *n* (%)**		0		0
** Male**	653 (60.1)		374 (60.7)	
** Female**	434 (39.9)		242 (39.3)	
**Race, *n* (%)**		0		0
** White**	728 (67)		336 (54.5)	
** Other**	359 (33)		280 (45.5)	
**Prior surgery for IBD, *n* (%)**	12 (2.1)	507	29 (11.5)	363
**Smoking status, *n* (%)**		51		51
** Former smoker/never smoked**	972 (93.8)		514 (91)	
** Current smoker**	64 (6.2)		51 (9)	
**Disease location based on Montreal criteria at diagnosis, *n* (%)**		1424		204
** Left-sided UC**	90 (17.4)		82 (19.9)	
** Extensive UC**	274 (53)		219 (53.2)	
** Other**	153 (29.6)		111 (26.9)	
**Concomitant 5-ASA drugs at baseline, *n* (%)**	209 (27.8)	0	215 (34.9)	0
**Previous 5-ASA drug treatment, *n* (%)**	505 (48.7)	0	151 (28.2)	0
**Concomitant calcineurin inhibitor at baseline, *n* (%)**	8 (1.1)	0	5 (0.8)	0
**Previous calcineurin inhibitor treatment, *n* (%)**	10 (1.4)	0	8 (1.5)	31
**Concomitant 6-MP at baseline, *n* (%)**	20 (2.7)	0	31 (5)	0
**Previous 6-MP treatment, *n* (%)**	24 (3.4)	0	20 (3.7)	31
**Concomitant AZA at baseline, *n* (%)**	87 (11.6)	0	89 (14.4)	0
**Previous AZA treatment, *n* (%)**	85 (12.1)	0	76 (14.2)	31
**Concomitant MTX at baseline, *n* (%)**	10 (1.3)	0	7 (1.1)	0
**Previous MTX drug treatment, *n* (%)**	11 (1.6)	0	10 (1.9)	31
**Concomitant oral corticosteroid at baseline, *n* (%)**	233 (31)	0	228 (37)	0
**Previous oral corticosteroid treatment, *n* (%)**	433 (41.7)	0	123 (21.7)	0
**Prior exposure to anti-TNFs, *n* (%)**	114 (49.4)	384	72 (37.1)	80
**Prior failure of anti-TNFs, *n* (%)**	70 (30.3)	856	44 (22.7)	422
**Prior intolerance to anti-TNFs, *n* (%)**	14 (6.1)	856	14 (7.2)	422
**Previous exposure to biologic, *n* (%)**	114 (49.4)	0	72 (37.1)	0

a Percentages are based on the number of non-missing values for each category.

Abbreviations: 5-ASA, 5-aminosalicylic acid; 6-MP, 6-mercaptopurine; AZA, azathioprine; IBD, inflammatory bowel disease; MTX, methotrexate; SD, standard deviation; TNF, tumor necrosis factor; UC, ulcerative colitis.

Trial-level characteristics are summarized in Table S1. Three studies were induction-only, one was maintenance-only, and five included both phases. Most of the induction trials were multi-arm (6/8; 75%), quadruple-blind (4/8; 50%), phase 3 (6/8; 75%), multicenter, multinational (5/8; 62.5%) trials, investigating intravenous (IV) biologics (8/8; 100%). Most of the maintenance trials were phase 3 trials (6/6; 100%) conducted in Asia (4/6; 66.7%). These trials were equally distributed between two-arm (3/6; 50%) and three-arm (3/6; 50%) parallel designs, with mostly double blinding (3/6; 50%), followed by quadruple (2/6; 33.3%) and triple blinding (1/6; 16.7%). The majority of these maintenance trials were multicenter single-country trials (4/6; 66.7%), investigating both IV (3/6; 50%) and subcutaneous (SC) (3/6; 50%) biologics.

**Figure 1.**
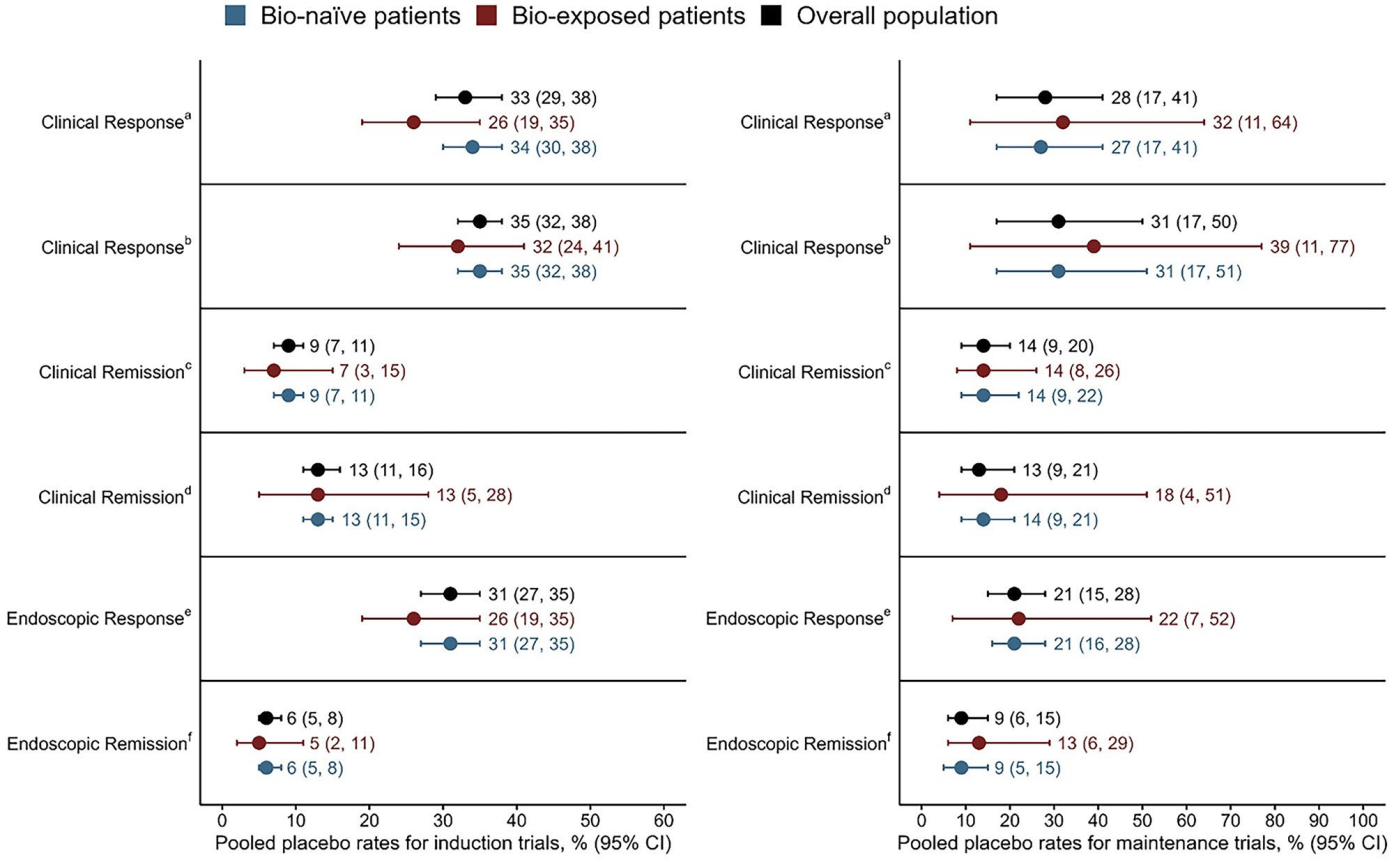
Pooled placebo clinical and endoscopic response and remission rates for induction and maintenance trials of ulcerative colitis using a 2-stage meta-analytical approach. Induction trials: *n* = 1087 (bio-exposed: *n *= 125); maintenance trials: *n* = 616 (bio-exposed: *n *= 81). Abbreviations: CI, confidence interval; MCS, Mayo Clinic Score; MES, Mayo Endoscopic Subscore; RB, rectal bleeding; SF, stool frequency. ^a^Decrease in MCS ≥3 and ≥30% reduction; decrease in RB ≥1 or RB ≤1. ^b^Decrease in adapted MCS ≥2 and ≥35% reduction; decrease in RB ≥1 or RB ≤1. ^c^MCS ≤2; no subscore >1. ^d^MES ≤1; decrease in SF ≥1; SF ≤1 and RB=0. ^e^MES ≤1. ^f^MES=0.

Pooled placebo clinical and endoscopic response and remission rates for induction and maintenance trials for the overall population and biologic-exposed and biologic-naïve subgroups are summarized in Figure 1. Results obtained from the two-stage approach are reported in the main text (see Table S2 for one-stage results).

### 3.2. Placebo clinical response

For induction trials, the pooled placebo clinical response rate using the trial definition was 33% (95% CI: 29%-38%, *I*^2^ = 48.9%; Table S2; Figure S2). In patients with prior biologic exposure, the pooled clinical response rate was 26% (95% CI 19%-35%) compared with 34% (95% CI: 30%-38%; Figure 1) in biologic-naïve patients. Using an alternative definition (a decrease in the adapted MCS ≥2, a ≥35% reduction from baseline, and a ≥1-point decrease in rectal bleeding or absolute rectal bleeding subscore ≤1), the pooled clinical response rate was 35% (95% CI: 32%-38%, *I*^2^ = 0%; Table S2; Figure S2).

For maintenance trials, the pooled placebo clinical response rate using the trial definition was 28% (95% CI: 17%-41%, *I*^2^ = 89.2%; Table S2; Figure S3). Biologic-naive patients showed a lower response rate (27%, 95% CI: 17%-41%) compared to biologic-exposed patients (32%, 95% CI: 11%-64%). Similarly, anti-TNF-naive patients had lower response rates (27%, 95% CI: 17%-41%) compared to anti-TNF-exposed patients (32%, 95% CI: 11%-64%). A similar response rate was found using the alternative definition (31%, 95% CI: 17%-50%, *I*^2^ = 93.7%; Figure S3). Again, biologic-naïve patients had lower rates of placebo clinical response (31%, 95% CI: 17%-51%) compared to biologic-exposed patients (39%, 95% CI: 11%-77%).

### 3.3. Placebo clinical remission

For induction trials, the pooled placebo remission rate using the trial definition was 9% (95% CI: 7%-11%, *I*^2^ = 0%; Table S2; Figure S4). Slightly lower placebo clinical remission rates were reported in biologic-exposed and TNF-exposed patients compared to biologic-naïve and TNF-naïve patients (7% [95% CI: 3%-15%) vs 9% [95% CI: 7%-11%] and 9% [95% CI: 7%-11%]). Using the alternative definition, the remission rate increased to 13% (95% CI: 11%-16%, *I*^2^ = 29.7%; Table S2; Figure S4).

For maintenance trials, the pooled placebo remission rate using the trial definition was 14% (95% CI: 9%-20%, *I*^2^ = 70.7%; Table S2; Figure S5). Using the alternative definition, the pooled placebo remission rate was 13% (95% CI: 9%-21%, *I*^2^ = 74.7%; Table S2; Figure S5). Biologic-naïve patients had similar rates of placebo clinical remission compared to biologic-exposed (14% [95% CI: 9%-22%] compared with 14% [95% CI: 8%-26%] using the clinical trial definition), regardless of the clinical remission definition.

### 3.4. Endoscopic response and remission, sustained clinical remission, corticosteroid-free remission, sustained corticosteroid-free remission, AEs, and SAEs

These outcomes were evaluated as secondary endpoints. In placebo-treated patients, the pooled endoscopic response and remission rates during induction were 31% and 6%, respectively, and 21% and 9% during maintenance. Sustained clinical remission and sustained corticosteroid-free remission were observed in 1%-3% of patients. Corticosteroid-free clinical remission occurred in 3%-5% of patients during induction and 8%-10% during maintenance. AEs were reported in 44% of placebo patients during induction and 79% during maintenance, while SAEs occurred in 10% and 12%-13%, respectively. Forest plots for endoscopic response and remission (Figures S6-S9), sustained clinical remission (Figures S10 and S11), corticosteroid-free remission (Figures S12-S15), sustained corticosteroid-free remission (Figures S16 and S17), AEs (Figures S18 and S19), and SAEs (Figures S20 and S21) are reported in the supplementary material.

### 3.5. Patient-level predictors of placebo response and remission

Of the 33 patient-level characteristics evaluated, only four had a statistically significant impact on the placebo response rate using the trial definition. These factors included BMI, albumin levels at baseline, azathioprine use at baseline, and previous azathioprine use (Table 3 and Table S3). A 1-unit (kg/m^2^) increase in BMI at baseline was associated with a 3% increase in the odds of clinical response (OR 1.03 95% CI 1.01-1.06; *P* = .008). A 10-unit (g/L) increase in albumin levels at baseline was associated with a 41% increase in the odds of clinical response (OR 1.41 95% CI 1.05-1.90; *P* = .023). Using the alternative definition for response, BMI and albumin level at baseline were significantly associated with placebo clinical response (OR 1.03, 95% CI: 1-1.05; *P* = .029 and OR 1.38, 95% CI: 1.04-1.82; *P* = .026, respectively; Table 3 and Table S4).

**Table 3.** Univariable regression analyses of select patient- and trial-level factors contributing to placebo clinical and endoscopic response and remission for induction trials in ulcerative colitis.

	Definition of clinical response, odds ratio (95% CI)		Definition of clinical remission, odds ratio (95% CI)		Definition of endoscopic response, odds ratio (95% CI)	Definition of endoscopic remission, odds ratio (95% CI)
	By trial definition	Decrease in adapted MCS ≥2 and ≥35% reduction; decrease in RB ≥1 or RB ≤1^a^	By trial definition	MES ≤1; decrease in SF ≥1; SF ≤1 and RB = 0^a^	MES ≤1^a^	MES = 0^a^
**Patient-level characteristics**						
**Age^b^**	1 (0.99, 1.01)	1 (0.99, 1.01)	1 (0.99, 1.02)	1 (0.99, 1.01)	1 (0.99, 1.01)	1 (0.98, 1.02)
**Age at diagnosis^b^**	1 (0.99, 1.01)	1 (0.99, 1.02)	0.99 (0.97, 1.02)	0.99 (0.97, 1.01)	0.99 (0.98, 1)	1 (0.97, 1.03)
**Sex (male vs female)**	0.95 (0.73, 1.23)	0.97 (0.75, 1.25)	0.73 (0.48, 1.12)	0.86 (0.6, 1.24)	0.67 (0.51, 0.87)	0.96 (0.57, 1.62)
**BMI^b^**	1.03 (1.01, 1.06)	1.03 (1, 1.05)	1.01 (0.98, 1.06)	1.03 (0.99, 1.06)	1.03 (1.01, 1.06)	1.04 (1, 1.09)
**CRP^c ^**	1.03 (0.91, 1.15)	1.06 (0.95, 1.19)	0.83 (0.63, 1.1)	0.84 (0.67, 1.05)	0.92 (0.8, 1.05)	0.73 (0.46, 1.14)
**Albumin^c ^**	1.41 (1.05, 1.9)	1.38 (1.04, 1.82)	1.29 (0.8, 2.1)	1.21 (0.8, 1.84)	1.8 (1.31, 2.48)*	1.87 (0.97, 3.61)
**MCS score^b^**	0.98 (0.91, 1.06)	0.93 (0.86, 1)	0.74 (0.64, 0.85)*	0.85 (0.76, 0.95)	0.72 (0.66, 0.79)*	0.7 (0.59, 0.83)*
**Adapted MCS scoreb**	0.96 (0.88, 1.05)	0.92 (0.84, 1)	0.73 (0.63, 0.85)*	0.83 (0.73, 0.95)	0.69 (0.63, 0.77)*	0.67 (0.56, 0.81)*
**IBDQ score^c^**	0.98 (0.94, 1.02)	1 (0.96, 1.03)	1.07 (1, 1.14)	1.05 (1, 1.11)	1.02 (0.98, 1.07)	0.99 (0.91, 1.09)
**Disease extent (left-sided UC vs other)**	0.61 (0.35, 1.05)	0.74 (0.44, 1.24)	1.59 (0.64, 3.91)	0.95 (0.46, 1.97)	0.92 (0.52, 1.61)	1.32 (0.37, 4.69)
**Prior exposure to biologic therapy^d^**	0.72 (0.45, 1.15)	0.83 (0.56, 1.24)	0.7 (0.33, 1.47)	0.97 (0.53, 1.76)	0.82 (0.51, 1.32)	0.89 (0.36, 2.18)
**Prior exposure to anti-TNF therapy^d^ ^,^ ^e^**	0.72 (0.45, 1.15)	0.79 (0.49, 1.3)	0.45 (0.16, 1.26)	0.97 (0.53, 1.76)	0.82 (0.51, 1.32)	0.89 (0.36, 2.18)
**Prior failure of anti-TNF therapy^d^ ^,^ ^f^**	0.98 (0.55, 1.72)	1.43 (0.79, 2.59)	0.74 (0.23, 2.36)	1.32 (0.67, 2.58)	0.86 (0.48, 1.53)	0.71 (0.21, 2.34)
**Concomitant oral corticosteroids^d^**	0.97 (0.71, 1.34)	0.99 (0.71, 1.37)	1.26 (0.74, 2.14)	1.37 (0.81, 2.29)	1.39 (0.85, 2.25)	1.2 (0.65, 2.2)
**Trial-level characteristics**						
**Multicenter, single-country vs multicenter, multinational**	1.09 (0.71, 1.68)	0.97 (0.71, 1.32)	1.47 (0.91, 2.37)	1.45 (0.96, 2.2)	0.84 (0.54, 1.29)	1.56 (0.74, 3.3)
**Number of study sites^c ^**	0.98 (0.95, 1.01)	0.99 (0.97, 1.02)	0.97 (0.93, 1.01)	0.98 (0.94, 1.01)	1 (0.96, 1.04)	0.92 (0.85, 1.01)
**Number of follow-up visits^b^**	1.00 (0.97, 1.03)	0.99 (0.97, 1.01)	1.01 (0.98, 1.05)	1.01 (0.97, 1.04)	1 (0.97, 1.03)	0.97 (0.91, 1.03)

Green, significantly increased odds of achieving the placebo outcome (*P* < .05); orange, significantly decreased odds of achieving the placebo outcome (*P* < .05); grey, not statistically significant (*P *> .05); *P ≤ .001.

a *n* = 1087 (bio-exposed: *n = *125).

b Evaluated per 1-unit increase.

c Evaluated per 10-unit increase.

d Evaluated with the comparison of ‘yes vs no’.

e *n *= 703 (prior anti-TNF exposure: *n *= 87).

f *n* = 251 (prior anti-TNF failure: *n = *70).

BMI, body mass index; CI, confidence interval; CRP, C-reactive protein; IBDQ, Inflammatory Bowel Disease Questionnaire; MCS, Mayo Clinic Score; MES, Mayo Endoscopic Subscore; RB, rectal bleeding; SF, stool frequency; TNF, tumor necrosis factor; UC, ulcerative colitis.

### 3.6. Trial-level predictors of placebo response and remission

Among 12 trial-level characteristics evaluated, time of primary endpoint was the only statistically significant factor impacting the placebo response rate (using the trial definition). Endpoints at greater than 6 weeks were associated with a 42% increase in the odds of clinical response (OR 1.42, 95% CI: 1.06-1.90, *P* = .018; Table 3 and Table S5). Using the alternative definition, no statistically significant factors were identified (Table 3 and Table S6).

Using the trial definition, only two patient-level factors had a statistically significant impact on the placebo remission rate. A 1-point increase in the MCS and adapted MCS was associated with a 26% (OR 0.74, 95% CI: 0.64-0.85; *P* < .001) and 27% (OR 0.73, 95% CI: 0.63-0.85; *P* < .001) reduction in the odds of clinical remission, respectively (Table 3 and Table S7). Using the alternative definition of remission, two factors were associated with a significant reduction in placebo remission. One-point increases in the MCS and adapted MCS from baseline were associated with 15% (OR = 0.85, 95% CI: 0.76-0.95; *P* = .005) and 17% (OR = 0.83, 95% CI: 0.73-0.95; *P* = .005) reductions in the odds of clinical remission, respectively (Table 3 and Table S8).

Using the trial definitions for remission, the only statistically significant trial-level characteristic was location. North America was associated with a 56% decrease in the odds of clinical remission (OR 0.44, 95% CI: 0.19-1; *P* = .049) when Asia was the reference (Table 3 and Table S9). A similar association was observed for the alternative definition of remission (Table 3 and Table S10).

Patients with left-sided UC had a 90% increase in the odds of response compared to extensive UC (OR 1.9, 95% CI: 1.02-3.54; *P* = .043; Table 4 and Table S11). Statistically significant associations were not found for the alternative definition of response (Table 4 and Table S12).

**Table 4.** Univariable regression analyses of select patient- and trial-level factors contributing to placebo clinical and endoscopic response and remission for maintenance trials in ulcerative colitis.

	Definition of clinical response, odds ratio (95% CI)		Definition of clinical remission, odds ratio (95% CI)		Definition of endoscopic response, odds ratio (95% CI)	Definition of endoscopic remission, odds ratio (95% CI)
	By trial definition	Decrease in adapted MCS ≥2 and ≥35% reduction; decrease in RB ≥1 or RB ≤1^a^	By trial definition	MES ≤1; decrease in SF ≥1; SF ≤1 and RB = 0^a^	MES ≤1^a^	MES = 0^a^
**Patient-level characteristics**						
**Age^b^**	1.01 (0.99, 1.02)	1.01 (1, 1.02)	1 (0.98, 1.02)	1 (0.98, 1.01)	1 (0.98, 1.01)	1.01 (0.99, 1.03)
**Age at diagnosis^b^**	1 (0.98, 1.02)	1 (0.98, 1.02)	1.01 (0.98, 1.03)	1 (0.98, 1.02)	1 (0.98, 1.02)	1.01 (0.98, 1.04)
**Sex (male vs female)**	0.85 (0.58, 1.25)	0.91 (0.62, 1.35)	0.96 (0.59, 1.57)	1.09 (0.66, 1.78)	0.81 (0.54, 1.22)	0.92 (0.52, 1.62)
**BMI^b^**	1.01 (0.97, 1.05)	1.01 (0.97, 1.05)	1 (0.96, 1.05)	1 (0.95, 1.05)	1.02 (0.98, 1.06)	1.03 (0.98, 1.09)
**CRP^c^**	0.98 (0.8, 1.18)	0.93 (0.77, 1.13)	1.02 (0.82, 1.27)	1.03 (0.84, 1.28)	0.93 (0.74, 1.17)	1.01 (0.78, 1.3)
**Albumin^c^**	1.5 (0.97, 2.3)	1.48 (0.96, 2.28)	1.92 (1.09, 3.39)	2.33 (1.29, 4.19)	2.39 (1.47, 3.88)*	2.33 (1.18, 4.6)
**MCS score^b^**	1.01 (0.9, 1.13)	1 (0.89, 1.13)	0.94 (0.81, 1.09)	0.98 (0.85, 1.14)	0.91 (0.8, 1.03)	0.99 (0.83, 1.17)
**Adapted MCS score^b^**	0.99 (0.87, 1.13)	1 (0.87, 1.14)	0.93 (0.78, 1.09)	0.95 (0.81, 1.12)	0.88 (0.76, 1.01)	0.96 (0.79, 1.16)
**IBDQ score^c^**	1.05 (0.98, 1.12)	1.04 (0.97, 1.12)	1.05 (0.95, 1.14)	1.06 (0.96, 1.16)	1.06 (0.99, 1.14)	1.03 (0.93, 1.14)
**Disease extent (left-sided UC vs other)**	1.9 (1.02, 3.54)	1.54 (0.82, 2.89)	2.85 (1.5, 5.41) *	2.21 (1.1, 4.45)	2.16 (1.2, 3.88)	3.89 (1.69, 8.95)*
**Prior exposure to biologic therapy^d^,**	1.05 (0.56, 1.99)	0.88 (0.46, 1.67)	0.65 (0.29, 1.46)	0.89 (0.41, 1.94)	0.85 (0.42, 1.69)	1.17 (0.48, 2.84)
**Prior exposure to anti-TNF therapy^d^ ^,^ ^e^**	1.05 (0.56, 1.99)	0.88 (0.46, 1.67)	0.65 (0.29, 1.46)	0.89 (0.41, 1.94)	0.85 (0.42, 1.69)	1.17 (0.48, 2.84)
**Prior failure of anti-TNF therapy^d^ ^,^ ^f^**	0.89 (0.42, 1.91)	0.58 (0.26, 1.29)	0.39 (0.14, 1.12)	0.8 (0.32, 2.04)	0.73 (0.31, 1.72)	0.75 (0.24, 2.28)
**Concomitant oral corticosteroids^d^**	0.63 (0.38, 1.02)	0.66 (0.4, 1.08)	0.7 (0.39, 1.25)	0.71 (0.4, 1.26)	0.77 (0.46, 1.29)	0.47 (0.23, 0.94)
**Trial-level characteristics**						
**Multicenter, single-country vs multicenter, multinational**	1.83 (0.51, 6.57)	2.34 (0.44, 12.3)	1.36 (0.49, 3.77)	1.47 (0.5, 4.34)	1.56 (0.74, 3.3)	1.37 (0.42, 4.48)
**Number of study sites^c^**	0.87 (0.77, 0.99)	0.86 (0.71, 1.05)	0.96 (0.83, 1.1)	0.97 (0.83, 1.13)	0.92 (0.85, 1.01)	0.98 (0.82, 1.17)
**Number of follow-up visits^b^**	0.93 (0.85, 1.01)	0.92 (0.82, 1.04)	0.99 (0.91, 1.07)	0.99 (0.91, 1.09)	0.97 (0.91, 1.03)	0.99 (0.89, 1.1)

Green, significantly increased odds of achieving the placebo outcome (*P* < .05); orange, significantly decreased odds of achieving the placebo outcome (*P* < .05); grey, not statistically significant (*P* > .05); * *P* ≤ .001.

a *n* = 616 (bio-exposed: *n *= 81).

b Evaluated per 1-unit increase.

c Evaluated per 10-unit increase.

d Evaluated with the comparison of ‘yes vs no’.

e *n *= 536 (prior anti-TNF exposure: *n *= 59).

f *n* = 194 (prior anti-TNF failure: *n = *44).

BMI, body mass index; CI, confidence interval; CRP, C-reactive protein; IBDQ, Inflammatory Bowel Disease Questionnaire; MCS, Mayo Clinic Score; MES, Mayo Endoscopic Subscore; NA, lack of model convergence due to sparse data; RB, rectal bleeding; SF, stool frequency; TNF, tumor necrosis factor; UC, ulcerative colitis.

The number of centers had a statistically significant impact on the response rate (based on the trial definition). A 10-center increase was associated with a 13% decrease in the odds of response (OR 0.87, 95% CI: 0.77-0.99, *P* = .032; Table 4 and Table S13). The use of infliximab (OR 6.69, 95% CI 2.78-16.08; *P* < .001, trial and alternative definition; Tables S13 and S14) and vedolizumab (OR 1.97, 95% CI 1.03-3.79; *P* = .041; trial definition) were associated with an increase in placebo clinical response compared to adalimumab. Two stratification factors were associated with a significant reduction in the odds of response (OR 0.28, 95% CI: 0.14-0.56; *P* < .001; trial and alternative definition) compared to a single stratification factor. Additionally, the SC route of administration was a significant factor in reducing the odds of placebo response compared to IV (OR 0.35, 95% CI: 0.14-0.87; *P* = .023; Table 4 and Table S13).

Using the trial (OR 1.92, 95% CI: 1.09-3.39; *P* = .024; Table 4 and Table S15) and alternative definitions (OR 2.33, 95% CI: 1.29-4.19; *P* = .005; Table 4 and Table S16), a 10-unit (g/L) increase in albumin at baseline was associated with an increase in the odds of placebo remission. Using the trial (OR 2.85, 95% CI: 1.5-5.41; *P* = .001; Table 4 and Table S15) and alternative definitions (OR 2.21, 95% CI: 1.1-4.45; *P* = .026; Table 4 and Table S16), left-sided colitis was associated with a higher placebo remission rate.

Table 4 and Tables S17 and S18 summarize the univariable regression analyses of trial-level factors contributing to remission in maintenance trials. Using the trial definition, vedolizumab (OR 2.9, 95% CI: 1.42-5.90; *P* = .003) with adalimumab as the reference was associated with an increased odds of remission and SC route of administration (OR 0.44, 95% CI: 0.23-0.83; *P* = .011) was associated with a reduced odds of remission compared to IV.

### 3.7. Patient- and trial-level predictors of placebo secondary outcomes

Factors associated with endoscopic response and remission (Tables S19–S26), sustained clinical remission (Tables S27–S30), corticosteroid-free clinical remission (Tables S31–S38), sustained corticosteroid-free clinical remission (Tables S39–S42), AEs (Tables S43–S46), and SAEs (Tables S47–S50) among patients receiving placebo are summarized in the supplementary material. Briefly, higher baseline albumin level and lower baseline disease activity better predicted endoscopic response and/or remission and clinical outcomes (including sustained clinical and/or corticosteroid-free remission). Corticosteroid use was associated with a lower chance of achieving clinical outcomes. Additional patient-level associations with endoscopic or clinical outcomes included left-sided UC and disease duration. Trial-level features that better predicted endoscopic and/or clinical outcomes included vedolizumab use and quadruple or triple blinding. For safety, lower albumin level and higher disease activity were associated with an increased chance of AEs and SAEs.

### 3.8. Risk of bias assessment

The overall risk of bias among the included studies was predominantly low, with only a few domains rated as unclear (Table S51).

## 4. Discussion

In this IPD meta-analysis, the pooled placebo clinical response rate for induction trials was 33%, and for maintenance trials, it ranged from 27% to 31%. For clinical remission, placebo rates ranged from 9% to 13% during induction and from 13% to 14% during maintenance. Remission rates were consistent regardless of prior biologic exposure status.

Through access to patient-level data, we found some notable patient characteristics that influenced placebo rates. First, better nutritional status and lower biochemical burden were associated with an increased response to placebo, as demonstrated by a 3% increase in the odds of clinical response during induction trials for each 1-unit increase in BMI and a 41% increase in the odds of clinical response for every 10-unit increase in albumin.

Placebo responses have been shown to be significantly influenced by metabolic factors in RCTs, particularly in conditions such as hyperlipidemia and non-alcoholic fatty liver disease.^27^^,^^28^ Notably, a recent meta-analysis including 20 454 patients with a mean BMI of 35.66 kg/m^2^ (95% CI: 34.9-36.2) highlighted significant placebo effects in obesity pharmacological RCTs, emphasizing the impact of BMI and nutritional status on placebo outcomes.^29^ Second, greater baseline disease activity as captured by the MCS or adapted MCS was consistently associated with lower placebo remission rates, which supports our findings on endoscopic outcomes, as well as prior meta-analyses showing reduced placebo responses in patients with more severe endoscopic inflammation.^1^^,^^7^^,^^8^ Third, disease distribution influenced placebo rates since those with left-sided UC had a notable increase in their odds of placebo clinical response (OR 1.9, 95% CI: 1.02-3.54; *P* = .043) and remission (OR 2.21, 95% CI: 1.1-4.45; *P* = .026) compared to more extensive disease; this is likely to be a reflection of lower inflammatory burden in patients with limited disease, supported by recent intriguing observations that suggested differential efficacy of medical therapies in UC according to disease location.^30^ Collectively, the identified factors suggest that more severe disease activity as well as more extensive disease are associated with lower placebo rates, underscoring the importance of confirming high inflammatory burden at enrolment in clinical trials of moderate to severe UC, in order to minimize placebo rates and maximize efficiency within clinical trials.

Aside from patient characteristics, there were several factors related to clinical trial design that were found to influence placebo rates. First, multicenter trials tend to show lower treatment effects compared to single-center trials.^31^ We observed a similar pattern for placebo response. As single-center trials often show larger treatment effects due to homogeneous populations, potential patient selection bias, investigator expertise, stringent protocol adherence, increased participant attention, and publication bias favoring positive results,^32^ a higher risk of bias in single-center trials may extend to placebo rates. Second, trial location influenced placebo rates with a decreased odds of placebo clinical remission in North America compared to Asia (OR 0.44, 95% CI 0.19-1; *P* = .049); these findings could be related to differences in care processes or pathways (eg, a higher proportion of patients exposed to biologics [49% in North America vs 12% in Asia] or use of concomitant baseline steroids [41% vs 18%]). Third, trial duration influenced placebo rates, with endpoints assessed beyond 6 weeks associated with a 42% increase in the odds of clinical response. Similarly, longer disease duration was associated with a higher odds of achieving corticosteroid-free clinical remission under placebo. These findings are probably related to the natural disease course but also to prolonged participant engagement or evolving psychological factors over an extended period, contributing to a more pronounced placebo response. Fourth, route of administration revealed varying response rates. The SC route of administration was a significant factor in reducing the odds of placebo response and remission compared to IV administration. This could be related to expectation bias whereby the IV route of administration could be perceived as a more effective or faster acting intervention than SC administration. Finally, we observed new findings regarding the impact of prior biologic exposure on placebo response rates. Placebo remission rates were similar regardless of biologic exposure status. However, in the induction phases, patients with prior biologic exposure showed slightly lower placebo response rates compared to those without prior exposure. In contrast, during maintenance trials, biologic-naive patients exhibited lower placebo response rates than biologic-exposed patients. This difference may be explained by the fact that response is a less objective criterion than remission and is more prone to expectation bias.

Despite the availability of multiple effective therapies, placebo-controlled designs remain common, raising questions about equipoise and patient acceptability.^33^^,^^34^ Current regulatory frameworks continue to endorse placebo, but both the FDA and EMA stress the importance of minimizing unnecessary exposure by limiting placebo duration, defining escape criteria, and considering active comparator designs where feasible. Recent expert consensus similarly highlights the need for harmonization between agencies to reduce redundant placebo-controlled trials and to promote alternatives, including adaptive or active comparator strategies, particularly in populations with advanced or refractory disease.^35^

Several strategies have been proposed to reduce placebo exposure including asymmetrical randomization, inclusion of an active comparator arm, and early transition to open-label therapy after induction in placebo non-responders. Alternative clinical trial methods including Bayesian designs can reduce placebo exposure by using historical data, particularly at earlier phases of trial design for which there is well-established regulatory guidance.^36^^,^^37^ Our results provide novel and precise data to help inform Bayesian priors.

This is the first IPD meta-analysis based on a systematic review that assessed placebo response and associated patient- and trial-level predictors of response in patients with UC. The strengths of our study include the use of patient-level data allowing estimates of placebo rates in subpopulations as well as factors influencing these rates, robust IPD from nine RCTs, a large sample size, and diverse participants. Unlike meta-analyses relying on summary statistics,^1^^,^^7^^,^^38^ the use of IPD offers advantages such as consistent application of inclusion/exclusion criteria, the ability to address between-study heterogeneity, and minimization of selective reporting bias.^9–11^ However, there were some limitations to our meta-analysis that are worth noting. The analysis is constrained by the internal and external validity of the included trials, and only nine eligible studies provided IPD for inclusion, raising the possibility that negative trials may be underrepresented. Furthermore, our findings may not be generalizable to RCTs of ustekinumab or oral small molecule drugs, which were not available at the time of protocol submission. Our findings may also not be generalizable to RCTs of Crohn’s disease, in which placebo responses may differ, as we have recently shown in a paired IPD meta-analysis.^39^ In addition, a potential carry-over effect was present in only 1 trial, where placebo patients in the maintenance phase had previously received vedolizumab, but this involved a limited number of patients and is unlikely to have materially influenced placebo response rates. Finally, heterogeneity remained unexplained even after conducting meta-regression analyses. The multifactorial nature of the placebo response makes identifying factors that could explain this heterogeneity challenging, and factors not captured in our dataset, such as psychological state and cultural context, may also play a role and further confound the results.

This IPD meta-analysis significantly broadens our understanding, surpassing the insights gained from individual trials and previous aggregated data meta-analyses. Baseline disease severity, prior medications, and nutritional status influenced placebo response rates, emphasizing the significance of individual patient profiles in forecasting treatment outcomes. These contemporary results will enable future trials that include placebo to incorporate design elements that enable reduction of placebo rates. These results also provide a precise benchmark for expected rates in clinical trials that do not include placebo, such as open-label designs, head-to-head trials, and those incorporating external control arms or Bayesian designs.

## Supplementary Material

jjaf191_Supplementary_Data

## Data Availability

The data that support the findings of this study are available from Vivli Inc. and the Yale University Open Data Access Project. Restrictions apply to the availability of these data, which were used under licence for this study. The research proposal is publicly available on the Vivli platform (https://search.vivli.org/doiLanding/dataRequests/PR00007288). Interested parties may contact the corresponding author (vjairath@uwo.ca) to request access to the study protocol and statistical analysis plan.
