# Correction to: Placebo rates in randomized clinical trials of ulcerative colitis: an individual patient data meta-analysis

**DOI:** 10.1093/ecco-jcc/jjag070

**Published:** 2026-06-03

This is a correction to: Virginia Solitano, Malcolm Hogan, Siddharth Singh, Silvio Danese, Laurent Peyrin-Biroulet, Sudheer Vuyyuru, John K Macdonald, Guangyong Zou, Yuhong Yuan, Bruce E Sands, Remo Panaccione, Brian G Feagan, Jurij Hanzel, Rocio Sedano, Parambir Dulai, Neeraj Narula, Christopher Ma, Vipul Jairath, Placebo rates in randomized clinical trials of ulcerative colitis: an individual patient data meta-analysis, *Journal of Crohn’s and Colitis*, Volume 19, Issue 10, October 2025, jjaf191, https://doi.org/10.1093/ecco-jcc/jjaf191

The thirteenth author’s name has been corrected to read “Jurij Hanzel”. 

