# Supplementary material for: Placebo rates in randomized clinical trials of ulcerative colitis: an individual patient data meta-analysis
Source: J Crohns Colitis. 2025 Nov 9;19(10):jjaf191. doi: 10.1093/ecco-jcc/jjaf191 (PMC12668678; doi:10.1093/ecco-jcc/jjaf191)
Supplement: jjaf191_Supplementary_Data [file jjaf191_supplementary_data.zip › RCT-01374-SUPPLEMENT-29OCT2025-clean.docx]

**SUPPLEMENTARY MATERIAL**

#

# **Table S1.** Characteristics of included studies.

| **Characteristic** | **Induction trials (N=8) ^a^** | **Maintenance trials (N=6) ^a^** |
| --- | --- | --- |
| Number of centres, mean (SD) | 92.9 (57.8) | 63.7 (38.9) |
| Number of follow-up visits, mean (SD) | 12 (7.6) | 15.8 (6.9) |
| Trial start year, n (%) |  |  |
| 2010 and before | 7 (87.5) | 4 (66.7) |
| After 2010 | 1 (12.5) | 2 (33.3) |
| Trial end year, n (%) |  |  |
| 2010 and before | 4 (50) | 1 (16.7) |
| After 2010 | 4 (50) | 5 (83.3) |
| Trial design, n (%) |  |  |
| 2-arm parallel | 2 (25) | 3 (50) |
|  |  |  |
| Multi-arm parallel | 6 (75) | 3 (50) |
| Drug, n (%) |  |  |
| Adalimumab | 3 (37.5) | 2 (33.3) |
| Golimumab | 2 (25) | 1 (16.7) |
| Infliximab | 1 (12.5) | 1 (16.7) |
| Vedolizumab | 2 (25) | 2 (33.3) |
| Number of arms, n (%) |  |  |
| 2 | 2 (25) | 3 (50) |
| 3 | 4 (50) | 3 (50) |
| 4 | 2 (25) | 0 (0) |
| Trial phase, n (%) |  |  |
| 2/3 | 2 (25) | 0 (0) |
| 3 | 6 (75) | 6 (100) |
| Location, n (%) |  |  |
| Asia | 3 (37.5) | 4 (66.7) |
| Multi-continental | 4 (50) | 1 (16.7) |
| North America | 1 (12.5) | 1 (16.7) |
| Trial setting, n (%) |  |  |
| Multicentre, multinational | 5 (62.5) | 2 (33.3) |
| Multicentre, single-country | 3 (37.5) | 4 (66.7) |
| Route of administration, n (%) |  |  |
| IV | 4 (50) | 3 (50) |
|  |  |  |
| SC | 4 (50) | 3 (50) |
| Blinding, n (%) |  |  |
| Double | 3 (37.5) | 3 (50) |
| Quadruple | 4 (50) | 2 (33.3) |
| Triple | 1 (12.5) | 1 (16.7) |
| Number of screening visits, n (%) |  |  |
| 1 | 8 (100) | 6 (100) |
|  |  |  |
|  |  |  |

^a^ Percentages are based on the number of non-missing values for each category.

Abbreviations: IV, intravenous; SC subcutaneous.

# **Table S2.** Overall pooled placebo outcome rates for induction and maintenance trials in ulcerative colitis using one-stage and two-stage meta-analytical approaches.

| **Outcome** | **Period** | **Definition** | **Trials (n)** | **Patients (n)** | **Proportion (95% CI) One-stage** | **Proportion (95% CI) Two-stage** | **I^2^ (%)** | **Het. p-value ^a^** |
| --- | --- | --- | --- | --- | --- | --- | --- | --- |
| Clinical response | Induction | Trial definition | 8 | 1087 | 33 (30, 37) | 33 (29, 38) | 48.9 | 0.069 |
|  |  | Decrease in Adapted MCS ≥2 and ≥35% reduction; decrease in RB ≥1 or RB ≤1 | 8 | 1087 | 35 (32, 38) | 35 (32, 38) | 0 | 0.453 |
|  |  |  |  |  |  |  |  |  |
|  |  |  |  |  |  |  |  |  |
|  |  |  |  |  |  |  |  |  |
|  | Maintenance | Trial definition | 6 | 616 | 27 (18, 40) | 28 (17, 41) | 89.2 | <0.001 |
|  |  | Decrease in Adapted MCS ≥2 and ≥35% reduction; decrease in RB ≥1 or RB ≤1 | 6 | 616 | 31 (18, 48) | 31 (17, 50) | 93.7 | <0.001 |
|  |  |  |  |  |  |  |  |  |
|  |  |  |  |  |  |  |  |  |
|  |  |  |  |  |  |  |  |  |
| Clinical remission | Induction | Trial definition | 8 | 1087 | 9 (7, 10) | 9 (7, 11) | 0 | 0.52 |
|  |  | MES ≤1; decrease in SF ≥1; SF ≤1 and RB=0 | 8 | 1087 | 13 (11, 15) | 13 (11, 16) | 29.7 | 0.211 |
|  |  |  |  |  |  |  |  |  |
|  | Maintenance | Trial definition | 6 | 616 | 13 (9, 19) | 14 (9, 20) | 70.7 | 0.003 |
|  |  | MES ≤1; decrease in SF ≥1; SF ≤1 and RB=0 | 6 | 616 | 13 (9, 20) | 13 (9, 21) | 74.7 | <0.001 |
|  |  |  |  |  |  |  |  |  |
| Endoscopic response | Induction | MES ≤ 1 | 8 | 1087 | 31 (27, 34) | 31 (27, 35) | 45 | 0.078 |
|  | Maintenance | MES ≤ 1 | 6 | 616 | 21 (15, 27) | 21 (15, 28) | 70 | 0.005 |
|  |  |  |  |  |  |  |  |  |
| Endoscopic remission | Induction | MES = 0 | 8 | 1087 | 6 (5, 8) | 6 (5, 8 | 14.3 | 0.387 |
|  |  |  |  |  |  |  |  |  |
|  |  |  |  |  |  |  |  |  |
|  | Maintenance | MES = 0 | 6 | 616 | 9 (6, 14) | 9 (6, 15) | 69 | 0.005 |
|  |  |  |  |  |  |  |  |  |
|  |  |  |  |  |  |  |  |  |
| Sustained clinical  remission | N/A | Trial definition | 6 | 616 | 1 (0, 11) | 3 (1, 10) | 77.6 | 0.003 |
|  |  | MES ≤1; decrease in SF ≥1; SF ≤1 and RB=0 | 6 | 616 | 1 (0, 7) | 3 (2, 7) | 36.8 | 0.124 |
| Corticosteroid-free  clinical remission | Induction | Trial definition | 8 | 1087 | 3 (1, 6) | 4 (2, 8) | 78.1 | 0.002 |
|  |  | MES ≤1; decrease in SF ≥1; SF ≤1 and RB=0 | 8 | 1087 | 4 (2, 10) | 5 (3, 11) | 87 | <0.001 |
|  | Maintenance | Trial definition | 6 | 616 | 8 (4, 16) | 10 (6, 18) | 75.4 | 0.005 |
|  |  | MES ≤1; decrease in SF ≥1; SF ≤1 and RB=0 | 6 | 616 | 9 (4, 16) | 10 (6, 17) | 73.1 | 0.004 |
| Sustained corticosteroid-free clinical remission | N/A | Trial definition | 6 | 616 | 1 (0, 10) | 3 (1, 9) | 75 | 0.006 |
|  |  | MES ≤1; decrease in SF ≥1; SF ≤1 and RB=0 | 6 | 616 | 1 (0, 9) | 3 (1, 9) | 67.1 | 0.03 |
|  |  |  |  |  |  |  |  |  |
| Adverse events | Induction | N/A | 7 | 991 | 44 (35, 53) | 44 (35, 54) | 88.6 | <0.001 |
|  | Maintenance | N/A | 5 | 520 | 79 (58, 91) | 78 (55, 91) | 94.9 | <0.001 |
| Serious adverse events | Induction | N/A | 7 | 991 | 10 (5, 19) | 10 (5, 20) | 92.7 | <0.001 |
|  | Maintenance | N/A | 5 | 520 | 12 (10, 15) | 13 (10, 16) | 0 | 0.339 |

^a^ Measures of heterogeneity are based on the two-stage approach.

Abbreviations: CI, confidence interval; MCS Mayo Clinic Score; RB, rectal bleeding; MES, Mayo endoscopic subscore; SF, stool frequency.

# **Table S3.** Univariable regression analyses of patient-level factors contributing to clinical response (trial definition) for induction trials in ulcerative colitis.

| **Patient Characteristic** | **Odds Ratio (95% CI)** | **p-value** |
| --- | --- | --- |
| Age | 1 (0.99, 1.01) | 0.889 |
| Body mass index | 1.03 (1.01, 1.06) | 0.008 |
| Age at diagnosis | 1 (0.99, 1.01) | 0.995 |
| Disease duration at baseline | 1.01 (0.98, 1.03) | 0.699 |
| Albumin level at baseline (per 10-unit increase) | 1.41 (1.05, 1.9) | 0.023 |
| CRP level at baseline (per 10-unit increase) | 1.03 (0.91, 1.15) | 0.672 |
| FCP level at baseline (per 10-unit increase) | 1 (1, 1) | 0.159 |
|  |  |  |
| IBDQ total score at baseline (per 10-unit increase) | 0.98 (0.94, 1.02) | 0.36 |
| Mayo score at baseline | 0.98 (0.91, 1.06) | 0.675 |
| Adapted Mayo score at baseline | 0.96 (0.88, 1.05) | 0.382 |
| Sex (Male vs. Female) | 0.95 (0.73, 1.23) | 0.7 |
| Race (White vs. Other) | 1.02 (0.76, 1.39) | 0.874 |
| Prior surgery for IBD (Yes vs. No) | 0.46 (0.1, 2.12) | 0.32 |
| Smoking (Former smoker/Never smoked vs. Current Smoker) | 1.16 (0.67, 2.02) | 0.596 |
| Disease extent based on Montreal criteria at diagnosis | | |
| Extensive UC | 1 (reference) | |
| Left-sided UC | 0.61 (0.35, 1.05) | 0.073 |
| Other | 0.76 (0.5, 1.17) | 0.212 |
| Concomitant 5-ASA drugs at baseline (Yes vs. No) | 1 (0.66, 1.53) | 0.99 |
| Previous 5-ASA drug treatment (Yes vs. No) | 0.82 (0.56, 1.18) | 0.28 |
| Concomitant calcineurin inhibitor at baseline (Yes vs. No) | 1.17 (0.27, 5.03) | 0.834 |
| Previous calcineurin inhibitor treatment (Yes vs. No) | 1.3 (0.36, 4.72) | 0.688 |
| Concomitant 6-MP at baseline (Yes vs. No) | 1.03 (0.4, 2.66) | 0.954 |
| Previous 6-MP treatment (Yes vs. No) | 0.96 (0.39, 2.33) | 0.921 |
| Concomitant AZA at baseline (Yes vs. No) | 0.43 (0.24, 0.78) | 0.005 |
| Previous AZA treatment (Yes vs. No) | 0.49 (0.27, 0.92) | 0.026 |
| Concomitant MTX at baseline (Yes vs. No) | 1.92 (0.54, 6.73) | 0.311 |
| Previous MTX drug treatment (Yes vs. No) | 2.29 (0.68, 7.68) | 0.179 |
| Concomitant oral corticosteroid at baseline (Yes vs. No) | 1.2 (0.79, 1.81) | 0.387 |
| Previous oral corticosteroid treatment (Yes vs. No) | 0.98 (0.7, 1.39) | 0.932 |
| Prior exposure to anti-TNFs (Yes vs. No) | 0.72 (0.45, 1.15) | 0.171 |
| Prior failure to anti-TNFs (Yes vs. No) | 0.98 (0.55, 1.72) | 0.933 |
| Prior intolerance to anti-TNFs (Yes vs. No) | 0.6 (0.16, 2.2) | 0.438 |
| Previous exposure to biologic (Yes vs. No) | 0.72 (0.45, 1.15) | 0.171 |
| Abbreviations: CI, confidence interval; IBDQ, inflammatory bowel disease questionnaire; IBD, inflammatory bowel disease; CRP, C-reactive protein; FCP, fecal calprotectin; 5-ASA, 5-aminosalicylic acid; 6-MP, 6-mercaptopurine; AZA, azathioprine; MTX, methotrexate; TNF, tumor necrosis factor. | | |

# **Table S4.** Univariable regression analyses of patient-level factors contributing to clinical response (decrease in adapted MCS ≥ 2, a ≥ 35% reduction from baseline, and a ≥ 1-point decrease in RB or absolute RB subscore ≤ 1) for induction trials in ulcerative colitis.

| **Patient Characteristic** | **Odds Ratio (95% CI)** | **p-value** |
| --- | --- | --- |
| Age | 1 (0.99, 1.01) | 0.413 |
| Body mass index | 1.03 (1, 1.05) | 0.026 |
| Age at diagnosis | 1 (0.99, 1.02) | 0.592 |
| Disease duration at baseline | 1.01 (0.98, 1.04) | 0.471 |
| Albumin level at baseline (per 10-unit increase) | 1.38 (1.04, 1.82) | 0.026 |
| CRP level at baseline (per 10-unit increase) | 1.06 (0.95, 1.19) | 0.275 |
| FCP level at baseline (per 10-unit increase) | 1 (1, 1) | 0.789 |
|  |  |  |
| IBDQ total score at baseline (per 10-unit increase) | 1 (0.96, 1.03) | 0.927 |
| Mayo score at baseline | 0.93 (0.86, 1) | 0.063 |
| Adapted Mayo score at baseline | 0.92 (0.84, 1) | 0.064 |
| Sex (Male vs. Female) | 0.97 (0.75, 1.25) | 0.827 |
| Race (White vs. Other) | 1.06 (0.81, 1.38) | 0.668 |
| Prior surgery for IBD (Yes vs. No) | 0.4 (0.09, 1.83) | 0.237 |
| Smoking (Former smoker/Never smoked vs. Current Smoker) | 0.94 (0.56, 1.6) | 0.824 |
| Disease extent based on Montreal criteria at diagnosis | | |
| Extensive UC | 1 (reference) | |
| Left-sided UC | 0.74 (0.44, 1.24) | 0.253 |
| Other | 0.75 (0.49, 1.15) | 0.189 |
|  |  |  |
| Concomitant 5-ASA drugs at baseline (Yes vs. No) | 1.05 (0.73, 1.49) | 0.808 |
| Previous 5-ASA drug treatment (Yes vs. No) | 1.01 (0.79, 1.31) | 0.911 |
| Concomitant calcineurin inhibitor at baseline (Yes vs. No) | 1.12 (0.26, 4.76) | 0.883 |
| Previous calcineurin inhibitor treatment (Yes vs. No) | 1.27 (0.35, 4.56) | 0.717 |
| Concomitant 6-MP at baseline (Yes vs. No) | 1.01 (0.39, 2.58) | 0.99 |
| Previous 6-MP treatment (Yes vs. No) | 0.94 (0.39, 2.26) | 0.892 |
| Concomitant AZA at baseline (Yes vs. No) | 0.6 (0.35, 1.05) | 0.075 |
| Previous AZA treatment (Yes vs. No) | 0.73 (0.42, 1.28) | 0.271 |
| Concomitant MTX at baseline (Yes vs. No) | 1.91 (0.55, 6.69) | 0.309 |
| Previous MTX drug treatment (Yes vs. No) | 2.33 (0.7, 7.74) | 0.168 |
| Concomitant oral corticosteroid at baseline (Yes vs. No) | 1.03 (0.72, 1.49) | 0.853 |
| Previous oral corticosteroid treatment (Yes vs. No) | 1.07 (0.83, 1.38) | 0.613 |
| Prior exposure to anti-TNFs (Yes vs. No) | 0.85 (0.56, 1.29) | 0.437 |
| Prior failure to anti-TNFs (Yes vs. No) | 1.11 (0.67, 1.84) | 0.68 |
| Prior intolerance to anti-TNFs (Yes vs. No) | 0.51 (0.14, 1.82) | 0.297 |
| Previous exposure to biologic (Yes vs. No) | 0.85 (0.56, 1.29) | 0.437 |
| Abbreviations: CI, confidence interval; MCS Mayo Clinic Score; RB, rectal bleeding; IBDQ, inflammatory bowel disease questionnaire; IBD, inflammatory bowel disease; CRP, C-reactive protein; FCP, fecal calprotectin; 5-ASA, 5-aminosalicylic acid; 6-MP, 6-mercaptopurine; AZA, azathioprine; MTX, methotrexate; TNF, tumor necrosis factor. | | |

# **Table S5.** Univariable regression analyses of trial-level factors contributing to clinical response (trial definition) for induction trials in ulcerative colitis.

| **Trial Characteristic** | **Odds Ratio (95% CI)** | **p-value** |
| --- | --- | --- |
| Trial start year (After 2010 vs. 2010 and before) | 1.17 (0.57, 2.41) | 0.674 |
| Trial end year (After 2010 vs. 2010 and before) | 0.88 (0.6, 1.29) | 0.51 |
|  | | |
|  |  | |
| Trial design (multi-arm parallel vs. 2-arm parallel) | 0.93 (0.58, 1.48) | 0.763 |
|  |  |  |
| Study drug | | |
| Adalimumab | 1 (reference) | |
| Golimumab | 0.73 (0.49, 1.09) | 0.122 |
| Infliximab | 0.97 (0.49, 1.92) | 0.923 |
| Vedolizumab | 0.66 (0.43, 1.01) | 0.058 |
| Stratification factors (2 vs. 1) | 1.1 (0.7, 1.72) | 0.68 |
| Number of arms | | |
| 2 | 1 (reference) | |
| 3 | 0.99 (0.59, 1.66) | 0.966 |
| 4 | 0.83 (0.46, 1.49) | 0.528 |
| Trial phase (3 vs. 2/3) | 1.2 (0.78, 1.84) | 0.407 |
| Location | | |
| Asia | 1 (reference) | |
| Multi-continental | 0.99 (0.66, 1.49) | 0.968 |
| North America | 0.64 (0.35, 1.16) | 0.141 |
| Trial setting (Multicentre, single-country vs. Multicentre, multinational) | 1.09 (0.71, 1.68) | 0.686 |
|  | | |
|  |  | |
| Route of administration (SC vs. IV) | 1.24 (0.86, 1.8) | 0.253 |
|  |  |  |
| Blinding | | |
| Double | 1 (reference) | |
| Quadruple | 1.14 (0.8, 1.64) | 0.461 |
| Triple | 0.7 (0.41, 1.19) | 0.188 |
| Time of primary endpoint (> 6 weeks vs. ≤ 6 weeks) | 1.42 (1.06, 1.9) | 0.018 |
|  | | |
|  |  | |
|  |  |  |
|  |  |  |
| Number of centres (per 10-centre increase) | 0.98 (0.95, 1.01) | 0.269 |
| Number of follow-up visits | 1 (0.97, 1.03) | 0.897 |
| Abbreviations: CI, confidence interval; IV, intravenous; SC subcutaneous | | |

# **Table S6.** Univariable regression analyses of trial-level factors contributing to clinical response (decrease in adapted MCS ≥ 2, a ≥ 35% reduction from baseline, and a ≥ 1-point decrease in RB or absolute RB subscore ≤ 1) for induction trials in ulcerative colitis.

| **Trial Characteristic** | **Odds Ratio (95% CI)** | **p-value** |
| --- | --- | --- |
| Trial start year (After 2010 vs. 2010 and before) | 1.19 (0.66, 2.14) | 0.568 |
| Trial end year (After 2010 vs. 2010 and before) | 0.89 (0.68, 1.16) | 0.372 |
|  | | |
|  |  | |
| Trial design (Multi-arm parallel vs. 2-arm parallel) | 1.01 (0.74, 1.4) | 0.927 |
|  |  |  |
| Study drug | | |
| Adalimumab | 1 (reference) | |
| Golimumab | 0.99 (0.7, 1.41) | 0.965 |
| Infliximab | 1.14 (0.6, 2.17) | 0.696 |
| Vedolizumab | 0.85 (0.58, 1.24) | 0.397 |
| Stratification factors (2 vs. 1) | 0.88 (0.65, 1.2) | 0.428 |
| Number of arms | | |
| 2 | 1 (reference) | |
| 3 | 0.99 (0.67, 1.47) | 0.973 |
| 4 | 1.03 (0.66, 1.59) | 0.903 |
| Trial phase (3 vs. 2/3) | 0.97 (0.71, 1.31) | 0.83 |
| Location | | |
| Asia | 1 (reference) | |
| Multi-continental | 0.97 (0.71, 1.32) | 0.846 |
| North America | 0.66 (0.42, 1.03) | 0.068 |
| Trial setting (Multicentre, single-country vs. Multicentre, multinational) | 1.1 (0.8, 1.5) | 0.566 |
|  | | |
|  |  | |
|  |  |  |
| Route of administration (SC vs. IV) | 1.08 (0.82, 1.41) | 0.597 |
| Blinding | | |
| Double | 1 (reference) | |
| Quadruple | 1.06 (0.81, 1.39) | 0.651 |
| Triple | 0.7 (0.46, 1.05) | 0.088 |
| Time of primary endpoint (> 6 weeks vs. ≤ 6 weeks) | 1.16 (0.89, 1.52) | 0.281 |
|  | | |
|  |  | |
|  |  |  |
|  |  |  |
| Number of centres (per 10-centre increase) | 0.99 (0.97, 1.02) | 0.645 |
| Number of follow-up visits | 0.99 (0.97, 1.01) | 0.423 |
| Abbreviations: CI, confidence interval; IV, intravenous; SC subcutaneous | | |

# **Table S7.** Univariable regression analyses of patient-level factors contributing to clinical remission (trial definition) for induction trials in ulcerative colitis.

| **Patient Characteristic** | **Odds Ratio (95% CI)** | **p-value** |
| --- | --- | --- |
| Age | 1 (0.99, 1.02) | 0.749 |
| Body mass index | 1.01 (0.98, 1.06) | 0.455 |
| Age at diagnosis | 0.99 (0.97, 1.02) | 0.572 |
| Disease duration at baseline | 1.02 (0.98, 1.06) | 0.424 |
| Albumin level at baseline (per 10-unit increase) | 1.29 (0.8, 2.1) | 0.298 |
| CRP level at baseline (per 10-unit increase) | 0.83 (0.63, 1.1) | 0.192 |
| FCP level at baseline (per 10-unit increase) | 1 (1, 1) | 0.977 |
|  |  |  |
| IBDQ total score at baseline (per 10-unit increase) | 1.07 (1, 1.14) | 0.052 |
| Mayo score at baseline | 0.74 (0.64, 0.85) | <0.001 |
| Adapted Mayo score at baseline | 0.73 (0.63, 0.85) | <0.001 |
| Sex (Male vs. Female) | 0.73 (0.48, 1.12) | 0.155 |
| Race (White vs. Other) | 0.78 (0.5, 1.2) | 0.257 |
| Prior surgery for IBD (Yes vs. No) | N/A | N/A |
| Smoking (Former smoker/Never smoked vs. Current Smoker) | 0.75 (0.33, 1.7) | 0.491 |
| Disease extent based on Montreal criteria at diagnosis | | |
| Extensive UC | 1 (reference) | |
| Left-sided UC | 1.59 (0.64, 3.91) | 0.315 |
| Other | 0.77 (0.37, 1.61) | 0.487 |
|  |  |  |
| Concomitant 5-ASA drugs at baseline (Yes vs. No) | 1.34 (0.79, 2.27) | 0.282 |
| Previous 5-ASA drug treatment (Yes vs. No) | 1.04 (0.67, 1.6) | 0.877 |
| Concomitant calcineurin inhibitor at baseline (Yes vs. No) | 1.42 (0.17, 11.71) | 0.745 |
| Previous calcineurin inhibitor treatment (Yes vs. No) | 1.11 (0.14, 8.91) | 0.921 |
| Concomitant 6-MP at baseline (Yes vs. No) | 0.51 (0.07, 3.9) | 0.52 |
| Previous 6-MP treatment (Yes vs. No) | 0.9 (0.21, 3.94) | 0.894 |
| Concomitant AZA at baseline (Yes vs. No) | 0.39 (0.12, 1.33) | 0.133 |
| Previous AZA treatment (Yes vs. No) | 0.73 (0.31, 1.76) | 0.486 |
| Concomitant MTX at baseline (Yes vs. No) | 1.1 (0.14, 8.82) | 0.928 |
| Previous MTX drug treatment (Yes vs. No) | 1 (0.13, 7.93) | 0.999 |
| Concomitant oral corticosteroid at baseline (Yes vs. No) | 1.39 (0.83, 2.32) | 0.208 |
| Previous oral corticosteroid treatment (Yes vs. No) | 1.18 (0.69, 2.02) | 0.549 |
| Prior exposure to anti-TNFs (Yes vs. No) | 0.67 (0.3, 1.48) | 0.317 |
| Prior failure to anti-TNFs (Yes vs. No) | 0.8 (0.31, 2.04) | 0.644 |
| Prior intolerance to anti-TNFs (Yes vs. No) | N/A | N/A |
| Previous exposure to biologic (Yes vs. No) | 0.67 (0.3, 1.48) | 0.317 |
| Abbreviations: CI, confidence interval; IBDQ, inflammatory bowel disease questionnaire; IBD, inflammatory bowel disease; CRP, C-reactive protein; FCP, fecal calprotectin; 5-ASA, 5-aminosalicylic acid; 6-MP, 6-mercaptopurine; AZA, azathioprine; MTX, methotrexate; TNF, tumor necrosis factor. | | |

# **Table S8.** Univariable regression analyses of patient-level factors contributing to clinical remission (MES ≤ 1, a ≥ 1-point decrease in SF to achieve a SF ≤ 1, and RB = 0) for induction trials in ulcerative colitis.

| **Patient Characteristic** | **Odds Ratio (95% CI)** | **p-value** |
| --- | --- | --- |
| Age | 1 (0.99, 1.01) | 0.868 |
| Body mass index | 1.03 (0.99, 1.06) | 0.132 |
| Age at diagnosis | 0.99 (0.97, 1.01) | 0.292 |
| Disease duration at baseline | 1.02 (0.99, 1.06) | 0.251 |
| Albumin level at baseline (per 10-unit increase) | 1.21 (0.8, 1.84) | 0.363 |
| CRP level at baseline (per 10-unit increase) | 0.84 (0.67, 1.05) | 0.125 |
| FCP level at baseline (per 10-unit increase) | 1 (1, 1) | 0.555 |
|  |  |  |
| IBDQ total score at baseline (per 10-unit increase) | 1.05 (1, 1.11) | 0.065 |
| Mayo score at baseline | 0.85 (0.76, 0.95) | 0.005 |
| Adapted Mayo score at baseline | 0.83 (0.73, 0.95) | 0.005 |
| Sex (Male vs. Female) | 0.86 (0.6, 1.24) | 0.413 |
| Race (White vs. Other) | 0.9 (0.62, 1.31) | 0.593 |
| Prior surgery for IBD (Yes vs. No) | 0.73 (0.09, 5.74) | 0.764 |
| Smoking (Former smoker/Never smoked vs. Current Smoker) | 0.87 (0.42, 1.81) | 0.711 |
| Disease extent based on Montreal criteria at diagnosis | | |
| Extensive UC | 1 (reference) | |
| Left-sided UC | 0.95 (0.46, 1.97) | 0.899 |
| Other | 0.53 (0.26, 1.07) | 0.075 |
|  |  |  |
| Concomitant 5-ASA drugs at baseline (Yes vs. No) | 1.38 (0.88, 2.17) | 0.165 |
| Previous 5-ASA drug treatment (Yes vs. No) | 1.14 (0.75, 1.71) | 0.539 |
| Concomitant calcineurin inhibitor at baseline (Yes vs. No) | 0.84 (0.1, 7.36) | 0.873 |
| Previous calcineurin inhibitor treatment (Yes vs. No) | 0.65 (0.08, 5.38) | 0.692 |
| Concomitant 6-MP at baseline (Yes vs. No) | 1.64 (0.5, 5.32) | 0.412 |
| Previous 6-MP treatment (Yes vs. No) | 1.71 (0.58, 5.01) | 0.331 |
| Concomitant AZA at baseline (Yes vs. No) | 0.75 (0.34, 1.66) | 0.474 |
| Previous AZA treatment (Yes vs. No) | 0.71 (0.3, 1.66) | 0.428 |
| Concomitant MTX at baseline (Yes vs. No) | 1.72 (0.36, 8.26) | 0.5 |
| Previous MTX drug treatment (Yes vs. No) | 1.51 (0.32, 7.19) | 0.602 |
| Concomitant oral corticosteroid at baseline (Yes vs. No) | 1.37 (0.81, 2.29) | 0.237 |
| Previous oral corticosteroid treatment (Yes vs. No) | 1.18 (0.75, 1.86) | 0.477 |
| Prior exposure to anti-TNFs (Yes vs. No) | 0.97 (0.53, 1.76) | 0.91 |
| Prior failure to anti-TNFs (Yes vs. No) | 1.32 (0.67, 2.58) | 0.419 |
| Prior intolerance to anti-TNFs (Yes vs. No) | 0.53 (0.07, 4.09) | 0.543 |
| Previous exposure to biologic (Yes vs. No) | 0.97 (0.53, 1.76) | 0.91 |
| Abbreviations: Abbreviations: CI, confidence interval; MES, Mayo endoscopic subscore; SF, stool frequency; RB, rectal bleeding; IBDQ, inflammatory bowel disease questionnaire; IBD, inflammatory bowel disease; CRP, C-reactive protein; FCP, fecal calprotectin; 5-ASA, 5-aminosalicylic acid; 6-MP, 6-mercaptopurine; AZA, azathioprine; MTX, methotrexate; TNF, tumor necrosis factor. | | |

# **Table S9.** Univariable regression analyses of trial-level factors contributing to clinical remission (trial definition) for induction trials in ulcerative colitis.

| **Trial Characteristic** | **Odds Ratio (95% CI)** | **p-value** |
| --- | --- | --- |
| Trial start year (After 2010 vs. 2010 and before) | 1.17 (0.45, 3.03) | 0.742 |
| Trial end year (After 2010 vs. 2010 and before) | 1.13 (0.73, 1.76) | 0.59 |
|  | | |
|  |  | |
|  |  |  |
| Trial design (Multi-arm parallel vs. vs. 2-arm parallel) | 0.9 (0.56, 1.47) | 0.686 |
| Study drug | | |
| Adalimumab | 1 (reference) | |
| Golimumab | 0.76 (0.46, 1.26) | 0.29 |
| Infliximab | 1.05 (0.4, 2.78) | 0.924 |
| Vedolizumab | 0.85 (0.48, 1.5) | 0.569 |
| Stratification factors (2 vs. 1) | 0.92 (0.56, 1.5) | 0.729 |
| Number of arms | | |
| 2 | 1 (reference) | |
| 3 | 0.99 (0.6, 1.64) | 0.959 |
| 4 | 0.78 (0.45, 1.38) | 0.399 |
| Trial phase (3 vs. 2/3) | 1.26 (0.78, 2.04) | 0.336 |
| Location | | |
| Asia | 1 (reference) | |
| Multi-continental | 0.72 (0.44, 1.17) | 0.189 |
| North America | 0.44 (0.19, 1) | 0.049 |
| Trial setting (multicentre, single-country vs. multicentre, multinational) | 1.47 (0.91, 2.37) | 0.113 |
|  | | |
|  |  | |
|  |  |  |
| Route of administration (SC vs. IV) | 0.95 (0.61, 1.5) | 0.842 |
| Blinding | | |
| Double | 1 (reference) | |
| Quadruple | 1.2 (0.76, 1.89) | 0.435 |
| Triple | 0.62 (0.28, 1.37) | 0.233 |
|  | | |
|  |  | |
|  |  |  |
|  |  |  |
| Time of primary endpoint (> 6 weeks vs. ≤ 6 weeks) | 1.51 (0.97, 2.35) | 0.069 |
| Number of centres (per 10-centre increase) | 0.97 (0.93, 1.01) | 0.09 |
| Number of follow-up visits | 1.01 (0.98, 1.05) | 0.365 |
| Abbreviations: CI, confidence interval; IV, intravenous; SC subcutaneous | | |

# **Table S10.** Univariable regression analyses of trial-level factors contributing to clinical remission (MES ≤ 1, a ≥ 1-point decrease in SF to achieve a SF ≤ 1, and RB = 0) for induction trials in ulcerative colitis.

| **Trial Characteristic** | **Odds Ratio (95% CI)** | **p-value** |
| --- | --- | --- |
| Trial start year (After 2010 vs. 2010 and before) | 2.17 (0.31, 15.13) | 0.881 |
| Trial end year (After 2010 vs. 2010 and before) | 0.34 (0.03, 4.63) | 0.731 |
|  | | |
|  |  | |
|  |  |  |
| Trial design (multi-arm parallel vs. 2-arm parallel) | 1.1 (0.63, 1.93) | 0.737 |
| Study drug | | |
| Adalimumab | 1 (reference) | |
| Golimumab | 0.92 (0.47, 1.81) | 0.805 |
| Infliximab | 0.93 (0.31, 2.81) | 0.898 |
| Vedolizumab | 1.12 (0.56, 2.24) | 0.754 |
| Stratification factors (2 vs. 1) | 0.8 (0.47, 1.37) | 0.417 |
| Number of arms | | |
| 2 | 1 (reference) | |
| 3 | 1.17 (0.63, 2.2) | 0.62 |
| 4 | 0.98 (0.48, 2) | 0.966 |
| Trial phase (3 vs. 2/3) | 1.14 (0.67, 1.92) | 0.628 |
| Location | | |
| Asia | 1 (reference) | |
| Multi-continental | 0.73 (0.48, 1.11) | 0.139 |
| North America | 0.49 (0.25, 0.96) | 0.037 |
| Trial setting (multicentre, single-country vs. multicentre, multinational) | 1.45 (0.96, 2.2) | 0.078 |
| Route of administration (SC vs. IV) | 0.86 (0.54, 1.37) | 0.525 |
| Blinding | | |
| Double | 1 (reference) | |
| Quadruple | 1.36 (0.88, 2.08) | 0.162 |
| Triple | 0.74 (0.37, 1.47) | 0.385 |
|  |  |  |
| Time of primary endpoint (> 6 weeks vs. ≤ 6 weeks) | 1.38 (0.92, 2.07) | 0.121 |
| Number of centres (per 10-centre increase) | 0.98 (0.94, 1.01) | 0.175 |
| Number of follow-up visits | 1.01 (0.97, 1.04) | 0.763 |
| Abbreviations: CI, confidence interval; IV, intravenous; SC subcutaneous | | |

# **Table S11.** Univariable regression analyses of patient-level factors contributing to clinical response (trial definition) for maintenance trials in ulcerative colitis.

| **Patient Characteristic** | **Odds Ratio (95% CI)** | **p-value** |
| --- | --- | --- |
| Age | 1.01 (0.99, 1.02) | 0.338 |
| Body mass index | 1.01 (0.97, 1.05) | 0.696 |
| Age at diagnosis | 1 (0.98, 1.02) | 0.979 |
| Disease duration at baseline | 1.01 (0.97, 1.05) | 0.56 |
| Albumin level at baseline (per 10-unit increase) | 1.5 (0.97, 2.3) | 0.065 |
| CRP level at baseline (per 10-unit increase) | 0.98 (0.8, 1.18) | 0.802 |
| FCP level at baseline (per 10-unit increase) | 1 (1, 1) | 0.8 |
|  |  |  |
| IBDQ total score at baseline (per 10-unit increase) | 1.05 (0.98, 1.12) | 0.163 |
| Mayo score at baseline | 1.01 (0.9, 1.13) | 0.9 |
| Adapted Mayo score at baseline | 0.99 (0.87, 1.13) | 0.863 |
| Sex (Male vs. Female) | 0.85 (0.58, 1.25) | 0.399 |
| Race (White vs. Other) | 0.62 (0.32, 1.21) | 0.162 |
| Prior surgery for IBD (Yes vs. No) | 0.76 (0.28, 2.11) | 0.603 |
| Smoking (Former smoker/Never smoked vs. Current Smoker) | 2.35 (0.96, 5.77) | 0.061 |
| Disease extent based on Montreal criteria at diagnosis | | |
| Extensive UC | 1 (reference) | |
| Left-sided UC | 1.9 (1.02, 3.54) | 0.043 |
| Other | 0.85 (0.44, 1.64) | 0.632 |
| Concomitant 5-ASA drugs at baseline (Yes vs. No) | 1.44 (0.74, 2.81) | 0.289 |
| Previous 5-ASA drug treatment (Yes vs. No) | 0.94 (0.4, 2.2) | 0.888 |
| Concomitant calcineurin inhibitor at baseline (Yes vs. No) | 1.18 (0.18, 7.77) | 0.862 |
| Previous calcineurin inhibitor treatment (Yes vs. No) | 1.96 (0.45, 8.61) | 0.374 |
| Concomitant 6-MP at baseline (Yes vs. No) | 1.33 (0.55, 3.22) | 0.529 |
| Previous 6-MP treatment (Yes vs. No) | 1.01 (0.34, 3) | 0.98 |
| Concomitant AZA at baseline (Yes vs. No) | 1.35 (0.75, 2.44) | 0.315 |
| Previous AZA treatment (Yes vs. No) | 1 (0.51, 1.97) | 0.998 |
| Concomitant MTX at baseline (Yes vs. No) | N/A | N/A |
| Previous MTX drug treatment (Yes vs. No) | 0.45 (0.06, 3.65) | 0.458 |
| Concomitant oral corticosteroid at baseline (Yes vs. No) | 0.63 (0.38, 1.02) | 0.062 |
| Previous oral corticosteroid treatment (Yes vs. No) | 0.72 (0.39, 1.34) | 0.298 |
| Prior exposure to anti-TNFs (Yes vs. No) | 1.05 (0.56, 1.99) | 0.88 |
| Prior failure to anti-TNFs (Yes vs. No) | 0.89 (0.42, 1.91) | 0.765 |
| Prior intolerance to anti-TNFs (Yes vs. No) | 0.69 (0.18, 2.59) | 0.58 |
| Previous exposure to biologic (Yes vs. No) | 1.05 (0.56, 1.99) | 0.88 |
| Abbreviations: CI, confidence interval; IBDQ, inflammatory bowel disease questionnaire; IBD, inflammatory bowel disease; CRP, C-reactive protein; FCP, fecal calprotectin; 5-ASA, 5-aminosalicylic acid; 6-MP, 6-mercaptopurine; AZA, azathioprine; MTX, methotrexate; TNF, tumor necrosis factor. | | |

# **Table S12.** Univariable regression analyses of patient-level factors contributing to clinical response (decrease in adapted MCS ≥ 2, a ≥ 35% reduction from baseline, and a ≥ 1-point decrease in RB or absolute RB subscore ≤ 1) for maintenance trials in ulcerative colitis.

| **Patient Characteristic** | **Odds Ratio (95% CI)** | **p-value** |
| --- | --- | --- |
| Age | 1.01 (1, 1.02) | 0.146 |
| Body mass index | 1.01 (0.97, 1.05) | 0.762 |
| Age at diagnosis | 1 (0.98, 1.02) | 0.892 |
| Disease duration at baseline | 1.02 (0.99, 1.06) | 0.22 |
| Albumin level at baseline (per 10-unit increase) | 1.48 (0.96, 2.28) | 0.075 |
| CRP level at baseline (per 10-unit increase) | 0.93 (0.77, 1.13) | 0.489 |
| FCP level at baseline (per 10-unit increase) | 1 (1, 1) | 0.67 |
|  |  |  |
| IBDQ total score at baseline (per 10-unit increase) | 1.04 (0.97, 1.12) | 0.215 |
| Mayo score at baseline | 1 (0.89, 1.13) | 0.947 |
| Adapted Mayo score at baseline | 1 (0.87, 1.14) | 0.951 |
| Sex (Male vs. Female) | 0.91 (0.62, 1.35) | 0.652 |
| Race (White vs. Other) | 0.62 (0.3, 1.26) | 0.184 |
| Prior surgery for IBD (Yes vs. No) | 0.78 (0.28, 2.17) | 0.64 |
| Smoking (Former smoker/Never smoked vs. Current Smoker) | 2.07 (0.89, 4.81) | 0.091 |
| Disease extent based on Montreal criteria at diagnosis | | |
| Extensive UC | 1 (reference) | |
| Left-sided UC | 1.54 (0.82, 2.89) | 0.183 |
| Other | 0.9 (0.46, 1.75) | 0.748 |
| Concomitant 5-ASA drugs at baseline (Yes vs. No) | 1.2 (0.59, 2.46) | 0.613 |
| Previous 5-ASA drug treatment (Yes vs. No) | 0.91 (0.32, 2.61) | 0.868 |
| Concomitant calcineurin inhibitor at baseline (Yes vs. No) | 1.54 (0.22, 10.83) | 0.663 |
| Previous calcineurin inhibitor treatment (Yes vs. No) | 2.43 (0.51, 11.52) | 0.262 |
| Concomitant 6-MP at baseline (Yes vs. No) | 1.08 (0.43, 2.71) | 0.878 |
| Previous 6-MP treatment (Yes vs. No) | 1.06 (0.36, 3.13) | 0.918 |
| Concomitant AZA at baseline (Yes vs. No) | 1.15 (0.63, 2.09) | 0.656 |
| Previous AZA treatment (Yes vs. No) | 0.84 (0.41, 1.73) | 0.641 |
| Concomitant MTX at baseline (Yes vs. No) | N/A | N/A |
| Previous MTX drug treatment (Yes vs. No) | 0.47 (0.06, 3.77) | 0.476 |
| Concomitant oral corticosteroid at baseline (Yes vs. No) | 0.66 (0.4, 1.08) | 0.098 |
| Previous oral corticosteroid treatment (Yes vs. No) | 0.88 (0.46, 1.66) | 0.693 |
| Prior exposure to anti-TNFs (Yes vs. No) | 0.88 (0.46, 1.67) | 0.686 |
| Prior failure to anti-TNFs (Yes vs. No) | 0.58 (0.26, 1.29) | 0.184 |
| Prior intolerance to anti-TNFs (Yes vs. No) | 0.72 (0.2, 2.59) | 0.62 |
| Previous exposure to biologic (Yes vs. No) | 0.88 (0.46, 1.67) | 0.686 |
| Abbreviations: CI, confidence interval; MCS Mayo Clinic Score; RB, rectal bleeding; IBDQ, inflammatory bowel disease questionnaire; IBD, inflammatory bowel disease; CRP, C-reactive protein; FCP, fecal calprotectin; 5-ASA, 5-aminosalicylic acid; 6-MP, 6-mercaptopurine; AZA, azathioprine; MTX, methotrexate; TNF, tumor necrosis factor. | | |

# **Table S13.** Univariable regression analyses of trial-level factors contributing to clinical response (trial definition) for maintenance trials in ulcerative colitis.

| **Trial Characteristic** | **Odds Ratio (95% CI)** | **p-value** |
| --- | --- | --- |
| Trial start year (After 2010 vs. 2010 and before) | 2.11 (0.61, 7.35) | 0.24 |
| Trial end year (After 2010 vs. 2010 and before) | 2 (0.39, 10.2) | 0.406 |
|  | | |
|  |  | |
|  |  |  |
| Trial design (multi-arm parallel vs. 2-arm parallel) | 0.81 (0.21, 3.13) | 0.763 |
| Study drug | | |
| Adalimumab | 1 (reference) | |
| Golimumab | 1.11 (0.36, 3.37) | 0.858 |
| Infliximab | 6.69 (2.78, 16.08) | <0.001 |
| Vedolizumab | 1.97 (1.03, 3.79) | 0.041 |
| Stratification factors (2 vs. 1) | 0.28 (0.14, 0.56) | <0.001 |
|  | | |
|  |  | |
|  |  |  |
| Number of arms (3 vs. 2) | 0.81 (0.21, 3.13) | 0.763 |
|  |  |  |
| Location | | |
| Asia | 1 (reference) | |
| Multi-continental | 0.46 (0.07, 3.06) | 0.422 |
| North America | 0.66 (0.1, 4.47) | 0.669 |
| Trial setting (multicentre, single-country vs. multicentre, multinational) | 1.83 (0.51, 6.57) | 0.353 |
| Route of administration (SC vs. IV) | 0.35 (0.14, 0.87) | 0.023 |
| Blinding | | |
| Double | 1 (reference) | |
| Quadruple | 0.85 (0.15, 4.8) | 0.851 |
| Triple | 0.73 (0.08, 6.45) | 0.775 |
|  |  |  |
| Time of primary endpoint (> 6 weeks vs. ≤ 6 weeks) | 1.28 (0.22, 7.63) | 0.784 |
| Number of centres (per 10-centre increase) | 0.87 (0.77, 0.99) | 0.032 |
| Number of follow-up visits | 0.93 (0.85, 1.01) | 0.094 |
| Abbreviations: CI, confidence interval; IV, intravenous; SC subcutaneous | | |

# **Table S14.** Univariable regression analyses of trial-level factors contributing to clinical response (decrease in adapted MCS ≥ 2, a ≥ 35% reduction from baseline, and a ≥ 1-point decrease in RB or absolute RB subscore ≤ 1) for maintenance trials in ulcerative colitis.

| **Trial Characteristic** | **Odds Ratio (95% CI)** | **p-value** |
| --- | --- | --- |
| Trial start year (After 2010 vs. 2010 and before) | 1.68 (0.27, 10.49) | 0.577 |
| Trial end year (After 2010 vs. 2010 and before) | 2.43 (0.28, 21.35) | 0.425 |
|  | | |
|  |  | |
|  |  |  |
| Trial design (multi-arm parallel vs. 2-arm parallel | 1.06 (0.18, 6.31) | 0.946 |
| Study drug | | |
| Adalimumab | 1 (reference) | |
| Golimumab | 1.15 (0.14, 9.53) | 0.898 |
| Infliximab | 7.55 (1.02, 56.02) | 0.048 |
| Vedolizumab | 3.36 (0.67, 16.8) | 0.14 |
|  |  |  |
| Stratification factors (2 vs. 1) | 0.16 (0.1, 0.25) | <0.001 |
|  | | |
|  |  | |
|  |  |  |
| Number of arms (3 vs. 2) | 1.06 (0.18, 6.31) | 0.946 |
|  |  |  |
| Location | | |
| Asia | 1 (reference) | |
| Multi-continental | 0.36 (0.03, 4.26) | 0.418 |
| North America | 0.52 (0.04, 6.19) | 0.603 |
| Trial setting (multicentre, single-country vs. multicentre, multinational) | 2.34 (0.44, 12.3) | 0.317 |
| Route of administration (SC vs. IV) | 0.24 (0.08, 0.74) | 0.013 |
| Blinding | | |
| Double | 1 (reference) | |
| Quadruple | 1.37 (0.15, 12.83) | 0.783 |
| Triple | 0.73 (0.04, 12.23) | 0.827 |
|  |  |  |
| Time of primary endpoint (> 6 weeks vs. ≤ 6 weeks) | 1.56 (0.15, 15.97) | 0.707 |
| Number of centres (per 10-centre increase) | 0.86 (0.71, 1.05) | 0.142 |
| Number of follow-up visits | 0.92 (0.82, 1.04) | 0.193 |
| Abbreviations: CI, confidence interval; IV, intravenous; SC subcutaneous | | |

# **Table S15.** Univariable regression analyses of patient-level factors contributing to clinical remission (trial definition) for maintenance trials in ulcerative colitis.

| **Patient Characteristic** | **Odds Ratio (95% CI)** | **p-value** |
| --- | --- | --- |
| Age | 1 (0.98, 1.02) | 0.833 |
| Body mass index | 1 (0.96, 1.05) | 0.851 |
| Age at diagnosis | 1.01 (0.98, 1.03) | 0.565 |
| Disease duration at baseline | 0.97 (0.93, 1.03) | 0.321 |
| Albumin level at baseline (per 10-unit increase) | 1.92 (1.09, 3.39) | 0.024 |
| CRP level at baseline (per 10-unit increase) | 1.02 (0.82, 1.27) | 0.85 |
| FCP level at baseline (per 10-unit increase) | 1 (1, 1) | 0.878 |
|  |  |  |
| IBDQ total score at baseline (per 10-unit increase) | 1.05 (0.95, 1.14) | 0.339 |
| Mayo score at baseline | 0.94 (0.81, 1.09) | 0.384 |
| Adapted Mayo score at baseline | 0.93 (0.78, 1.09) | 0.357 |
| Sex (Male vs. Female) | 0.96 (0.59, 1.57) | 0.874 |
| Race (White vs. Other) | 0.62 (0.32, 1.22) | 0.167 |
| Prior surgery for IBD (Yes vs. No) | 0.78 (0.21, 2.86) | 0.704 |
| Smoking (Former smoker/Never smoked vs. Current Smoker) | 2.04 (0.69, 6.04) | 0.2 |
| Disease extent based on Montreal criteria at diagnosis | | |
| Extensive UC | 1 (reference) | |
| Left-sided UC | 2.85 (1.5, 5.41) | 0.001 |
| Other | 0.68 (0.31, 1.52) | 0.353 |
| Concomitant 5-ASA drugs at baseline (Yes vs. No) | 1.5 (0.75, 3.02) | 0.252 |
| Previous 5-ASA drug treatment (Yes vs. No) | 0.78 (0.27, 2.27) | 0.65 |
| Concomitant calcineurin inhibitor at baseline (Yes vs. No) | N/A | N/A |
| Previous calcineurin inhibitor treatment (Yes vs. No) | 0.85 (0.1, 7.37) | 0.884 |
| Concomitant 6-MP at baseline (Yes vs. No) | 1.53 (0.57, 4.11) | 0.396 |
| Previous 6-MP treatment (Yes vs. No) | 1.04 (0.28, 3.92) | 0.949 |
| Concomitant AZA at baseline (Yes vs. No) | 0.7 (0.32, 1.52) | 0.369 |
| Previous AZA treatment (Yes vs. No) | 0.65 (0.26, 1.59) | 0.345 |
| Concomitant MTX at baseline (Yes vs. No) | N/A | N/A |
| Previous MTX drug treatment (Yes vs. No) | N/A | N/A |
| Concomitant oral corticosteroid at baseline (Yes vs. No) | 0.7 (0.39, 1.25) | 0.223 |
| Previous oral corticosteroid treatment (Yes vs. No) | 0.69 (0.32, 1.48) | 0.342 |
| Prior exposure to anti-TNFs (Yes vs. No) | 0.65 (0.29, 1.46) | 0.295 |
| Prior failure to anti-TNFs (Yes vs. No) | 0.43 (0.15, 1.25) | 0.121 |
| Prior intolerance to anti-TNFs (Yes vs. No) | 0.79 (0.17, 3.72) | 0.766 |
| Previous exposure to biologic (Yes vs. No) | 0.65 (0.29, 1.46) | 0.295 |
| Abbreviations: CI, confidence interval; IBDQ, inflammatory bowel disease questionnaire; IBD, inflammatory bowel disease; CRP, C-reactive protein; FCP, fecal calprotectin; 5-ASA, 5-aminosalicylic acid; 6-MP, 6-mercaptopurine; AZA, azathioprine; MTX, methotrexate; TNF, tumor necrosis factor. | | |

# **Table S16.** Univariable regression analyses of patient-level factors contributing to clinical remission (MES ≤ 1, a ≥ 1-point decrease in SF to achieve a SF ≤ 1, and RB = 0) for maintenance trials in ulcerative colitis.

| **Patient Characteristic** | **Odds Ratio (95% CI)** | **p-value** |
| --- | --- | --- |
| Age | 1 (0.98, 1.01) | 0.699 |
| Body mass index | 1 (0.95, 1.05) | 0.896 |
| Age at diagnosis | 1 (0.98, 1.02) | 0.927 |
| Disease duration at baseline | 0.98 (0.93, 1.03) | 0.376 |
| Albumin level at baseline (per 10-unit increase) | 2.33 (1.29, 4.19) | 0.005 |
| CRP level at baseline (per 10-unit increase) | 1.03 (0.84, 1.28) | 0.761 |
| FCP level at baseline (per 10-unit increase) | 1 (1, 1) | 0.844 |
|  |  |  |
| IBDQ total score at baseline (per 10-unit increase) | 1.06 (0.96, 1.16) | 0.229 |
| Mayo score at baseline | 0.98 (0.85, 1.14) | 0.829 |
| Adapted Mayo score at baseline | 0.95 (0.81, 1.12) | 0.551 |
| Sex (Male vs. Female) | 1.09 (0.66, 1.78) | 0.745 |
| Race (White vs. Other) | 0.55 (0.27, 1.1) | 0.091 |
| Prior surgery for IBD (Yes vs. No) | 0.88 (0.25, 3.11) | 0.841 |
| Smoking (Former smoker/Never smoked vs. Current Smoker) | 2.01 (0.68, 5.95) | 0.207 |
| Disease extent based on Montreal criteria at diagnosis | | |
| Extensive UC | 1 (reference) | |
| Left-sided UC | 2.21 (1.1, 4.45) | 0.026 |
| Other | 0.73 (0.33, 1.64) | 0.448 |
| Concomitant 5-ASA drugs at baseline (Yes vs. No) | 1.44 (0.7, 2.97) | 0.321 |
| Previous 5-ASA drug treatment (Yes vs. No) | 0.71 (0.22, 2.29) | 0.571 |
| Concomitant calcineurin inhibitor at baseline (Yes vs. No) | N/A | N/A |
| Previous calcineurin inhibitor treatment (Yes vs. No) | 0.72 (0.08, 6.33) | 0.768 |
| Concomitant 6-MP at baseline (Yes vs. No) | 2 (0.77, 5.17) | 0.155 |
| Previous 6-MP treatment (Yes vs. No) | 1.32 (0.39, 4.41) | 0.656 |
| Concomitant AZA at baseline (Yes vs. No) | 0.99 (0.48, 2.04) | 0.979 |
| Previous AZA treatment (Yes vs. No) | 0.91 (0.4, 2.1) | 0.826 |
| Concomitant MTX at baseline (Yes vs. No) | N/A | N/A |
| Previous MTX drug treatment (Yes vs. No) | N/A | N/A |
| Concomitant oral corticosteroid at baseline (Yes vs. No) | 0.71 (0.4, 1.26) | 0.242 |
| Previous oral corticosteroid treatment (Yes vs. No) | 0.63 (0.3, 1.35) | 0.237 |
| Prior exposure to anti-TNFs (Yes vs. No) | 0.89 (0.41, 1.94) | 0.765 |
| Prior failure to anti-TNFs (Yes vs. No) | 0.8 (0.32, 2.04) | 0.647 |
| Prior intolerance to anti-TNFs (Yes vs. No) | 0.35 (0.04, 2.78) | 0.321 |
| Previous exposure to biologic (Yes vs. No) | 0.89 (0.41, 1.94) | 0.765 |
| Abbreviations: CI, confidence interval; MES, Mayo endoscopic subscore; SF, stool frequency; RB, rectal bleeding; IBDQ, inflammatory bowel disease questionnaire; IBD, inflammatory bowel disease; CRP, C-reactive protein; FCP, fecal calprotectin; 5-ASA, 5-aminosalicylic acid; 6-MP, 6-mercaptopurine; AZA, azathioprine; MTX, methotrexate; TNF, tumor necrosis factor. | | |

# **Table S17.** Univariable regression analyses of trial-level factors contributing to clinical remission (trial definition) for maintenance trials in ulcerative colitis.

| **Trial Characteristic** | **Odds Ratio (95% CI)** | **p-value** |
| --- | --- | --- |
| Trial start year (After 2010 vs. 2010 and before) | 1.16 (0.37, 3.66) | 0.794 |
| Trial end year (After 2010 vs. 2010 and before) | 1.97 (0.66, 5.83) | 0.221 |
|  | | |
|  |  | |
|  |  |  |
| Trial design (Multi-arm parallel vs. 2-arm parallel) | 1.37 (0.52, 3.6) | 0.527 |
| Study drug | | |
| Adalimumab | 1 (reference) | |
| Golimumab | 2.18 (0.67, 7.13) | 0.196 |
| Infliximab | 1.89 (0.66, 5.46) | 0.238 |
| Vedolizumab | 2.9 (1.42, 5.9) | 0.003 |
| Stratification factors (2 vs. 1) | 0.54 (0.23, 1.3) | 0.172 |
|  | | |
|  |  | |
| Number of arms (3 vs. 2) | 1.37 (0.52, 3.6) | 0.527 |
|  |  |  |
|  |  |  |
| Location | | |
| Asia | 1 (reference) | |
| Multi-continental | 0.52 (0.13, 2.01) | 0.344 |
| North America | 1.05 (0.27, 4.11) | 0.943 |
| Trial setting (Multicentre, single-country vs. Multicentre, multinational) | 1.36 (0.49, 3.77) | 0.551 |
| Route of administration (SC vs. IV) | 0.44 (0.23, 0.83) | 0.011 |
| Blinding | | |
| Double | 1 (reference) | |
| Quadruple | 1.35 (0.37, 4.87) | 0.649 |
| Triple | 1.41 (0.29, 6.93) | 0.672 |
|  |  |  |
| Time of primary endpoint (> 6 weeks vs. ≤ 6 weeks) | 0.81 (0.22, 3.05) | 0.758 |
| Number of centres (per 10-centre increase) | 0.96 (0.83, 1.1) | 0.565 |
| Number of follow-up visits | 0.99 (0.91, 1.07) | 0.746 |
| Abbreviations: CI, confidence interval; IV, intravenous; SC subcutaneous | | |

# **Table S18.** Univariable regression analyses of trial-level factors contributing to clinical remission (MES ≤ 1, a ≥ 1-point decrease in SF to achieve a SF ≤ 1, and RB = 0) for maintenance trials in ulcerative colitis.

| **Trial Characteristic** | **Odds Ratio (95% CI)** | **p-value** |
| --- | --- | --- |
| Trial start year (After 2010 vs. 2010 and before) | 0.9 (0.26, 3.12) | 0.863 |
| Trial end year (After 2010 vs. 2010 and before) | 1.78 (0.49, 6.5) | 0.384 |
|  | | |
|  |  | |
|  |  |  |
| Trial design (Multi-arm parallel vs. 2-arm parallel) | 1.58 (0.57, 4.4) | 0.377 |
| Study drug | | |
| Adalimumab | 1 (reference) | |
| Golimumab | 1.56 (0.32, 7.69) | 0.585 |
| Infliximab | 1.47 (0.34, 6.41) | 0.609 |
| Vedolizumab | 2.78 (0.95, 8.11) | 0.062 |
| Stratification factors (2 vs. 1) | 0.45 (0.18, 1.13) | 0.09 |
|  | | |
|  |  | |
|  |  |  |
| Number of arms (3 vs. 2) | 1.58 (0.57, 4.4) | 0.377 |
|  |  |  |
| Location | | |
| Asia | 1 (reference) | |
| Multi-continental | 0.55 (0.12, 2.59) | 0.451 |
| North America | 0.87 (0.18, 4.21) | 0.867 |
| Trial setting (Multicentre, single-country vs. Multicentre, multinational) | 1.47 (0.5, 4.34) | 0.489 |
| Route of administration (SC vs. IV) | 0.46 (0.2, 1.08) | 0.074 |
| Blinding | | |
| Double | 1 (reference) | |
| Quadruple | 1.7 (0.46, 6.36) | 0.428 |
| Triple | 1.29 (0.25, 6.66) | 0.764 |
|  |  |  |
| Time of primary endpoint (> 6 weeks vs. ≤ 6 weeks) | 0.99 (0.23, 4.28) | 0.995 |
| Number of centres (per 10-centre increase) | 0.97 (0.83, 1.13) | 0.686 |
| Number of follow-up visits | 0.99 (0.91, 1.09) | 0.872 |
| Abbreviations: CI, confidence interval; IV, intravenous; SC subcutaneous | | |

# **Table S19.** Univariable regression analyses of patient-level factors contributing to endoscopic response (MES ≤ 1) for induction trials in ulcerative colitis.

| **Patient Characteristic** | **Odds Ratio (95% CI)** | **p-value** |
| --- | --- | --- |
| Age | 1 (0.99, 1.01) | 0.45 |
| Body mass index | 1.03 (1.01, 1.06) | 0.015 |
| Age at diagnosis | 0.99 (0.98, 1) | 0.153 |
| Disease duration at baseline | 1.02 (0.99, 1.04) | 0.212 |
| Albumin level at baseline (per 10-unit increase) | 1.8 (1.31, 2.48) | <0.001 |
| CRP level at baseline (per 10-unit increase) | 0.92 (0.8, 1.05) | 0.233 |
| FCP level at baseline (per 10-unit increase) | 1 (1, 1) | 0.365 |
| IBDQ total score at baseline (per 10-unit increase) | 1.02 (0.98, 1.07) | 0.343 |
| Mayo score at baseline | 0.72 (0.66, 0.79) | <0.001 |
| Adapted Mayo score at baseline | 0.69 (0.63, 0.77) | <0.001 |
| Sex (Male vs. Female) | 0.67 (0.51, 0.87) | 0.003 |
| Race (White vs. Other) | 1.22 (0.9, 1.65) | 0.193 |
| Prior surgery for IBD (Yes vs. No) | 0.22 (0.03, 1.73) | 0.151 |
| Smoking (Former smoker/Never smoked vs. Current Smoker) | 0.73 (0.43, 1.24) | 0.245 |
| Disease extent based on Montreal criteria at diagnosis | | |
| Extensive UC | 1 (reference) | |
| Left-sided UC | 0.92 (0.52, 1.61) | 0.759 |
| Other | 0.94 (0.61, 1.46) | 0.8 |
| Concomitant 5-ASA drugs at baseline (Yes vs. No) | 0.73 (0.47, 1.13) | 0.155 |
| Previous 5-ASA drug treatment (Yes vs. No) | 0.85 (0.62, 1.17) | 0.328 |
| Concomitant calcineurin inhibitor at baseline (Yes vs. No) | 1.4 (0.32, 6.04) | 0.652 |
| Previous calcineurin inhibitor treatment (Yes vs. No) | 0.94 (0.24, 3.7) | 0.926 |
| Concomitant 6-MP at baseline (Yes vs. No) | 1.26 (0.48, 3.27) | 0.638 |
| Previous 6-MP treatment (Yes vs. No) | 1.11 (0.46, 2.7) | 0.816 |
| Concomitant AZA at baseline (Yes vs. No) | 0.48 (0.26, 0.87) | 0.016 |
| Previous AZA treatment (Yes vs. No) | 0.54 (0.29, 0.98) | 0.043 |
| Concomitant MTX at baseline (Yes vs. No) | 0.91 (0.23, 3.58) | 0.894 |
| Previous MTX drug treatment (Yes vs. No) | 1.76 (0.53, 5.89) | 0.358 |
| Concomitant oral corticosteroid at baseline (Yes vs. No) | 1.39 (0.85, 2.25) | 0.188 |
| Previous oral corticosteroid treatment (Yes vs. No) | 1.11 (0.76, 1.61) | 0.585 |
| Prior exposure to anti-TNFs (Yes vs. No) | 0.82 (0.51, 1.32) | 0.409 |
| Prior failure to anti-TNFs (Yes vs. No) | 0.86 (0.48, 1.53) | 0.608 |
| Prior intolerance to anti-TNFs (Yes vs. No) | 1.38 (0.45, 4.27) | 0.571 |
| Previous exposure to biologic (Yes vs. No) | 0.82 (0.51, 1.32) | 0.409 |
| Abbreviations: Abbreviations: CI, confidence interval; MES, Mayo endoscopic subscore; SF, stool frequency; RB, rectal bleeding; IBDQ, inflammatory bowel disease questionnaire; IBD, inflammatory bowel disease; CRP, C-reactive protein; FCP, fecal calprotectin; 5-ASA, 5-aminosalicylic acid; 6-MP, 6-mercaptopurine; AZA, azathioprine; MTX, methotrexate; TNF, tumor necrosis factor. | | |

# **Table S20.** Univariable regression analyses of trial-level factors contributing to endoscopic response (MES ≤ 1) for induction trials in ulcerative colitis.

| **Trial Characteristic** | **Odds Ratio (95% CI)** | **p-value** |
| --- | --- | --- |
| Trial start year (After 2010 vs. 2010 and before) | 0.56 (0.26, 1.23) | 0.148 |
| Trial end year (After 2010 vs. 2010 and before) | 0.74 (0.52, 1.05) | 0.094 |
| Trial design (Multi-arm parallel vs. 2-arm parallel) | 1.15 (0.71, 1.87) | 0.56 |
| Study drug | | |
| Adalimumab | 1 (reference) | |
| Golimumab | 0.83 (0.56, 1.24) | 0.362 |
| Infliximab | 0.49 (0.22, 1.07) | 0.075 |
| Vedolizumab | 0.71 (0.46, 1.09) | 0.119 |
| Stratification factors (2 vs. 1) | 1.18 (0.75, 1.85) | 0.471 |
| Number of arms (3 vs. 2) | 1.19 (0.68, 2.1) | 0.536 |
| Location | | |
| Asia | 1 (reference) | |
| Multi-continental | 1.28 (0.84, 1.94) | 0.247 |
| North America | 0.85 (0.47, 1.54) | 0.585 |
| Trial setting (Multicentre, single-country vs. Multicentre, multinational) | 0.84 (0.54, 1.29) | 0.419 |
| Route of administration (SC vs. IV) | 1.27 (0.88, 1.83) | 0.209 |
| Blinding | | |
| Double | 1 (reference) | |
| Quadruple | 1.35 (0.98, 1.86) | 0.07 |
| Triple | 0.85 (0.52, 1.38) | 0.51 |
| Time of primary endpoint (> 6 weeks vs. ≤ 6 weeks) | 1.2 (0.84, 1.7) | 0.323 |
| Number of centres (per 10-centre increase) | 1 (0.96, 1.04) | 0.959 |
| Number of follow-up visits | 1 (0.97, 1.03) | 0.97 |
| Abbreviations: CI, confidence interval; IV, intravenous; SC subcutaneous | | |

# **Table S21.** Univariable regression analyses of patient-level factors contributing to endoscopic response (MES ≤ 1) for maintenance trials in ulcerative colitis.

| **Patient Characteristic** | **Odds Ratio (95% CI)** | **p-value** |
| --- | --- | --- |
| Age | 1 (0.98, 1.01) | 0.954 |
| Body mass index | 1.02 (0.98, 1.06) | 0.401 |
| Age at diagnosis | 1 (0.98, 1.02) | 0.71 |
| Disease duration at baseline | 0.99 (0.95, 1.03) | 0.655 |
| Albumin level at baseline (per 10-unit increase) | 2.39 (1.47, 3.88) | <0.001 |
| CRP level at baseline (per 10-unit increase) | 0.93 (0.74, 1.17) | 0.526 |
| FCP level at baseline (per 10-unit increase) | 1 (1, 1) | 0.889 |
| IBDQ total score at baseline (per 10-unit increase) | 1.06 (0.99, 1.14) | 0.086 |
| Mayo score at baseline | 0.91 (0.8, 1.03) | 0.128 |
| Adapted Mayo score at baseline | 0.88 (0.76, 1.01) | 0.071 |
| Sex (Male vs. Female) | 0.81 (0.54, 1.22) | 0.311 |
| Race (White vs. Other) | 0.48 (0.29, 0.82) | 0.007 |
| Prior surgery for IBD (Yes vs. No) | 0.5 (0.14, 1.73) | 0.274 |
| Smoking (Former smoker/Never smoked vs. Current Smoker) | 1.5 (0.65, 3.48) | 0.347 |
| Disease extent based on Montreal criteria at diagnosis | | |
| Extensive UC | 1 (reference) | |
| Left-sided UC | 2.16 (1.2, 3.88) | 0.01 |
| Other | 0.89 (0.48, 1.66) | 0.714 |
| Concomitant 5-ASA drugs at baseline (Yes vs. No) | 1.51 (0.87, 2.61) | 0.14 |
| Previous 5-ASA drug treatment (Yes vs. No) | 1.1 (0.5, 2.39) | 0.816 |
| Concomitant calcineurin inhibitor at baseline (Yes vs. No) | 0.73 (0.08, 6.9) | 0.786 |
| Previous calcineurin inhibitor treatment (Yes vs. No) | 1.33 (0.26, 6.89) | 0.734 |
| Concomitant 6-MP at baseline (Yes vs. No) | 1.43 (0.59, 3.44) | 0.425 |
| Previous 6-MP treatment (Yes vs. No) | 0.94 (0.29, 3.02) | 0.917 |
| Concomitant AZA at baseline (Yes vs. No) | 1.1 (0.6, 2.02) | 0.757 |
| Previous AZA treatment (Yes vs. No) | 1.1 (0.54, 2.21) | 0.797 |
| Concomitant MTX at baseline (Yes vs. No) | - | - |
| Previous MTX drug treatment (Yes vs. No) | 0.57 (0.07, 4.58) | 0.595 |
| Concomitant oral corticosteroid at baseline (Yes vs. No) | 0.77 (0.46, 1.29) | 0.318 |
| Previous oral corticosteroid treatment (Yes vs. No) | 0.64 (0.32, 1.26) | 0.198 |
| Prior exposure to anti-TNFs (Yes vs. No) | 0.85 (0.42, 1.69) | 0.635 |
| Prior failure to anti-TNFs (Yes vs. No) | 0.73 (0.31, 1.72) | 0.471 |
| Prior intolerance to anti-TNFs (Yes vs. No) | 0.56 (0.12, 2.6) | 0.459 |
| Previous exposure to biologic (Yes vs. No) | 0.85 (0.42, 1.69) | 0.635 |
| Abbreviations: Abbreviations: CI, confidence interval; MES, Mayo endoscopic subscore; SF, stool frequency; RB, rectal bleeding; IBDQ, inflammatory bowel disease questionnaire; IBD, inflammatory bowel disease; CRP, C-reactive protein; FCP, fecal calprotectin; 5-ASA, 5-aminosalicylic acid; 6-MP, 6-mercaptopurine; AZA, azathioprine; MTX, methotrexate; TNF, tumor necrosis factor. | | |

# **Table S22.** Univariable regression analyses of trial-level factors contributing to endoscopic response (MES ≤ 1) for maintenance trials in ulcerative colitis.

| **Trial Characteristic** | **Odds Ratio (95% CI)** | **p-value** |
| --- | --- | --- |
| Trial start year (After 2010 vs. 2010 and before) | 1.38 (0.56, 3.42) | 0.487 |
| Trial end year (After 2010 vs. 2010 and before) | 1.74 (0.71, 4.29) | 0.226 |
| Trial design (Multi-arm parallel vs. 2-arm parallel) | 1.12 (0.47, 2.66) | 0.802 |
| Study drug | | |
| Adalimumab | 1 (reference) | |
| Golimumab | 1.09 (0.35, 3.41) | 0.884 |
| Infliximab | 2.74 (1.16, 6.5) | 0.222 |
| Vedolizumab | 1.9 (1.01, 3.57) | 0.047 |
| Stratification factors (2 vs. 1) | 0.41 (0.26, 0.66) | <0.001 |
| Number of arms (3 vs. 2) | 1.12 (0.47, 2.66) | 0.802 |
| Location | | |
| Asia | 1 (reference) | |
| Multi-continental | 0.54 (0.18, 1.6) | 0.268 |
| North America | 0.78 (0.26, 2.38) | 0.669 |
| Trial setting (Multicentre, single-country vs. Multicentre, multinational) | 1.56 (0.74, 3.3) | 0.247 |
| Route of administration (SC vs. IV) | 0.48 (0.29, 0.81) | 0.005 |
| Blinding | | |
| Double | 1 (reference) | |
| Quadruple | 1.06 (0.34, 3.27) | 0.925 |
| Triple | 0.94 (0.23, 3.83) | 0.926 |
| Time of primary endpoint (> 6 weeks vs. ≤ 6 weeks) | 1.1 (0.35, 3.39) | 0.875 |
| Number of centres (per 10-centre increase) | 0.92 (0.85, 1.01) | 0.088 |
| Number of follow-up visits | 0.97 (0.91, 1.03) | 0.341 |
| Abbreviations: CI, confidence interval; IV, intravenous; SC subcutaneous | | |

# **Table S23.** Univariable regression analyses of patient-level factors contributing to endoscopic remission (MES = 0) for induction trials in ulcerative colitis.

| **Patient Characteristic** | **Odds Ratio (95% CI)** | **p-value** |
| --- | --- | --- |
| Age | 1 (0.98, 1.02) | 0.942 |
| Body mass index | 1.04 (1, 1.09) | 0.051 |
| Age at diagnosis | 1 (0.97, 1.03) | 0.984 |
| Disease duration at baseline | 1.02 (0.97, 1.07) | 0.422 |
| Albumin level at baseline (per 10-unit increase) | 1.87 (0.97, 3.61) | 0.06 |
| CRP level at baseline (per 10-unit increase) | 0.73 (0.46, 1.14) | 0.167 |
| FCP level at baseline (per 10-unit increase) | 1 (1, 1) | 0.969 |
| IBDQ total score at baseline (per 10-unit increase) | 0.99 (0.91, 1.09) | 0.905 |
| Mayo score at baseline | 0.7 (0.59, 0.83) | <0.001 |
| Adapted Mayo score at baseline | 0.67 (0.56, 0.81) | <0.001 |
| Sex (Male vs. Female) | 0.96 (0.57, 1.62) | 0.886 |
| Race (White vs. Other) | 1.43 (0.77, 2.68) | 0.26 |
| Prior surgery for IBD (Yes vs. No) | - | - |
| Smoking (Former smoker/Never smoked vs. Current Smoker) | 0.48 (0.21, 1.09) | 0.081 |
| Disease extent based on Montreal criteria at diagnosis | | |
| Extensive UC | 1 (reference) | |
| Left-sided UC | 1.32 (0.37, 4.69) | 0.665 |
| Other | 0.94 (0.42, 2.07) | 0.872 |
| Concomitant 5-ASA drugs at baseline (Yes vs. No) | 0.93 (0.49, 1.8) | 0.838 |
| Previous 5-ASA drug treatment (Yes vs. No) | 0.77 (0.46, 1.3) | 0.333 |
| Concomitant calcineurin inhibitor at baseline (Yes vs. No) | - | - |
| Previous calcineurin inhibitor treatment (Yes vs. No) | - | - |
| Concomitant 6-MP at baseline (Yes vs. No) | - | - |
| Previous 6-MP treatment (Yes vs. No) | - | - |
| Concomitant AZA at baseline (Yes vs. No) | 0.31 (0.07, 1.3) | 0.109 |
| Previous AZA treatment (Yes vs. No) | 0.68 (0.24, 1.94) | 0.468 |
| Concomitant MTX at baseline (Yes vs. No) | 1.61 (0.2, 12.94) | 0.656 |
| Previous MTX drug treatment (Yes vs. No) | 1.44 (0.18, 11.48) | 0.732 |
| Concomitant oral corticosteroid at baseline (Yes vs. No) | 1.2 (0.65, 2.2) | 0.562 |
| Previous oral corticosteroid treatment (Yes vs. No) | 1.02 (0.52, 2) | 0.958 |
| Prior exposure to anti-TNFs (Yes vs. No) | 0.89 (0.36, 2.18) | 0.793 |
| Prior failure to anti-TNFs (Yes vs. No) | 0.71 (0.21, 2.34) | 0.569 |
| Prior intolerance to anti-TNFs (Yes vs. No) | 1.27 (0.16, 10.06) | 0.823 |
| Previous exposure to biologic (Yes vs. No) | 0.89 (0.36, 2.18) | 0.793 |
| Abbreviations: Abbreviations: CI, confidence interval; MES, Mayo endoscopic subscore; SF, stool frequency; RB, rectal bleeding; IBDQ, inflammatory bowel disease questionnaire; IBD, inflammatory bowel disease; CRP, C-reactive protein; FCP, fecal calprotectin; 5-ASA, 5-aminosalicylic acid; 6-MP, 6-mercaptopurine; AZA, azathioprine; MTX, methotrexate; TNF, tumor necrosis factor. | | |

# **Table S24.** Univariable regression analyses of trial-level factors contributing to endoscopic remission (MES = 0) for induction trials in ulcerative colitis.

| **Trial Characteristic** | **Odds Ratio (95% CI)** | **p-value** |
| --- | --- | --- |
| Trial start year (After 2010 vs. 2010 and before) | 0.98 (0.25, 3.8) | 0.972 |
| Trial end year (After 2010 vs. 2010 and before) | 1.28 (0.29, 5.75) | 0.745 |
| Trial design (Multi-arm parallel vs. 2-arm parallel) | 1.2 (0.36, 3.98) | 0.76 |
| Study drug | | |
| Adalimumab | 1 (reference) | |
| Golimumab | 2.3 (0.36, 14.91) | 0.381 |
| Infliximab | 1.01 (0.15, 7.03) | 0.988 |
| Vedolizumab | 2.47 (0.63, 9.66) | 0.195 |
| Stratification factors (2 vs. 1) | 0.53 (0.2, 1.37) | 0.19 |
| Number of arms (3 vs. 2) | 1.2 (0.36, 3.98) | 0.76 |
| Location | | |
| Asia | 1 (reference) | |
| Multi-continental | 0.75 (0.12, 4.47) | 0.748 |
| North America | 0.77 (0.12, 4.86) | 0.779 |
| Trial setting (Multicentre, single-country vs. Multicentre, multinational) | 1.37 (0.42, 4.48) | 0.608 |
| Route of administration (SC vs. IV) | 0.63 (0.21, 1.9) | 0.41 |
| Blinding | | |
| Double | 1 (reference) | |
| Quadruple | 2.02 (0.53, 7.79) | 0.305 |
| Triple | 1.15 (0.21, 6.3) | 0.868 |
| Time of primary endpoint (> 6 weeks vs. ≤ 6 weeks) | 1.23 (0.26, 5.84) | 0.795 |
| Number of centres (per 10-centre increase) | 0.98 (0.82, 1.17) | 0.821 |
| Number of follow-up visits | 0.99 (0.89, 1.1) | 0.85 |
| Abbreviations: CI, confidence interval; IV, intravenous; SC subcutaneous | | |

# **Table S25.** Univariable regression analyses of patient-level factors contributing to endoscopic remission (MES = 0) for maintenance trials in ulcerative colitis.

| **Patient Characteristic** | **Odds Ratio (95% CI)** | **p-value** |
| --- | --- | --- |
| Age | 1.01 (0.99, 1.03) | 0.288 |
| Body mass index | 1.03 (0.98, 1.09) | 0.247 |
| Age at diagnosis | 1.01 (0.98, 1.04) | 0.481 |
| Disease duration at baseline | 0.99 (0.94, 1.05) | 0.836 |
| Albumin level at baseline (per 10-unit increase) | 2.33 (1.18, 4.6) | 0.015 |
| CRP level at baseline (per 10-unit increase) | 1.01 (0.78, 1.3) | 0.958 |
| FCP level at baseline (per 10-unit increase) | 1 (0.99, 1) | 0.185 |
| IBDQ total score at baseline (per 10-unit increase) | 1.03 (0.93, 1.14) | 0.557 |
| Mayo score at baseline | 0.99 (0.83, 1.17) | 0.877 |
| Adapted Mayo score at baseline | 0.96 (0.79, 1.16) | 0.689 |
| Sex (Male vs. Female) | 0.92 (0.52, 1.62) | 0.781 |
| Race (White vs. Other) | 0.98 (0.4, 2.38) | 0.959 |
| Prior surgery for IBD (Yes vs. No) | 1.61 (0.44, 5.93) | 0.476 |
| Smoking (Former smoker/Never smoked vs. Current Smoker) | 1.96 (0.57, 6.77) | 0.287 |
| Disease extent based on Montreal criteria at diagnosis | | |
| Extensive UC | 1 (reference) | |
| Left-sided UC | 3.89 (1.69, 8.95) | 0.001 |
| Other | 1.15 (0.45, 2.96) | 0.771 |
| Concomitant 5-ASA drugs at baseline (Yes vs. No) | 1.83 (0.79, 4.2) | 0.156 |
| Previous 5-ASA drug treatment (Yes vs. No) | 0.88 (0.27, 2.84) | 0.825 |
| Concomitant calcineurin inhibitor at baseline (Yes vs. No) | 1.65 (0.17, 16.11) | 0.667 |
| Previous calcineurin inhibitor treatment (Yes vs. No) | 1.16 (0.13, 10.16) | 0.892 |
| Concomitant 6-MP at baseline (Yes vs. No) | 2.57 (0.93, 7.12) | 0.069 |
| Previous 6-MP treatment (Yes vs. No) | 1.56 (0.4, 6.03) | 0.517 |
| Concomitant AZA at baseline (Yes vs. No) | 1.05 (0.45, 2.42) | 0.91 |
| Previous AZA treatment (Yes vs. No) | 0.89 (0.34, 2.37) | 0.822 |
| Concomitant MTX at baseline (Yes vs. No) | - | - |
| Previous MTX drug treatment (Yes vs. No) | 1.3 (0.16, 10.56) | 0.808 |
| Concomitant oral corticosteroid at baseline (Yes vs. No) | 0.47 (0.23, 0.94) | 0.032 |
| Previous oral corticosteroid treatment (Yes vs. No) | 0.43 (0.17, 1.07) | 0.07 |
| Prior exposure to anti-TNFs (Yes vs. No) | 1.17 (0.48, 2.84) | 0.726 |
| Prior failure to anti-TNFs (Yes vs. No) | 0.75 (0.24, 2.28) | 0.61 |
| Prior intolerance to anti-TNFs (Yes vs. No) | 1.41 (0.29, 6.78) | 0.67 |
| Previous exposure to biologic (Yes vs. No) | 1.17 (0.48, 2.84) | 0.726 |
| Abbreviations: Abbreviations: CI, confidence interval; MES, Mayo endoscopic subscore; SF, stool frequency; RB, rectal bleeding; IBDQ, inflammatory bowel disease questionnaire; IBD, inflammatory bowel disease; CRP, C-reactive protein; FCP, fecal calprotectin; 5-ASA, 5-aminosalicylic acid; 6-MP, 6-mercaptopurine; AZA, azathioprine; MTX, methotrexate; TNF, tumor necrosis factor. | | |

# **Table S26.** Univariable regression analyses of trial-level factors contributing to endoscopic remission (MES = 0) for maintenance trials in ulcerative colitis.

| **Trial Characteristic** | **Odds Ratio (95% CI)** | **p-value** |
| --- | --- | --- |
| Trial start year (After 2010 vs. 2010 and before) | 0.98 (0.25, 3.8) | 0.972 |
| Trial end year (After 2010 vs. 2010 and before) | 1.28 (0.29, 5.75) | 0.745 |
| Trial design (Multi-arm parallel vs. 2-arm parallel) | 1.2 (0.36, 3.98) | 0.76 |
| Study drug | | |
| Adalimumab | 1 (reference) | |
| Golimumab | 2.3 (0.36, 14.91) | 0.381 |
| Infliximab | 1.01 (0.15, 7.03) | 0.988 |
| Vedolizumab | 2.47 (0.63, 9.66) | 0.195 |
| Stratification factors (2 vs. 1) | 0.53 (0.2, 1.37) | 0.19 |
| Number of arms (3 vs. 2) | 1.2 (0.36, 3.98) | 0.76 |
| Location | | |
| Asia | 1 (reference) | |
| Multi-continental | 0.75 (0.12, 4.47) | 0.748 |
| North America | 0.77 (0.12, 4.86) | 0.799 |
| Trial setting (Multicentre, single-country vs. Multicentre, multinational) | 1.37 (0.42, 4.48) | 0.608 |
| Route of administration (SC vs. IV) | 0.63 (0.21, 1.9) | 0.41 |
| Blinding | | |
| Double | 1 (reference) | |
| Quadruple | 2.02 (0.53, 7.79) | 0.305 |
| Triple | 1.15 (0.21, 6.3) | 0.868 |
| Time of primary endpoint (> 6 weeks vs. ≤ 6 weeks) | 1.23 (0.26, 5.84) | 0.795 |
| Number of centres (per 10-centre increase) | 0.98 (0.82, 1.17) | 0.821 |
| Number of follow-up visits | 0.99 (0.89, 1.1) | 0.85 |
| Abbreviations: CI, confidence interval; IV, intravenous; SC subcutaneous | | |

# **Table S27.** Univariable regression analyses of patient-level factors contributing to sustained clinical remission (trial definition) in ulcerative colitis.

| **Patient Characteristic** | **Odds Ratio (95% CI)** | **p-value** |
| --- | --- | --- |
| Age | 1.03 (1, 1.07) | 0.089 |
| Body mass index | 1.07 (0.97, 1.18) | 0.168 |
| Age at diagnosis | 1.02 (0.97, 1.08) | 0.396 |
| Disease duration at baseline | 1.03 (0.93, 1.15) | 0.526 |
| Albumin level at baseline (per 10-unit increase) | 5.96 (1.35, 26.24) | 0.018 |
| CRP level at baseline (per 10-unit increase) | 0.84 (0.42, 1.67) | 0.618 |
| FCP level at baseline (per 10-unit increase) | 1 (0.98, 1.02) | 0.852 |
| IBDQ total score at baseline (per 10-unit increase) | 1.13 (0.94, 1.37) | 0.191 |
| Mayo score at baseline | 0.68 (0.49, 0.96) | 0.03 |
| Adapted Mayo score at baseline | 0.75 (0.54, 1.06) | 0.1 |
| Sex (Male vs. Female) | 0.58 (0.21, 1.57) | 0.281 |
| Race (White vs. Other) | 1.17 (0.06, 24.73) | 0.919 |
| Prior surgery for IBD (Yes vs. No) | - | - |
| Smoking (Former smoker/Never smoked vs. Current Smoker) | 1.59 (0.29, 8.79) | 0.596 |
| Disease extent based on Montreal criteria at diagnosis | | |
| Extensive UC | 1 (reference) | |
| Left-sided UC | 1.96 (0.41, 9.3) | 0.396 |
| Other | 0.44 (0.04, 4.7) | 0.493 |
| Concomitant 5-ASA drugs at baseline (Yes vs. No) | 1.04 (0.21, 5.19) | 0.964 |
| Previous 5-ASA drug treatment (Yes vs. No) | 0.32 (0.05, 2.1) | 0.233 |
| Concomitant calcineurin inhibitor at baseline (Yes vs. No) | - | - |
| Previous calcineurin inhibitor treatment (Yes vs. No) | - | - |
| Concomitant 6-MP at baseline (Yes vs. No) | 2.17 (0.43, 11.03) | 0.35 |
| Previous 6-MP treatment (Yes vs. No) | 3.77 (0.6, 23.46) | 0.155 |
| Concomitant AZA at baseline (Yes vs. No) | 0.68 (0.16, 2.77) | 0.586 |
| Previous AZA treatment (Yes vs. No) | 0.71 (0.13, 3.96) | 0.694 |
| Concomitant MTX at baseline (Yes vs. No) | - | - |
| Previous MTX drug treatment (Yes vs. No) | - | - |
| Concomitant oral corticosteroid at baseline (Yes vs. No) | 0.16 (0.03, 0.82) | 0.027 |
| Previous oral corticosteroid treatment (Yes vs. No) | 0.42 (0.1, 1.7) | 0.223 |
| Prior exposure to anti-TNFs (Yes vs. No) | 0.26 (0.03, 2.32) | 0.229 |
| Prior failure to anti-TNFs (Yes vs. No) | 0.28 (0.03, 2.46) | 0.248 |
| Prior intolerance to anti-TNFs (Yes vs. No) | 3.1 (0.29, 33.45) | 0.35 |
| Previous exposure to biologic (Yes vs. No) | 0.26 (0.03, 2.32) | 0.229 |
| Abbreviations: Abbreviations: CI, confidence interval; MES, Mayo endoscopic subscore; SF, stool frequency; RB, rectal bleeding; IBDQ, inflammatory bowel disease questionnaire; IBD, inflammatory bowel disease; CRP, C-reactive protein; FCP, fecal calprotectin; 5-ASA, 5-aminosalicylic acid; 6-MP, 6-mercaptopurine; AZA, azathioprine; MTX, methotrexate; TNF, tumor necrosis factor. | | |

# **Table S28.** Univariable regression analyses of trial-level factors contributing to sustained clinical remission (trial definition) in ulcerative colitis.

| **Trial Characteristic** | **Odds Ratio (95% CI)** | **p-value** |
| --- | --- | --- |
| Trial start year (After 2010 vs. 2010 and before) | 2.48 (0.16, 39.42) | 0.50. |
| Trial end year (After 2010 vs. 2010 and before) | 1.25 (0.04, 36.78) | 0.898 |
| Trial design (Multi-arm parallel vs. 2-arm parallel) | 0.52 (0.03, 8.25) | 0.646 |
| Study drug | | |
| Adalimumab | 1 (reference) | |
| Golimumab | 10.56 (0.18, 606.3) | 0.254 |
| Infliximab | 0.72 (0.01, 87.7) | 0.893 |
| Vedolizumab | 2.38 (0.07, 80) | 0.628 |
| Stratification factors (2 vs. 1) | 0.67 (0.04, 12.29) | 0.646 |
| Number of arms (3 vs. 2) | 0.52 (0.03, 8.25) | 0.787 |
| Location | | |
| Asia | 1 (reference) | |
| Multi-continental | 0.5 (0.03, 9.24) | 0.638 |
| North America | 0.08 (0, 4.08) | 0.207 |
| Trial setting (Multicentre, single-country vs. Multicentre, multinational) | 3.71 (0.34, 40.36) | 0.282 |
| Route of administration (SC vs. IV) | 1.38 (0.09, 21.92) | 0.819 |
| Blinding | | |
| Double | 1 (reference) | |
| Quadruple | 1.77 (0.11, 28.89) | 0.688 |
| Triple | 0.13 (0, 9.92) | 0.357 |
| Time of primary endpoint (> 6 weeks vs. ≤ 6 weeks) | 11.33 (0.3, 420.7) | 0.188 |
| Number of centres (per 10-centre increase) | 0.88 (0.61, 1.27) | 0.484 |
| Number of follow-up visits | 1.01 (0.79, 1.28) | 0.96 |
| Abbreviations: CI, confidence interval; IV, intravenous; SC subcutaneous | | |

# **Table S29.** Univariable regression analyses of patient-level factors contributing to sustained clinical remission (MES ≤ 1, a ≥ 1-point decrease in SF to achieve a SF ≤ 1, and RB = 0) in ulcerative colitis.

| **Patient Characteristic** | **Odds Ratio (95% CI)** | **p-value** |
| --- | --- | --- |
| Age | 1.04 (1, 1.08) | 0.071 |
| Body mass index | 1.09 (1, 1.2) | 0.062 |
| Age at diagnosis | 1.04 (0.98, 1.11) | 0.216 |
| Disease duration at baseline | 1.05 (0.93, 1.18) | 0.426 |
| Albumin level at baseline (per 10-unit increase) | 5.87 (1.3, 26.44) | 0.021 |
| CRP level at baseline (per 10-unit increase) | 0.95 (0.55, 1.65) | 0.865 |
| FCP level at baseline (per 10-unit increase) | 1 (0.99, 1.01) | 0.985 |
| IBDQ total score at baseline (per 10-unit increase) | 1.11 (0.92, 1.34) | 0.282 |
| Mayo score at baseline | 0.75 (0.53, 1.04) | 0.087 |
| Adapted Mayo score at baseline | 0.79 (0.56, 1.13) | 0.199 |
| Sex (Male vs. Female) | 1.01 (0.35, 2.92) | 0.981 |
| Race (White vs. Other) | 1.44 (0.11, 18.78) | 0.781 |
| Prior surgery for IBD (Yes vs. No) | - | - |
| Smoking (Former smoker/Never smoked vs. Current Smoker) | 0.78 (0.15, 4.06) | 0.769 |
| Disease extent based on Montreal criteria at diagnosis | | |
| Extensive UC | 1 (reference) | |
| Left-sided UC | 0.89 (0.14, 5.53) | 0.902 |
| Other | 0.29 (0.03, 2.81) | 0.285 |
| Concomitant 5-ASA drugs at baseline (Yes vs. No) | 0.56 (0.1, 3.1) | 0.505 |
| Previous 5-ASA drug treatment (Yes vs. No) | 0.18 (0.02, 1.4) | 0.102 |
| Concomitant calcineurin inhibitor at baseline (Yes vs. No) | - | - |
| Previous calcineurin inhibitor treatment (Yes vs. No) | - | - |
| Concomitant 6-MP at baseline (Yes vs. No) | 6.66 (1.22, 36.31) | 0.029 |
| Previous 6-MP treatment (Yes vs. No) | 6.27 (0.91, 43.2) | 0.062 |
| Concomitant AZA at baseline (Yes vs. No) | 0.78 (0.14, 4.23) | 0.772 |
| Previous AZA treatment (Yes vs. No) | 0.42 (0.04, 4) | 0.454 |
| Concomitant MTX at baseline (Yes vs. No) | - | - |
| Previous MTX drug treatment (Yes vs. No) | - | - |
| Concomitant oral corticosteroid at baseline (Yes vs. No) | 0.27 (0.05, 1.4) | 0.118 |
| Previous oral corticosteroid treatment (Yes vs. No) | 0.41 (0.08, 2.2) | 0.299 |
| Prior exposure to anti-TNFs (Yes vs. No) | 0.43 (0.05, 3.99) | 0.456 |
| Prior failure to anti-TNFs (Yes vs. No) | 0.47 (0.05, 4.58) | 0.518 |
| Prior intolerance to anti-TNFs (Yes vs. No) | 4.55 (0.42, 49.81) | 0.214 |
| Previous exposure to biologic (Yes vs. No) | 0.43 (0.05, 3.99) | 0.456 |
| Abbreviations: Abbreviations: CI, confidence interval; MES, Mayo endoscopic subscore; SF, stool frequency; RB, rectal bleeding; IBDQ, inflammatory bowel disease questionnaire; IBD, inflammatory bowel disease; CRP, C-reactive protein; FCP, fecal calprotectin; 5-ASA, 5-aminosalicylic acid; 6-MP, 6-mercaptopurine; AZA, azathioprine; MTX, methotrexate; TNF, tumor necrosis factor. | | |

# **Table S30.** Univariable regression analyses of trial-level factors contributing to sustained clinical remission (MES ≤ 1, a ≥ 1-point decrease in SF to achieve a SF ≤ 1, and RB = 0) in ulcerative colitis.

| **Trial Characteristic** | **Odds Ratio (95% CI)** | **p-value** |
| --- | --- | --- |
| Trial start year (After 2010 vs. 2010 and before) | 1.37 (0.15, 12.52) | 0.782 |
| Trial end year (After 2010 vs. 2010 and before) | 0.78 (0.06, 9.72) | 0.846 |
| Trial design (Multi-arm parallel vs. 2-arm parallel) | 0.63 (0.07, 5.42) | 0.677 |
| Study drug | | |
| Adalimumab | 1 (reference) | |
| Golimumab | 3.93 (0.08, 192.99) | 0.491 |
| Infliximab | 0.58 (0.01, 55.48) | 0.813 |
| Vedolizumab | 1.55 (0.06, 42.02) | 0.794 |
| Stratification factors (2 vs. 1) | 0.53 (0.18, 1.54) | 0.243 |
| Number of arms (3 vs. 2) | 0.63 (0.07, 5.42) | 0.677 |
| Location | | |
| Asia | 1 (reference) | |
| Multi-continental | 0.78 (0.13, 4.57) | 0.781 |
| North America | 0.09 (0, 2.28) | 0.145 |
| Trial setting (Multicentre, single-country vs. Multicentre, multinational) | 1.91 (0.28, 13.17) | 0.511 |
| Route of administration (SC vs. IV) | 1.19 (0.15, 9.72) | 0.871 |
| Blinding | | |
| Double | 1 (reference) | |
| Quadruple | 1.93 (0.33, 11.13) | 0.463 |
| Triple | 0.15 (0.01, 4.34) | 0.271 |
| Time of primary endpoint (> 6 weeks vs. ≤ 6 weeks) | 10.78 (0.58, 199.43) | 0.11 |
| Number of centres (per 10-centre increase) | 0.95 (0.71, 1.26) | 0.708 |
| Number of follow-up visits | 0.98 (0.82, 1.19) | 0.862 |
| Abbreviations: CI, confidence interval; IV, intravenous; SC subcutaneous | | |

# **Table S31.** Univariable regression analyses of patient-level factors contributing to corticosteroid-free clinical remission (trial definition) for induction trials in ulcerative colitis.

| **Patient Characteristic** | **Odds Ratio (95% CI)** | **p-value** |
| --- | --- | --- |
| Age | 1.01 (0.99, 1.03) | 0.481 |
| Body mass index | 1.03 (0.98, 1.08) | 0.231 |
| Age at diagnosis | - | - |
| Disease duration at baseline | 1.06 (1, 1.12) | 0.044 |
| Albumin level at baseline (per 10-unit increase) | 1.7 (0.86, 3.37) | 0.126 |
| CRP level at baseline (per 10-unit increase) | 0.42 (0.09, 1.94) | 0.267 |
| FCP level at baseline (per 10-unit increase) | - | - |
| IBDQ total score at baseline (per 10-unit increase) | 1.08 (0.98, 1.2) | 0.137 |
| Mayo score at baseline | 0.71 (0.59, 0.85) | <0.001 |
| Adapted Mayo score at baseline | 0.7 (0.57, 0.85) | <0.001 |
| Sex (Male vs. Female) | 0.82 (0.46, 1.44) | 0.487 |
| Race (White vs. Other) | 1.81 (0.68, 4.85) | 0.237 |
| Prior surgery for IBD (Yes vs. No) | - | - |
| Smoking (Former smoker/Never smoked vs. Current Smoker) | 1 (0.3, 3.31) | 0.995 |
| Disease extent based on Montreal criteria at diagnosis | | |
| Extensive UC | 1 (reference) | |
| Left-sided UC | 1.85 (0.64, 5.37) | 0.257 |
| Other | 1.11 (0.48, 2.58) | 0.81 |
| Concomitant 5-ASA drugs at baseline (Yes vs. No) | 0.34 (0.14, 0.82) | 0.016 |
| Previous 5-ASA drug treatment (Yes vs. No) | 0.16 (0.07, 0.36) | <0.001 |
| Concomitant calcineurin inhibitor at baseline (Yes vs. No) | - | - |
| Previous calcineurin inhibitor treatment (Yes vs. No) | - | - |
| Concomitant 6-MP at baseline (Yes vs. No) | - | - |
| Previous 6-MP treatment (Yes vs. No) | - | - |
| Concomitant AZA at baseline (Yes vs. No) | 0.67 (0.16, 2.79) | 0.582 |
| Previous AZA treatment (Yes vs. No) | 0.31 (0.07, 1.48) | 0.144 |
| Concomitant MTX at baseline (Yes vs. No) | 1.28 (0.16, 10.43) | 0.814 |
| Previous MTX drug treatment (Yes vs. No) | 1.1 (0.14, 8.92) | 0.927 |
| Concomitant oral corticosteroid at baseline (Yes vs. No) | 0.29 (0.12, 0.7) | 0.006 |
| Previous oral corticosteroid treatment (Yes vs. No) | 0.03 (0, 0.19) | <0.001 |
| Prior exposure to anti-TNFs (Yes vs. No) | 0.58 (0.18, 1.93) | 0.377 |
| Prior failure to anti-TNFs (Yes vs. No) | 0.5 (0.11, 2.34) | 0.377 |
| Prior intolerance to anti-TNFs (Yes vs. No) | - | - |
| Previous exposure to biologic (Yes vs. No) | 0.58 (0.18, 1.93) | 0.377 |
| Abbreviations: Abbreviations: CI, confidence interval; MES, Mayo endoscopic subscore; SF, stool frequency; RB, rectal bleeding; IBDQ, inflammatory bowel disease questionnaire; IBD, inflammatory bowel disease; CRP, C-reactive protein; FCP, fecal calprotectin; 5-ASA, 5-aminosalicylic acid; 6-MP, 6-mercaptopurine; AZA, azathioprine; MTX, methotrexate; TNF, tumor necrosis factor. | | |

# **Table S32.** Univariable regression analyses of trial-level factors contributing to corticosteroid-free clinical remission (trial definition) for induction trials in ulcerative colitis.

| **Trial Characteristic** | **Odds Ratio (95% CI)** | **p-value** |
| --- | --- | --- |
| Trial start year (After 2010 vs. 2010 and before) | 0.51 (0.03, 8) | 0.63 |
| Trial end year (After 2010 vs. 2010 and before) | 0.81 (0.16, 4.02) | 0.792 |
| Trial design (Multi-arm parallel vs. 2-arm parallel) | 0.53 (0.09, 3.08) | 0.476 |
| Study drug | | |
| Adalimumab | 1 (reference) | |
| Golimumab | 0.08 (0.02, 0.29) | <0.001 |
| Infliximab | 0.22 (0.03, 1.62) | 0.137 |
| Vedolizumab | 0.57 (0.29, 1.12) | 0.105 |
| Stratification factors (2 vs. 1) | 3.97 (1.57, 10.03) | 0.004 |
| Number of arms (3 vs. 2) | 0.74 (0.27, 2.01) | 0.555 |
| Location | | |
| Asia | 1 (reference) | |
| Multi-continental | 1.56 (0.22, 11.17) | 0.657 |
| North America | 2.24 (0.15, 33.43) | 0.56 |
| Trial setting (Multicentre, single-country vs. Multicentre, multinational) | 0.57 (0.11, 2.94) | 0.5 |
| Route of administration (SC vs. IV) | 1.19 (0.23, 6.09) | 0.833 |
| Blinding | | |
| Double | 1 (reference) | |
| Quadruple | 8.55 (3.03, 24.16) | <0.001 |
| Triple | 5.29 (1.57, 17.84) | 0.007 |
| Time of primary endpoint (> 6 weeks vs. ≤ 6 weeks) | 2.69 (0.6, 11.97) | 0.195 |
| Number of centres (per 10-centre increase) | 0.94 (0.81, 1.09) | 0.403 |
| Number of follow-up visits | 1.04 (0.91, 1.18) | 0.573 |
| Abbreviations: CI, confidence interval; IV, intravenous; SC subcutaneous | | |

# **Table S33.** Univariable regression analyses of patient-level factors contributing to corticosteroid-free clinical remission (MES ≤ 1, a ≥ 1-point decrease in SF to achieve a SF ≤ 1, and RB = 0) for induction trials in ulcerative colitis.

| **Patient Characteristic** | **Odds Ratio (95% CI)** | **p-value** |
| --- | --- | --- |
| Age | 1 (0.98, 1.02) | 0.844 |
| Body mass index | 1.04 (1, 1.09) | 0.055 |
| Age at diagnosis | 0.97 (0.94, 1.01) | 0.106 |
| Disease duration at baseline | 1.05 (1.01, 1.1) | 0.022 |
| Albumin level at baseline (per 10-unit increase) | 1.51 (0.86, 2.65) | 0.15 |
| CRP level at baseline (per 10-unit increase) | - | - |
| FCP level at baseline (per 10-unit increase) | - | - |
| IBDQ total score at baseline (per 10-unit increase) | 1 (0.92, 1.09) | 0.986 |
| Mayo score at baseline | 0.86 (0.75, 1) | 0.05 |
| Adapted Mayo score at baseline | 0.86 (0.73, 1.01) | 0.073 |
| Sex (Male vs. Female) | 0.82 (0.51, 1.32) | 0.407 |
| Race (White vs. Other) | 1.54 (0.66, 3.58) | 0.315 |
| Prior surgery for IBD (Yes vs. No) | 2.74 (0.31, 23.83) | 0.362 |
| Smoking (Former smoker/Never smoked vs. Current Smoker) | 0.86 (0.33, 2.24) | 0.763 |
| Disease extent based on Montreal criteria at diagnosis | | |
| Extensive UC | 1 (reference) | |
| Left-sided UC | 0.65 (0.25, 1.71) | 0.386 |
| Other | 0.67 (0.31, 1.44) | 0.303 |
| Concomitant 5-ASA drugs at baseline (Yes vs. No) | 0.44 (0.23, 0.85) | 0.015 |
| Previous 5-ASA drug treatment (Yes vs. No) | 0.26 (0.1, 0.67) | 0.006 |
| Concomitant calcineurin inhibitor at baseline (Yes vs. No) | - | - |
| Previous calcineurin inhibitor treatment (Yes vs. No) | - | - |
| Concomitant 6-MP at baseline (Yes vs. No) | 2.81 (0.7, 11.26) | 0.145 |
| Previous 6-MP treatment (Yes vs. No) | 1.93 (0.48, 7.72) | 0.352 |
| Concomitant AZA at baseline (Yes vs. No) | 0.56 (0.17, 1.85) | 0.343 |
| Previous AZA treatment (Yes vs. No) | 0.3 (0.09, 0.96) | 0.043 |
| Concomitant MTX at baseline (Yes vs. No) | 1.99 (0.41, 9.68) | 0.393 |
| Previous MTX drug treatment (Yes vs. No) | 1.73 (0.36, 8.34) | 0.492 |
| Concomitant oral corticosteroid at baseline (Yes vs. No) | 0.29 (0.14, 0.6) | <0.001 |
| Previous oral corticosteroid treatment (Yes vs. No) | 0.02 (0, 0.12) | <0.001 |
| Prior exposure to anti-TNFs (Yes vs. No) | 1.05 (0.43, 2.57) | 0.909 |
| Prior failure to anti-TNFs (Yes vs. No) | 1.28 (0.48, 3.44) | 0.626 |
| Prior intolerance to anti-TNFs (Yes vs. No) | 0.82 (0.1, 6.42) | 0.847 |
| Previous exposure to biologic (Yes vs. No) | 1.05 (0.43, 2.57) | 0.909 |
| Abbreviations: Abbreviations: CI, confidence interval; MES, Mayo endoscopic subscore; SF, stool frequency; RB, rectal bleeding; IBDQ, inflammatory bowel disease questionnaire; IBD, inflammatory bowel disease; CRP, C-reactive protein; FCP, fecal calprotectin; 5-ASA, 5-aminosalicylic acid; 6-MP, 6-mercaptopurine; AZA, azathioprine; MTX, methotrexate; TNF, tumor necrosis factor. | | |

# **Table S34.** Univariable regression analyses of trial-level factors contributing to corticosteroid-free clinical remission (MES ≤ 1, a ≥ 1-point decrease in SF to achieve a SF ≤ 1, and RB = 0) for induction trials in ulcerative colitis.

| **Trial Characteristic** | **Odds Ratio (95% CI)** | **p-value** |
| --- | --- | --- |
| Trial start year (After 2010 vs. 2010 and before) | 0.34 (0.02, 6.03) | 0.464 |
| Trial end year (After 2010 vs. 2010 and before) | 1 (0.18, 5.61) | 0.996 |
| Trial design (Multi-arm parallel vs. 2-arm parallel) | 0.7 (0.1, 4.96) | 0.717 |
| Study drug | | |
| Adalimumab | 1 (reference) | |
| Golimumab | 0.08 (0.03, 0.24) | <0.001 |
| Infliximab | 0.15 (0.02, 1.15) | 0.068 |
| Vedolizumab | 0.74 (0.43, 1.27) | 0.282 |
| Stratification factors (2 vs. 1) | 4.25 (1.16, 15.64) | 0.717 |
| Number of arms (3 vs. 2) | 0.99 (0.3, 3.31) | 0.029 |
| Location | | |
| Asia | 1 (reference) | |
| Multi-continental | 1.25 (0.16, 9.85) | 0.832 |
| North America | 2.33 (0.13, 42.34) | 0.568 |
| Trial setting (Multicentre, single-country vs. Multicentre, multinational) | 0.68 (0.12, 3.92) | 0.665 |
| Route of administration (SC vs. IV) | 1.09 (0.19, 6.15) | 0.923 |
| Blinding | | |
| Double | 1 (reference) | |
| Quadruple | 8.63 (3.68, 20.27) | <0.001 |
| Triple | 6.07 (2.26, 16.29) | <0.001 |
| Time of primary endpoint (> 6 weeks vs. ≤ 6 weeks) | 2.77 (0.55, 13.9) | 0.216 |
| Number of centres (per 10-centre increase) | 0.94 (0.8, 1.09) | 0.404 |
| Number of follow-up visits | 1.05 (0.93, 1.19) | 0.415 |
| Abbreviations: CI, confidence interval; IV, intravenous; SC subcutaneous | | |

# **Table S35.** Univariable regression analyses of patient-level factors contributing to corticosteroid-free clinical remission (trial definition) for maintenance trials in ulcerative colitis.

| **Patient Characteristic** | **Odds Ratio (95% CI)** | **p-value** |
| --- | --- | --- |
| Age | 1 (0.98, 1.02) | 0.784 |
| Body mass index | 0.99 (0.94, 1.04) | 0.745 |
| Age at diagnosis | 1 (0.98, 1.03) | 0.745 |
| Disease duration at baseline | 0.97 (0.92, 1.03) | 0.352 |
| Albumin level at baseline (per 10-unit increase) | 2.4 (1.22, 4.69) | 0.011 |
| CRP level at baseline (per 10-unit increase) | 1.03 (0.8, 1.33) | 0.823 |
| FCP level at baseline (per 10-unit increase) | 1 (1, 1) | 0.976 |
| IBDQ total score at baseline (per 10-unit increase) | 1.04 (0.94, 1.14) | 0.451 |
| Mayo score at baseline | 0.95 (0.81, 1.11) | 0.495 |
| Adapted Mayo score at baseline | 0.94 (0.79, 1.13) | 0.505 |
| Sex (Male vs. Female) | 0.86 (0.5, 1.47) | 0.58 |
| Race (White vs. Other) | 0.72 (0.29, 1.77) | 0.472 |
| Prior surgery for IBD (Yes vs. No) | 0.9 (0.2, 4.01) | 0.893 |
| Smoking (Former smoker/Never smoked vs. Current Smoker) | 2.42 (0.7, 8.36) | 0.163 |
| Disease extent based on Montreal criteria at diagnosis | | |
| Extensive UC | 1 (reference) | |
| Left-sided UC | 2.96 (1.47, 5.98) | 0.002 |
| Other | 0.93 (0.41, 2.13) | 0.861 |
| Concomitant 5-ASA drugs at baseline (Yes vs. No) | 1.76 (0.56, 5.52) | 0.335 |
| Previous 5-ASA drug treatment (Yes vs. No) | 0.74 (0.26, 2.12) | 0.574 |
| Concomitant calcineurin inhibitor at baseline (Yes vs. No) | - | - |
| Previous calcineurin inhibitor treatment (Yes vs. No) | 1.24 (0.14, 10.7) | 0.846 |
| Concomitant 6-MP at baseline (Yes vs. No) | 1.74 (0.57, 5.31) | 0.331 |
| Previous 6-MP treatment (Yes vs. No) | 1.01 (0.21, 4.8) | 0.992 |
| Concomitant AZA at baseline (Yes vs. No) | 0.81 (0.32, 2.04) | 0.65 |
| Previous AZA treatment (Yes vs. No) | 0.59 (0.2, 1.73) | 0.341 |
| Concomitant MTX at baseline (Yes vs. No) | - | - |
| Previous MTX drug treatment (Yes vs. No) | - | - |
| Concomitant oral corticosteroid at baseline (Yes vs. No) | 0.31 (0.15, 0.62) | <0.001 |
| Previous oral corticosteroid treatment (Yes vs. No) | 0.23 (0.08, 0.66) | 0.006 |
| Prior exposure to anti-TNFs (Yes vs. No) | 0.46 (0.19, 1.13) | 0.092 |
| Prior failure to anti-TNFs (Yes vs. No) | 0.18 (0.04, 0.78) | 0.023 |
| Prior intolerance to anti-TNFs (Yes vs. No) | 0.92 (0.2, 4.34) | 0.92 |
| Previous exposure to biologic (Yes vs. No) | 0.46 (0.19, 1.13) | 0.092 |
| Abbreviations: Abbreviations: CI, confidence interval; MES, Mayo endoscopic subscore; SF, stool frequency; RB, rectal bleeding; IBDQ, inflammatory bowel disease questionnaire; IBD, inflammatory bowel disease; CRP, C-reactive protein; FCP, fecal calprotectin; 5-ASA, 5-aminosalicylic acid; 6-MP, 6-mercaptopurine; AZA, azathioprine; MTX, methotrexate; TNF, tumor necrosis factor. | | |

# **Table S36.** Univariable regression analyses of trial-level factors contributing to corticosteroid-free clinical remission (trial definition) for maintenance trials in ulcerative colitis.

| **Trial Characteristic** | **Odds Ratio (95% CI)** | **p-value** |
| --- | --- | --- |
| Trial start year (After 2010 vs. 2010 and before) | 0.8 (0.13, 4.91) | 0.812 |
| Trial end year (After 2010 vs. 2010 and before) | 1.22 (0.19, 7.93) | 0.835 |
| Trial design (Multi-arm parallel vs. 2-arm parallel) | 1.34 (0.29, 6.14) | 0.705 |
| Study drug | | |
| Adalimumab | 1 (reference) | |
| Golimumab | 2.55 (0.78, 8.29) | 0.12 |
| Infliximab | 0.13 (0.01, 2.36) | 0.17 |
| Vedolizumab | 2.66 (1.28, 5.53) | 0.009 |
| Stratification factors (2 vs. 1) | 0.84 (0.27, 2.63) | 0.766 |
| Number of arms (3 vs. 2) | 1.34 (0.29, 6.14) | 0.705 |
| Location | | |
| Asia | 1 (reference) | |
| Multi-continental | 1.07 (0.09, 13.2) | 0.961 |
| North America | 2.03 (0.16, 25.3) | 0.583 |
| Trial setting (Multicentre, single-country vs. Multicentre, multinational) | 0.75 (0.15, 3.67) | 0.718 |
| Route of administration (SC vs. IV) | 0.53 (0.19, 1.49) | 0.232 |
| Blinding | | |
| Double | 1 (reference) | |
| Quadruple | 2.51 (0.39, 16.23) | 0.333 |
| Triple | 3.04 (0.31, 29.35) | 0.337 |
| Time of primary endpoint (> 6 weeks vs. ≤ 6 weeks) | 0.54 (0.09, 3.37) | 0.514 |
| Number of centres (per 10-centre increase) | 1.08 (0.84, 1.38) | 0.563 |
| Number of follow-up visits | 1 (0.87, 1.15) | 0.996 |
| Abbreviations: CI, confidence interval; IV, intravenous; SC subcutaneous | | |

# **Table S37.** Univariable regression analyses of patient-level factors contributing to corticosteroid-free clinical remission (MES ≤ 1, a ≥ 1-point decrease in SF to achieve a SF ≤ 1, and RB = 0) for maintenance trials in ulcerative colitis.

| **Patient Characteristic** | **Odds Ratio (95% CI)** | **p-value** |
| --- | --- | --- |
| Age | 1 (0.98, 1.02) | 0.953 |
| Body mass index | 0.99 (0.94, 1.04) | 0.733 |
| Age at diagnosis | 1 (0.97, 1.02) | 0.811 |
| Disease duration at baseline | 0.98 (0.93, 1.03) | 0.437 |
| Albumin level at baseline (per 10-unit increase) | 2.96 (1.49, 5.89) | 0.002 |
| CRP level at baseline (per 10-unit increase) | 0.99 (0.76, 1.3) | 0.946 |
| FCP level at baseline (per 10-unit increase) | 1 (1, 1) | 0.844 |
| IBDQ total score at baseline (per 10-unit increase) | 1.04 (0.95, 1.15) | 0.382 |
| Mayo score at baseline | 0.98 (0.84, 1.15) | 0.828 |
| Adapted Mayo score at baseline | 0.96 (0.81, 1.15) | 0.691 |
| Sex (Male vs. Female) | 1.02 (0.6, 1.75) | 0.938 |
| Race (White vs. Other) | 0.66 (0.27, 1.62) | 0.364 |
| Prior surgery for IBD (Yes vs. No) | 1.13 (0.28, 4.52) | 0.863 |
| Smoking (Former smoker/Never smoked vs. Current Smoker) | 1.63 (0.55, 4.82 | 0.378 |
| Disease extent based on Montreal criteria at diagnosis | | |
| Extensive UC | 1 (reference) | |
| Left-sided UC | 1.84 (0.83, 4.05) | 0.132 |
| Other | 0.9 (0.39, 2.1) | 0.816 |
| Concomitant 5-ASA drugs at baseline (Yes vs. No) | 1.37 (0.5, 3.77) | 0.546 |
| Previous 5-ASA drug treatment (Yes vs. No) | 0.72 (0.25, 2.09) | 0.541 |
| Concomitant calcineurin inhibitor at baseline (Yes vs. No) | - | - |
| Previous calcineurin inhibitor treatment (Yes vs. No) | 1.08 (0.12, 9.31) | 0.946 |
| Concomitant 6-MP at baseline (Yes vs. No) | 2.46 (0.86, 7.05) | 0.093 |
| Previous 6-MP treatment (Yes vs. No) | 1.46 (0.38, 5.57) | 0.582 |
| Concomitant AZA at baseline (Yes vs. No) | 1.09 (0.46, 2.58) | 0.849 |
| Previous AZA treatment (Yes vs. No) | 0.81 (0.31, 2.13) | 0.675 |
| Concomitant MTX at baseline (Yes vs. No) | - | - |
| Previous MTX drug treatment (Yes vs. No) | - | - |
| Concomitant oral corticosteroid at baseline (Yes vs. No) | 0.3 (0.15, 0.6) | <0.001 |
| Previous oral corticosteroid treatment (Yes vs. No) | 0.19 (0.07, 0.55) | 0.002 |
| Prior exposure to anti-TNFs (Yes vs. No) | 0.7 (0.3, 1.64) | 0.409 |
| Prior failure to anti-TNFs (Yes vs. No) | 0.54 (0.18, 1.56) | 0.251 |
| Prior intolerance to anti-TNFs (Yes vs. No) | 0.42 (0.05, 3.3) | 0.408 |
| Previous exposure to biologic (Yes vs. No) | 0.7 (0.3, 1.64) | 0.409 |
| Abbreviations: Abbreviations: CI, confidence interval; MES, Mayo endoscopic subscore; SF, stool frequency; RB, rectal bleeding; IBDQ, inflammatory bowel disease questionnaire; IBD, inflammatory bowel disease; CRP, C-reactive protein; FCP, fecal calprotectin; 5-ASA, 5-aminosalicylic acid; 6-MP, 6-mercaptopurine; AZA, azathioprine; MTX, methotrexate; TNF, tumor necrosis factor. | | |

# **Table S38.** Univariable regression analyses of trial-level factors contributing to corticosteroid-free clinical remission (MES ≤ 1, a ≥ 1-point decrease in SF to achieve a SF ≤ 1, and RB = 0) for maintenance trials in ulcerative colitis.

| **Trial Characteristic** | **Odds Ratio (95% CI)** | **p-value** |
| --- | --- | --- |
| Trial start year (After 2010 vs. 2010 and before) | 0.63 (0.11, 3.45) | 0.593 |
| Trial end year (After 2010 vs. 2010 and before) | 1.18 (0.21, 6.58) | 0.847 |
| Trial design (Multi-arm parallel vs. 2-arm parallel) | 1.54 (0.4, 5.99) | 0.534 |
| Study drug | | |
| Adalimumab | 1 (reference) | |
| Golimumab | 1.95 (0.46, 8.17) | 0.362 |
| Infliximab | 0.13 (0.01, 2.54) | 0.18 |
| Vedolizumab | 2.73 (1.08, 6.88) | 0.034 |
| Stratification factors (2 vs. 1) | 0.54 (0.24, 1.24) | 0.534 |
| Number of arms (3 vs. 2) | 1.54 (0.4, 5.99) | 0.148 |
| Location | | |
| Asia | 1 (reference) | |
| Multi-continental | 1.04 (0.1, 10.82) | 0.973 |
| North America | 1.65 (0.16, 17.43) | 0.676 |
| Trial setting (Multicentre, single-country vs. Multicentre, multinational) | 0.84 (0.19, 3.62) | 0.813 |
| Route of administration (SC vs. IV) | 0.49 (0.21, 1.15) | 0.103 |
| Blinding | | |
| Double | 1 (reference) | |
| Quadruple | 2.74 (0.56, 13.3) | 0.212 |
| Triple | 2.6 (0.38, 17.61) | 0.327 |
| Time of primary endpoint (> 6 weeks vs. ≤ 6 weeks) | 0.66 (0.12, 3.73) | 0.636 |
| Number of centres (per 10-centre increase) | 1.07 (0.85, 1.36) | 0.563 |
| Number of follow-up visits | 1 (0.88, 1.14) | 0.994 |
| Abbreviations: CI, confidence interval; IV, intravenous; SC subcutaneous | | |

# **Table S39.** Univariable regression analyses of patient-level factors contributing to sustained corticosteroid-free clinical remission (trial definition) in ulcerative colitis.

| **Patient Characteristic** | **Odds Ratio (95% CI)** | **p-value** |
| --- | --- | --- |
| Age | 1.04 (1, 1.07) | 0.068 |
| Body mass index | 1.06 (0.96, 1.18) | 0.25 |
| Age at diagnosis | 1.03 (0.97, 1.09) | 0.352 |
| Disease duration at baseline | 1.05 (0.94, 1.17) | 0.366 |
| Albumin level at baseline (per 10-unit increase) | 5.04 (1.14, 22.28) | 0.033 |
| CRP level at baseline (per 10-unit increase) | 0.88 (0.46, 1.67) | 0.688 |
| FCP level at baseline (per 10-unit increase) | 1 (0.98, 1.02) | 0.852 |
| IBDQ total score at baseline (per 10-unit increase) | 1.14 (0.94, 1.38) | 0.919 |
| Mayo score at baseline | 0.65 (0.45, 0.93) | 0.017 |
| Adapted Mayo score at baseline | 0.71 (0.5, 1.01) | 0.058 |
| Sex (Male vs. Female) | 0.66 (0.24, 1.82) | 0.418 |
| Race (White vs. Other) | 1.15 (0.06, 22.81) | 0.925 |
| Prior surgery for IBD (Yes vs. No) | - | - |
| Smoking (Former smoker/Never smoked vs. Current Smoker) | 1.49 (0.27, 8.4) | 0.648 |
| Disease extent based on Montreal criteria at diagnosis | | |
| Extensive UC | 1 (reference) | |
| Left-sided UC | 1.43 (0.27, 7.45) | 0.675 |
| Other | 0.43 (0.04, 4.61) | 0.487 |
| Concomitant 5-ASA drugs at baseline (Yes vs. No) | 2.05 (0.32, 13.09) | 0.449 |
| Previous 5-ASA drug treatment (Yes vs. No) | 0.79 (0.08, 7.73) | 0.841 |
| Concomitant calcineurin inhibitor at baseline (Yes vs. No) | - | - |
| Previous calcineurin inhibitor treatment (Yes vs. No) | - | - |
| Concomitant 6-MP at baseline (Yes vs. No) | 1.1 (0.18, 6.89) | 0.918 |
| Previous 6-MP treatment (Yes vs. No) | 1.63 (0.17, 16) | 0.673 |
| Concomitant AZA at baseline (Yes vs. No) | 0.78 (0.19, 3.29) | 0.735 |
| Previous AZA treatment (Yes vs. No) | 0.91 (0.15, 5.32) | 0.912 |
| Concomitant MTX at baseline (Yes vs. No) | - | - |
| Previous MTX drug treatment (Yes vs. No) | - | - |
| Concomitant oral corticosteroid at baseline (Yes vs. No) | 0.91 (0.15, 5.32) | 0.021 |
| Previous oral corticosteroid treatment (Yes vs. No) | 0.28 (0.05, 1.4) | 0.12 |
| Prior exposure to anti-TNFs (Yes vs. No) | 0.32 (0.04, 2.93) | 0.315 |
| Prior failure to anti-TNFs (Yes vs. No) | 0.34 (0.04, 3.16) | 0.345 |
| Prior intolerance to anti-TNFs (Yes vs. No) | 3.73 (0.34, 40.85) | 0.282 |
| Previous exposure to biologic (Yes vs. No) | 0.32 (0.04, 2.93) | 0.315 |
| Abbreviations: Abbreviations: CI, confidence interval; MES, Mayo endoscopic subscore; SF, stool frequency; RB, rectal bleeding; IBDQ, inflammatory bowel disease questionnaire; IBD, inflammatory bowel disease; CRP, C-reactive protein; FCP, fecal calprotectin; 5-ASA, 5-aminosalicylic acid; 6-MP, 6-mercaptopurine; AZA, azathioprine; MTX, methotrexate; TNF, tumor necrosis factor. | | |

# **Table S40.** Univariable regression analyses of trial-level factors contributing to sustained corticosteroid-free clinical remission (trial definition) in ulcerative colitis.

| **Trial Characteristic** | **Odds Ratio (95% CI)** | **p-value** |
| --- | --- | --- |
| Trial start year (After 2010 vs. 2010 and before) | 2.64 (0.19, 36.69) | 0.469 |
| Trial end year (After 2010 vs. 2010 and before) | 1.21 (0.05, 32.09) | 0.908 |
| Trial design (Multi-arm parallel vs. 2-arm parallel) | 0.49 (0.03, 7.01) | 0.599 |
| Study drug | | |
| Adalimumab | 1 (reference) | |
| Golimumab | 10.36 (0.22, 477.37) | 0.232 |
| Infliximab | 0.71 (0.01, 71.58) | 0.883 |
| Vedolizumab | 2.17 (0.08, 61.27) | 0.651 |
| Stratification factors (2 vs. 1) | 0.75 (0.04, 12.87) | 0.599 |
| Number of arms (3 vs. 2) | 0.49 (0.03, 7.01) | 0.843 |
| Location | | |
| Asia | 1 (reference) | |
| Multi-continental | 0.51 (0.03, 8.47) | 0.599 |
| North America | 0.08 (0, 3.86) | 0.203 |
| Trial setting (Multicentre, single-country vs. Multicentre, multinational) | 3.5 (0.35, 35.43) | 0.289 |
| Route of administration (SC vs. IV) | 1.49 (0.1, 21.68) | 0.77 |
| Blinding | | |
| Double | 1 (reference) | |
| Quadruple | 1.58 (0.11, 23.86) | 0.74 |
| Triple | 0.13 (0, 8.91) | 0.342 |
| Time of primary endpoint (> 6 weeks vs. ≤ 6 weeks) | 11 (0.32, 376.52) | 0.183 |
| Number of centres (per 10-centre increase) | 0.88 (0.62, 1.25) | 0.467 |
| Number of follow-up visits | 1.01 (0.8, 1.27) | 0.951 |
| Abbreviations: CI, confidence interval; IV, intravenous; SC subcutaneous | | |

# **Table S41.** Univariable regression analyses of patient-level factors contributing to sustained corticosteroid-free clinical remission (MES ≤ 1, a ≥ 1-point decrease in SF to achieve a SF ≤ 1, and RB = 0) in ulcerative colitis.

| **Patient Characteristic** | **Odds Ratio (95% CI)** | **p-value** |
| --- | --- | --- |
| Age | 1.03 (1, 1.07) | 0.074 |
| Body mass index | 1.07 (0.97, 1.18) | 0.171 |
| Age at diagnosis | 1.05 (0.98, 1.13) | 0.167 |
| Disease duration at baseline | 1.08 (0.95, 1.22) | 0.254 |
| Albumin level at baseline (per 10-unit increase) | 6.27 (1.39, 28.19) | 0.017 |
| CRP level at baseline (per 10-unit increase) | 0.94 (0.51, 1.72) | 0.835 |
| FCP level at baseline (per 10-unit increase) | 1 (0.98, 1.02) | 0.852 |
| IBDQ total score at baseline (per 10-unit increase) | 1.1 (0.9, 1.33) | 0.348 |
| Mayo score at baseline | 0.69 (0.49, 0.96) | 0.029 |
| Adapted Mayo score at baseline | 0.72 (0.51, 1.02) | 0.064 |
| Sex (Male vs. Female) | 1.12 (0.39, 3.19) | 0.83 |
| Race (White vs. Other) | 1.51 (0.09, 26.94) | 0.777 |
| Prior surgery for IBD (Yes vs. No) | - | - |
| Smoking (Former smoker/Never smoked vs. Current Smoker) | 0.72 (0.16, 3.23) | 0.664 |
| Disease extent based on Montreal criteria at diagnosis | | |
| Extensive UC | 1 (reference) | |
| Left-sided UC | 0.43 (0.04, 4.26) | 0.474 |
| Other | 0.29 (0.03, 2.77) | 0.282 |
| Concomitant 5-ASA drugs at baseline (Yes vs. No) | 1.52 (0.24, 9.68) | 0.655 |
| Previous 5-ASA drug treatment (Yes vs. No) | 0.47 (0.04, 5.09) | 0.534 |
| Concomitant calcineurin inhibitor at baseline (Yes vs. No) | - | - |
| Previous calcineurin inhibitor treatment (Yes vs. No) | - | - |
| Concomitant 6-MP at baseline (Yes vs. No) | 1.32 (0.2, 8.82) | 0.777 |
| Previous 6-MP treatment (Yes vs. No) | 2.73 (0.26, 28.73) | 0.402 |
| Concomitant AZA at baseline (Yes vs. No) | 0.57 (0.11, 3.07) | 0.512 |
| Previous AZA treatment (Yes vs. No) | 0.57 (0.06, 5.63) | 0.627 |
| Concomitant MTX at baseline (Yes vs. No) | - | - |
| Previous MTX drug treatment (Yes vs. No) | - | - |
| Concomitant oral corticosteroid at baseline (Yes vs. No) | 0.11 (0.01, 0.96) | 0.046 |
| Previous oral corticosteroid treatment (Yes vs. No) | 0.16 (0.02, 1.39) | 0.097 |
| Prior exposure to anti-TNFs (Yes vs. No) | 0.55 (0.06, 5.35) | 0.604 |
| Prior failure to anti-TNFs (Yes vs. No) | 0.61 (0.06, 6.21) | 0.678 |
| Prior intolerance to anti-TNFs (Yes vs. No) | 5.91 (0.5, 69.75) | 0.158 |
| Previous exposure to biologic (Yes vs. No) | 0.55 (0.06, 5.35) | 0.604 |
| Abbreviations: Abbreviations: CI, confidence interval; MES, Mayo endoscopic subscore; SF, stool frequency; RB, rectal bleeding; IBDQ, inflammatory bowel disease questionnaire; IBD, inflammatory bowel disease; CRP, C-reactive protein; FCP, fecal calprotectin; 5-ASA, 5-aminosalicylic acid; 6-MP, 6-mercaptopurine; AZA, azathioprine; MTX, methotrexate; TNF, tumor necrosis factor. | | |

# **Table S42.** Univariable regression analyses of trial-level factors contributing to sustained corticosteroid-free clinical remission (MES ≤ 1, a ≥ 1-point decrease in SF to achieve a SF ≤ 1, and RB = 0) in ulcerative colitis.

| **Trial Characteristic** | **Odds Ratio (95% CI)** | **p-value** |
| --- | --- | --- |
| Trial start year (After 2010 vs. 2010 and before) | 2.83 (0.3, 26.46) | 0.361 |
| Trial end year (After 2010 vs. 2010 and before) | 0.82 (0.04, 17.31) | 0.901 |
| Trial design (Multi-arm parallel vs. 2-arm parallel) | 0.37 (0.04, 3.74) | 0.397 |
| Study drug | | |
| Adalimumab | 1 (reference) | |
| Golimumab | 8.13 (0.25, 261.6) | 0.237 |
| Infliximab | 0.55 (0.01, 41.89) | 0.789 |
| Vedolizumab | 1.33 (0.06, 28.64) | 0.857 |
| Stratification factors (2 vs. 1) | 1.15 (0.09, 14.91) | 0.397 |
| Number of arms (3 vs. 2) | 0.37 (0.04, 3.74) | 0.915 |
| Location | | |
| Asia | 1 (reference) | |
| Multi-continental | 0.79 (0.06, 10.97) | 0.858 |
| North America | 0.09 (0, 3.98) | 0.215 |
| Trial setting (Multicentre, single-country vs. Multicentre, multinational) | 2.37 (0.23, 24.82) | 0.472 |
| Route of administration (SC vs. IV) | 2.06 (0.19, 22.15) | 0.55 |
| Blinding | | |
| Double | 1 (reference) | |
| Quadruple | 1.32 (0.12, 14.8) | 0.821 |
| Triple | 0.12 (0, 5.99) | 0.284 |
| Time of primary endpoint (> 6 weeks vs. ≤ 6 weeks) | 11.4 (0.45, 290.96) | 0.141 |
| Number of centres (per 10-centre increase) | 0.91 (0.65, 1.26) | 0.559 |
| Number of follow-up visits | 1 (0.8, 1.24) | 0.996 |
| Abbreviations: CI, confidence interval; IV, intravenous; SC subcutaneous | | |

# **Table S43.** Univariable regression analyses of patient-level factors contributing to adverse events for induction trials in ulcerative colitis.

| **Patient Characteristic** | **Odds Ratio (95% CI)** | **p-value** |
| --- | --- | --- |
| Age | 1 (0.99, 1.01) | 0.887 |
| Body mass index | 1.03 (1, 1.06) | 0.024 |
| Age at diagnosis | 1.01 (0.99, 1.02) | 0.328 |
| Disease duration at baseline | 1 (0.97, 1.04) | 0.817 |
| Albumin level at baseline (per 10-unit increase) | 0.57 (0.41, 0.78) | <0.001 |
| CRP level at baseline (per 10-unit increase) | 1.19 (1.04, 1.35) | 0.01 |
| FCP level at baseline (per 10-unit increase) | - | N/A |
| IBDQ total score at baseline (per 10-unit increase) | 0.93 (0.89, 0.97) | 0.001 |
| Mayo score at baseline | 1.14 (1.05, 1.24) | 0.001 |
| Adapted Mayo score at baseline | 1.15 (1.04, 1.26) | 0.004 |
| Sex (Male vs. Female) | 0.74 (0.57, 0.96) | 0.023 |
| Race (White vs. Other) | 0.76 (0.53, 1.1) | 0.151 |
| Prior surgery for IBD (Yes vs. No) | 2.64 (0.23, 30.05) | 0.434 |
| Smoking (Former smoker/Never smoked vs. Current Smoker) | 0.97 (0.56, 1.69) | 0.928 |
| Disease extent based on Montreal criteria at diagnosis | | |
| Extensive UC | 1 (reference) | |
| Left-sided UC | 1.14 (0.67, 1.93) | 0.639 |
| Other | 1.04 (0.64, 1.7) | 0.872 |
| Concomitant 5-ASA drugs at baseline (Yes vs. No) | 1.18 (0.63, 2.2) | 0.609 |
| Previous 5-ASA drug treatment (Yes vs. No) | 0.74 (0.36, 1.5) | 0.399 |
| Concomitant calcineurin inhibitor at baseline (Yes vs. No) | 1.6 (0.37, 7.03) | 0.53 |
| Previous calcineurin inhibitor treatment (Yes vs. No) | 1.1 (0.23, 5.12) | 0.907 |
| Concomitant 6-MP at baseline (Yes vs. No) | 1.21 (0.35, 4.21) | 0.766 |
| Previous 6-MP treatment (Yes vs. No) | 2.07 (0.58, 7.38) | 0.264 |
| Concomitant AZA at baseline (Yes vs. No) | 1.19 (0.59, 2.37) | 0.628 |
| Previous AZA treatment (Yes vs. No) | 1.01 (0.44, 2.31) | 0.981 |
| Concomitant MTX at baseline (Yes vs. No) | 0.29 (0.07, 1.15) | 0.078 |
| Previous MTX drug treatment (Yes vs. No) | 0.35 (0.1, 1.24) | 0.104 |
| Concomitant oral corticosteroid at baseline (Yes vs. No) | 0.79 (0.52, 1.22) | 0.294 |
| Previous oral corticosteroid treatment (Yes vs. No) | 0.84 (0.48, 1.46) | 0.529 |
| Prior exposure to anti-TNFs (Yes vs. No) | 1.46 (0.89, 2.4) | 0.134 |
| Prior failure to anti-TNFs (Yes vs. No) | 1.21 (0.68, 2.14) | 0.521 |
| Prior intolerance to anti-TNFs (Yes vs. No) | 1.54 (0.51, 4.59) | 0.442 |
| Previous exposure to biologic (Yes vs. No) | 1.54 (0.51, 4.59) | 0.134 |
| Abbreviations: Abbreviations: CI, confidence interval; MES, Mayo endoscopic subscore; SF, stool frequency; RB, rectal bleeding; IBDQ, inflammatory bowel disease questionnaire; IBD, inflammatory bowel disease; CRP, C-reactive protein; FCP, fecal calprotectin; 5-ASA, 5-aminosalicylic acid; 6-MP, 6-mercaptopurine; AZA, azathioprine; MTX, methotrexate; TNF, tumor necrosis factor. | | |

# **Table S44.** Univariable regression analyses of trial-level factors contributing to adverse events for induction trials in ulcerative colitis.

| **Trial Characteristic** | **Odds Ratio (95% CI)** | **p-value** |
| --- | --- | --- |
| Trial start year (After 2010 vs. 2010 and before) | 0.86 (0.24, 3.12) | 0.817 |
| Trial end year (After 2010 vs. 2010 and before) | 1.16 (0.48, 2.79) | 0.735 |
| Trial design (Multi-arm parallel vs. 2-arm parallel) | 0.54 (0.25, 1.18) | 0.12 |
| Study drug | | |
| Adalimumab | 1 (reference) | |
| Golimumab | 0.38 (0.15, 0.96) | 0.041 |
| Infliximab | 0.58 (0.17, 1.94) | 0.376 |
| Vedolizumab | 0.8 (0.32, 1.99) | 0.627 |
| Stratification factors (2 vs. 1) | 1.73 (0.84, 3.58) | 0.130 |
| Number of arms (3 vs. 2) | 0.67 (0.33, 1.39) | 0.286 |
| Location | | |
| Asia | 1 (reference) | |
| Multi-continental | 0.86 (0.28, 2.63) | 0.789 |
| North America | 1.01 (0.21, 4.76) | 0.995 |
| Trial setting (Multicentre, single-country vs. Multicentre, multinational) | 1.13 (0.42, 2.99) | 0.811 |
| Route of administration (SC vs. IV) | 1.33 (0.57, 3.08) | 0.509 |
| Blinding | | |
| Double | 1 (reference) | |
| Quadruple | 1.4 (0.48, 4.04) | 0.536 |
| Triple | 1.4 (0.32, 6.12) | 0.659 |
| Time of primary endpoint (> 6 weeks vs. ≤ 6 weeks) | 1.81 (0.89, 3.67) | 0.102 |
| Number of centres (per 10-centre increase) | 0.98 (0.91, 1.06) | 0.664 |
| Number of follow-up visits | 1.06 (0.99, 1.14) | 0.12 |
| Abbreviations: CI, confidence interval; IV, intravenous; SC subcutaneous | | |

# **Table S45.** Univariable regression analyses of patient-level factors contributing to adverse events for maintenance trials in ulcerative colitis.

| **Patient Characteristic** | **Odds Ratio (95% CI)** | **p-value** |
| --- | --- | --- |
| Age | 1.02 (1, 1.03) | 0.051 |
| Body mass index | 1.04 (0.99, 1.09) | 0.088 |
| Age at diagnosis | 1.01 (0.98, 1.04) | 0.499 |
| Disease duration at baseline | 1 (0.95, 1.06) | 0.873 |
| Albumin level at baseline (per 10-unit increase) | 0.95 (0.59, 1.53) | 0.825 |
| CRP level at baseline (per 10-unit increase) | 1.1 (0.75, 1.6) | 0.639 |
| FCP level at baseline (per 10-unit increase) | - | - |
| IBDQ total score at baseline (per 10-unit increase) | 1.05 (0.97, 1.13) | 0.256 |
| Mayo score at baseline | 0.89 (0.79, 1.01) | 0.08 |
| Adapted Mayo score at baseline | 0.88 (0.76, 1.02) | 0.081 |
| Sex (Male vs. Female) | 1.23 (0.81, 1.86) | 0.336 |
| Race (White vs. Other) | 1.17 (0.55, 2.5) | 0.684 |
| Prior surgery for IBD (Yes vs. No) | 0.45 (0.04, 4.93) | 0.516 |
| Smoking (Former smoker/Never smoked vs. Current Smoker) | 0.84 (0.35, 2.02) | 0.694 |
| Disease extent based on Montreal criteria at diagnosis | | |
| Extensive UC | 1 (reference) | |
| Left-sided UC | 0.91 (0.43, 1.91) | 0.795 |
| Other | 1.55 (0.74, 3.25) | 0.25 |
| Concomitant 5-ASA drugs at baseline (Yes vs. No) | 1.25 (0.49, 3.2) | 0.64 |
| Previous 5-ASA drug treatment (Yes vs. No) | 2.53 (1.11, 5.74) | 0.027 |
| Concomitant calcineurin inhibitor at baseline (Yes vs. No) | 1.29 (0.11, 15.29) | 0.839 |
| Previous calcineurin inhibitor treatment (Yes vs. No) | 0.87 (0.09, 8.37) | 0.905 |
| Concomitant 6-MP at baseline (Yes vs. No) | 1 (0.24, 4.21) | 0.966 |
| Previous 6-MP treatment (Yes vs. No) | - | - |
| Concomitant AZA at baseline (Yes vs. No) | 0.93 (0.36, 2.41) | 0.875 |
| Previous AZA treatment (Yes vs. No) | - | - |
| Concomitant MTX at baseline (Yes vs. No) | 0.46 (0.1, 2.13) | 0.323 |
| Previous MTX drug treatment (Yes vs. No) | 0.78 (0.2, 3.1) | 0.721 |
| Concomitant oral corticosteroid at baseline (Yes vs. No) | 1.11 (0.6, 2.06) | 0.728 |
| Previous oral corticosteroid treatment (Yes vs. No) | 0.74 (0.26, 2.09) | 0.569 |
| Prior exposure to anti-TNFs (Yes vs. No) | 1.91 (0.84, 4.35) | 0.124 |
| Prior failure to anti-TNFs (Yes vs. No) | 1.56 (0.56, 4.39) | 0.397 |
| Prior intolerance to anti-TNFs (Yes vs. No) | 1.43 (0.3, 6.74) | 0.652 |
| Previous exposure to biologic (Yes vs. No) | 1.91 (0.84, 4.35) | 0.124 |
| Abbreviations: Abbreviations: CI, confidence interval; MES, Mayo endoscopic subscore; SF, stool frequency; RB, rectal bleeding; IBDQ, inflammatory bowel disease questionnaire; IBD, inflammatory bowel disease; CRP, C-reactive protein; FCP, fecal calprotectin; 5-ASA, 5-aminosalicylic acid; 6-MP, 6-mercaptopurine; AZA, azathioprine; MTX, methotrexate; TNF, tumor necrosis factor. | | |

# **Table S46.** Univariable regression analyses of trial-level factors contributing to adverse events for maintenance trials in ulcerative colitis.

| **Trial Characteristic** | **Odds Ratio (95% CI)** | **p-value** |
| --- | --- | --- |
| Trial start year (After 2010 vs. 2010 and before) | 0.53 (0.05, 6.02) | 0.609 |
| Trial end year (After 2010 vs. 2010 and before) | 1.4 (0.06, 30.02) | 0.831 |
| Trial design (Multi-arm parallel vs. 2-arm parallel) | 2.33 (0.23, 23.5) | 0.474 |
| Study drug | | |
| Adalimumab | 1 (reference) | |
| Golimumab | 5.21 (0.47, 57.37) | 0.178 |
| Infliximab | 0.21 (0.03, 1.56) | 0.126 |
| Vedolizumab | 2.03 (0.36, 11.47) | 0.424 |
| Stratification factors (2 vs. 1) | 2.03 (0.18, 23.19) | 0.569 |
| Number of arms (3 vs. 2) | 2.33 (0.23, 23.5) | 0.474 |
| Location | | |
| Asia | 1 (reference) | |
| Multi-continental | 0.67 (0.01, 35.51) | 0.842 |
| North America | 0.84 (0.02, 45.17) | 0.932 |
| Trial setting (Multicentre, single-country vs. Multicentre, multinational) | 1.29 (0.1, 16.4) | 0.842 |
| Route of administration (SC vs. IV) | 2.11 (0.18, 24.75) | 0.551 |
| Blinding | | |
| Double | 1 (reference) | |
| Quadruple | 2.05 (0.07, 56.27) | 0.672 |
| Triple | 1.36 (0.02, 75.08) | 0.88 |
| Time of primary endpoint (> 6 weeks vs. ≤ 6 weeks) | 1.04 (0.05, 23.05) | 0.978 |
| Number of centres (per 10-centre increase) | 1.08 (0.8, 1.46) | 0.625 |
| Number of follow-up visits | 1.23 (1.08, 1.39) | 0.001 |
| Abbreviations: CI, confidence interval; IV, intravenous; SC subcutaneous | | |

# **Table S47.** Univariable regression analyses of patient-level factors contributing to serious adverse events for induction trials in ulcerative colitis.

| **Patient Characteristic** | **Odds Ratio (95% CI)** | **p-value** |
| --- | --- | --- |
| Age | 1 (0.99, 1.02) | 0.865 |
| Body mass index | 1.03 (1, 1.06) | 0.086 |
| Age at diagnosis | 1 (0.99, 1.02) | 0.637 |
| Disease duration at baseline | 0.99 (0.93, 1.04) | 0.06 |
| Albumin level at baseline (per 10-unit increase) | 0.47 (0.29, 0.74) | 0.001 |
| CRP level at baseline (per 10-unit increase) | 1.14 (0.99, 1.31) | 0.071 |
| FCP level at baseline (per 10-unit increase) | - | - |
| IBDQ total score at baseline (per 10-unit increase) | 0.92 (0.87, 0.97) | 0.004 |
| Mayo score at baseline | 1.24 (1.1, 1.39) | <0.001 |
| Adapted Mayo score at baseline | 1.24 (1.08, 1.42) | 0.002 |
| Sex (Male vs. Female) | 0.62 (0.43, 0.89) | 0.009 |
| Race (White vs. Other) | 0.57 (0.36, 0.9) | 0.016 |
| Prior surgery for IBD (Yes vs. No) | - | - |
| Smoking (Former smoker/Never smoked vs. Current Smoker) | 0.98 (0.46, 2.08) | 0.953 |
| Disease extent based on Montreal criteria at diagnosis | | |
| Extensive UC | 1 (reference) | |
| Left-sided UC | 0.38 (0.11, 1.31) | 0.126 |
| Other | 0.81 (0.34, 1.93) | 0.635 |
| Concomitant 5-ASA drugs at baseline (Yes vs. No) | 0.68 (0.28, 1.63) | 0.384 |
| Previous 5-ASA drug treatment (Yes vs. No) | 2.47 (0.78, 7.88) | 0.125 |
| Concomitant calcineurin inhibitor at baseline (Yes vs. No) | 2.05 (0.24, 17.69) | 0.515 |
| Previous calcineurin inhibitor treatment (Yes vs. No) | - | - |
| Concomitant 6-MP at baseline (Yes vs. No) | - | - |
| Previous 6-MP treatment (Yes vs. No) | - | - |
| Concomitant AZA at baseline (Yes vs. No) | - | - |
| Previous AZA treatment (Yes vs. No) | - | - |
| Concomitant MTX at baseline (Yes vs. No) | 1.56 (0.19, 12.58) | 0.677 |
| Previous MTX drug treatment (Yes vs. No) | 1.35 (0.17, 10.83) | 0.775 |
| Concomitant oral corticosteroid at baseline (Yes vs. No) | 0.51 (0.22, 1.16) | 0.109 |
| Previous oral corticosteroid treatment (Yes vs. No) | 1.9 (0.81, 4.44) | 0.138 |
| Prior exposure to anti-TNFs (Yes vs. No) | 0.26 (0.07, 0.93) | 0.038 |
| Prior failure to anti-TNFs (Yes vs. No) | 0.17 (0.02, 1.28) | 0.085 |
| Prior intolerance to anti-TNFs (Yes vs. No) | - | - |
| Previous exposure to biologic (Yes vs. No) | 0.26 (0.07, 0.93) | 0.038 |
| Abbreviations: Abbreviations: CI, confidence interval; MES, Mayo endoscopic subscore; SF, stool frequency; RB, rectal bleeding; IBDQ, inflammatory bowel disease questionnaire; IBD, inflammatory bowel disease; CRP, C-reactive protein; FCP, fecal calprotectin; 5-ASA, 5-aminosalicylic acid; 6-MP, 6-mercaptopurine; AZA, azathioprine; MTX, methotrexate; TNF, tumor necrosis factor. | | |

# **Table S48.** Univariable regression analyses of trial-level factors contributing to serious adverse events for induction trials in ulcerative colitis.

| **Trial Characteristic** | **Odds Ratio (95% CI)** | **p-value** |
| --- | --- | --- |
| Trial start year (After 2010 vs. 2010 and before) | 0.34 (0.03, 4.4) | 0.406 |
| Trial end year (After 2010 vs. 2010 and before) | 0.27 (0.07, 1.12) | 0.072 |
| Trial design (Multi-arm parallel vs. 2-arm parallel) | 1.92 (0.3, 12.42) | 0.493 |
| Study drug | | |
| Adalimumab | 1 (reference) | |
| Golimumab | 5.65 (2.73, 11.68) | <0.001 |
| Infliximab | 0.53 (0.1, 2.64) | 0.436 |
| Vedolizumab | 0.73 (0.3, 1.8) | 0.497 |
| Stratification factors (2 vs. 1) | 0.46 (0.1, 2.18) | 0.493 |
| Number of arms (3 vs. 2) | 0.68 (0.31, 1.49) | 0.334 |
| Location | | |
| Asia | 1 (reference) | |
| Multi-continental | 4.65 (0.76, 28.32) | 0.095 |
| North America | 1.8 (0.16, 20.74) | 0.639 |
| Trial setting (Multicentre, single-country vs. Multicentre, multinational) | 0.26 (0.05, 1.47) | 0.127 |
| Route of administration (SC vs. IV) | 1.78 (0.33, 9.48) | 0.501 |
| Blinding | | |
| Double | 1 (reference) | |
| Quadruple | 0.5 (0.06, 3.83) | 0.503 |
| Triple | 0.37 (0.02, 6.34) | 0.496 |
| Time of primary endpoint (> 6 weeks vs. ≤ 6 weeks) | 0.24 (0.07, 0.81) | 0.021 |
| Number of centres (per 10-centre increase) | 1.17 (1.07, 1.27) | <0.001 |
| Number of follow-up visits | 0.86 (0.78, 0.94) | 0.002 |
| Abbreviations: CI, confidence interval; IV, intravenous; SC subcutaneous | | |

# **Table S49.** Univariable regression analyses of patient-level factors contributing to serious adverse events for maintenance trials in ulcerative colitis.

| **Patient Characteristic** | **Odds Ratio (95% CI)** | **p-value** |
| --- | --- | --- |
| Age | 1.02 (1, 1.04) | 0.026 |
| Body mass index | 1.02 (0.97, 1.07) | 0.474 |
| Age at diagnosis | 1.03 (1, 1.06) | 0.07 |
| Disease duration at baseline | 0.99 (0.93, 1.05) | 0.713 |
| Albumin level at baseline (per 10-unit increase) | 0.62 (0.36, 1.08) | 0.092 |
| CRP level at baseline (per 10-unit increase) | 0.65 (0.24, 1.75) | 0.396 |
| FCP level at baseline (per 10-unit increase) | 1 (1, 1) | 0.769 |
| IBDQ total score at baseline (per 10-unit increase) | 0.99 (0.92, 1.07) | 0.787 |
| Mayo score at baseline | 1.01 (0.86, 1.18) | 0.94 |
| Adapted Mayo score at baseline | 0.99 (0.83, 1.19) | 0.944 |
| Sex (Male vs. Female) | 1.18 (0.69, 2.02) | 0.554 |
| Race (White vs. Other) | 1.21 (0.69, 2.13) | 0.499 |
| Prior surgery for IBD (Yes vs. No) | 2.2 (0.71, 6.8) | 0.169 |
| Smoking (Former smoker/Never smoked vs. Current Smoker) | 0.77 (0.33, 1.81) | 0.549 |
| Disease extent based on Montreal criteria at diagnosis | | |
| Extensive UC | 1 (reference) | |
| Left-sided UC | 0.8 (0.34, 1.92) | 0.621 |
| Other | 1.03 (0.44, 2.39) | 0.95 |
| Concomitant 5-ASA drugs at baseline (Yes vs. No) | 0.69 (0.36, 1.34) | 0.276 |
| Previous 5-ASA drug treatment (Yes vs. No) | 0.72 (0.29, 1.76) | 0.47 |
| Concomitant calcineurin inhibitor at baseline (Yes vs. No) | - | - |
| Previous calcineurin inhibitor treatment (Yes vs. No) | - | - |
| Concomitant 6-MP at baseline (Yes vs. No) | 0.72 (0.16, 3.14) | 0.658 |
| Previous 6-MP treatment (Yes vs. No) | 2.38 (0.47, 12.12) | 0.295 |
| Concomitant AZA at baseline (Yes vs. No) | 0.67 (0.23, 1.94) | 0.459 |
| Previous AZA treatment (Yes vs. No) | 1 (0.29, 3.47) | 1 |
| Concomitant MTX at baseline (Yes vs. No) | 1.21 (0.14, 10.24) | 0.86 |
| Previous MTX drug treatment (Yes vs. No) | 1.78 (0.37, 8.6) | 0.474 |
| Concomitant oral corticosteroid at baseline (Yes vs. No) | 1.14 (0.66, 1.99) | 0.636 |
| Previous oral corticosteroid treatment (Yes vs. No) | 1.48 (0.68, 3.21) | 0.324 |
| Prior exposure to anti-TNFs (Yes vs. No) | 0.75 (0.33, 1.73) | 0.504 |
| Prior failure to anti-TNFs (Yes vs. No) | 0.71 (0.24, 2.05) | 0.522 |
| Prior intolerance to anti-TNFs (Yes vs. No) | 0.55 (0.07, 4.28) | 0.569 |
| Previous exposure to biologic (Yes vs. No) | 0.75 (0.33, 1.73) | 0.504 |
| Abbreviations: Abbreviations: CI, confidence interval; MES, Mayo endoscopic subscore; SF, stool frequency; RB, rectal bleeding; IBDQ, inflammatory bowel disease questionnaire; IBD, inflammatory bowel disease; CRP, C-reactive protein; FCP, fecal calprotectin; 5-ASA, 5-aminosalicylic acid; 6-MP, 6-mercaptopurine; AZA, azathioprine; MTX, methotrexate; TNF, tumor necrosis factor. | | |

# **Table S50.** Univariable regression analyses of trial-level factors contributing to serious adverse events for maintenance trials in ulcerative colitis.

| **Trial Characteristic** | **Odds Ratio (95% CI)** | **p-value** |
| --- | --- | --- |
| Trial start year (After 2010 vs. 2010 and before) | 1.02 (0.46, 2.29) | 0.953 |
| Trial end year (After 2010 vs. 2010 and before) | 0.99 (0.59, 1.69) | 0.984 |
| Trial design (Multi-arm parallel vs. 2-arm parallel) | 0.98 (0.57, 1.69) | 0.951 |
| Study drug | | |
| Adalimumab | 1 (reference) | |
| Golimumab | 1.66 (0.63, 4.38) | 0.302 |
| Infliximab | 0.3 (0.07, 1.28) | 0.102 |
| Vedolizumab | 0.99 (0.56, 1.74) | 0.964 |
| Stratification factors (2 vs. 1) | 1.71 (0.82, 3.6) | 0.951 |
| Number of arms (3 vs. 2) | 0.98 (0.57, 1.69) | 0.155 |
| Location | | |
| Asia | 1 (reference) | |
| Multi-continental | 1.2 (0.29, 4.93) | 0.801 |
| North America | 1.3 (0.3, 5.55) | 0.726 |
| Trial setting (Multicentre, single-country vs. Multicentre, multinational) | 0.88 (0.47, 1.64) | 0.686 |
| Route of administration (SC vs. IV) | 1.27 (0.69, 2.32) | 0.445 |
| Blinding | | |
| Double | 1 (reference) | |
| Quadruple | 1.01 (0.31, 3.35) | 0.983 |
| Triple | 1.2 (0.31, 4.68) | 0.796 |
| Time of primary endpoint (> 6 weeks vs. ≤ 6 weeks) | 0.89 (0.49, 1.63) | 0.715 |
| Number of centres (per 10-centre increase) | 1.03 (0.94, 1.13) | 0.542 |
| Number of follow-up visits | 1.05 (0.97, 1.14) | 0.204 |
| Abbreviations: CI, confidence interval; IV, intravenous; SC subcutaneous | | |

# **Table S51.** Risk of bias assessment summary of included studies.

| **Study ID** | **Random sequence generation** | **Allocation concealment** | **Blind of participants and personnel** | **Incomplete outcome data** | **Selective reporting** | **Other bias** |
| --- | --- | --- | --- | --- | --- | --- |
| NCT00488774  (PURSUIT-IV) | Low risk | Low risk | Low risk | Low risk | Low risk | Low risk |
| NCT00487539  (PURSUIT-SC) | Low risk | Low risk | Low risk | Low risk | Low risk | Low risk |
| NCT00783718 (GEMINI 1) | Low risk | Low risk | Low risk | Low risk | Low risk | Low risk |
| NCT01863771  (PURSUIT-J) | Low risk | Unclear risk | Low risk | Low risk | Low risk | Low risk |
| NCT00385736  (ULTRA 1) | Low risk | Low risk | Low risk | Low risk | Low risk | Low risk |
| NCT00408629 (ULTRA 2) | Low risk | Low risk | Low risk | Low risk | Low risk | Low risk |
| NCT00853099 | Low risk | Low risk | Low risk | Low risk | Low risk | Low risk |
| NCT02039505 | Low risk | Low risk | Low risk | Low risk | Low risk | Low risk |
| NCT01551290 | Unclear risk | Unclear risk | Low risk | Unclear risk | Low risk | Unclear risk |

# **Figure S1.** Study flow diagram.

Number of additional studies identified through other sources including contact with researchers

**n=8**

=

(n = )

Number of studies identified through database searching **n=15553**

## Identification

## Screening

Number of studies after duplicates removed

**n=9929**

Number of records for which IPD were available on the Vivli and Yale University Open Data (YODA) Access Project data-sharing platforms

**n=9 records (reporting on 12 studies)**

**Number of participants for whom data were provided**

**Induction n=1087; maintenance n=616**

Number of studies excluded, with reasons

**n=520**

Number of studies included in the analysis

**n=9 (reporting on 12 studies)**

Number of participants included in the analysis

**Induction n=1087; maintenance n=616**

Number of studies for which IPD were sought

**n=34**

Number of studies screened for eligibility

**n=554**

## Eligibility

## Obtaining data

## Available data

## Analysed data

# **Figure S2.** Pooled placebo clinical response rates for induction trials.

|  |  |
| --- | --- |
|  |  |
|  |  |

# **Figure S3.** Pooled placebo clinical response rates for maintenance trials.

|  |  |
| --- | --- |
|  |  |
|  |  |

# **Figure S4**. Pooled placebo clinical remission rates for induction trials.

# **Figure S5.** Pooled placebo clinical remission rates for maintenance trials.

# **Figure S6.** Pooled placebo endoscopic response rate (MES ≤ 1) for induction trials.

# **Figure S7.** Pooled placebo endoscopic response rate (MES ≤ 1) for maintenance trials.

# **Figure S8.** Pooled placebo endoscopic remission rate (MES = 0) for induction trials.

# **Figure S9.** Pooled placebo endoscopic remission rate (MES = 0) for maintenance trials.

# **Figure S10.** Pooled placebo sustained clinical remission rate (trial definition).

# **Figure S11.** Pooled placebo sustained clinical remission rate (MES ≤ 1, a ≥ 1-point decrease in SF to achieve a SF ≤ 1, and RB = 0).

# **Figure S12.** Pooled placebo corticosteroid-free clinical remission rate (trial definition) for induction trials.

# **Figure S13.** Pooled placebo corticosteroid-free clinical remission rate (MES ≤ 1, a ≥ 1-point decrease in SF to achieve a SF ≤ 1, and RB = 0) for induction trials.

**Figure S14.** Pooled placebo corticosteroid-free clinical remission rate (trial definition) for maintenance trials.

**Figure S15.** Pooled placebo corticosteroid-free clinical remission rate (MES ≤ 1, a ≥ 1-point decrease in SF to achieve a SF ≤ 1, and RB = 0) for maintenance trials.

**Figure S16.** Pooled placebo sustained corticosteroid-free clinical remission rate (trial definition).

**Figure S17.** Pooled placebo sustained corticosteroid-free clinical remission rate (MES ≤ 1, a ≥ 1-point decrease in SF to achieve a SF ≤ 1, and RB = 0).

**Figure S18.** Pooled placebo adverse event rate for induction trials.

**Figure S19.** Pooled placebo adverse event rate for maintenance trials.

**Figure S20.** Pooled placebo serious adverse event rate for induction trials.

**Figure S21.** Pooled placebo serious adverse event rate for maintenance trials.
